# Supplementary material for: Alterations in acylcarnitines, amines, and lipids inform about the mechanism of action of citalopram/escitalopram in major depression
Source: Transl Psychiatry. 2021 Mar 2;11:153. doi: 10.1038/s41398-020-01097-6 (PMC7925685; doi:10.1038/s41398-020-01097-6)
Supplement: Supplementary file 1 — Supplementary Methods [file 41398_2020_1097_MOESM1_ESM.docx]

**Alterations in Acylcarnitines, Amines, and Lipids Inform about Mechanism of Action of Citalopram/Escitalopram in Major Depression**

Siamak MahmoudianDehkordi PhD, Ahmed T. Ahmed MBBCh, Sudeepa Bhattacharyya PhD, Xianlin Han PhD, Rebecca A.Baillie PhD, [Matthias Arnold](https://www.ncbi.nlm.nih.gov/pubmed/?term=Arnold%20M%5BAuthor%5D&cauthor=true&cauthor_uid=29370177) PhD, Michelle K. Skime, Lisa St. John-Williams MS, M. Arthur Moseley PhD, J. Will Thompson PhD, Gregory Louie MS, Patricio Riva-Posse MD, W. Edward Craighead PhD, William McDonald MD, Ranga Krishnan PhD, A John Rush MD, Mark A. Frye MD, Boadie W. Dunlop MD, Richard M. Weinshilboum MD, The Mood Disorders Precision Medicine Consortium (MDPMC), and Rima Kaddurah-Daouk PhD

**SUPPLEMENTARY MATERIALS**

## Metabolomic Profiling using the Absolute IDQ p180 Kit

***Sample Preparation***:

Samples were prepared using the AbsoluteIDQ® p180 kit (Biocrates Innsbruck, Austria) in strict accordance with their detailed protocol. In brief, after the addition of 10 µL of the supplied internal standard solution to each well of the 96-well extraction plate, 10 µL of each study plasma sample, low/medium/high quality control (QC) samples, blank, zero sample, or calibration standard were added to the appropriate wells. The plate was then dried under a gentle stream of nitrogen. The samples were derivatized with phenyl isothiocyanate then eluted with 5mM ammonium acetate in methanol. Samples were diluted with either 1:1 methanol:water for the UPLC analysis (4:1) or running solvent (a proprietary mixture provided by Biocrates) for flow injection analysis (20:1). A pool of equal volumes of all 76 plasma samples analyzed on the first plate was created, aliquoted, and frozen. The pooled sample was prepared and analyzed in the same way as the study samples on all eight plates. From each plate this sample was injected once before, once during, and once after the study samples in order to measure the performance of the assay across the sample cohort. The analyses of this pool were used to assess and correct potential batch effects.

***Quality Control Samples***: The analysis of the samples using the AbsoluteIDQ® p180 kit was performed using four specific sets of quality controls. First, low/mid/high level quality control (QC) samples provided by Biocrates Life Sciences AG were prepared and analyzed on each plate as recommended by the manufacturer. These QC samples were used for a technical validation of each kit plate. Second, the NIST standard reference material (SRM)-1950 reference plasma was prepared and analyzed twice on each kit plate in order to measure intra- and inter-assay reproducibility. Third, to allow appropriate inter-plate abundance scaling based specifically on this cohort of samples, we generated a Study Pool QC by combining equal volumes of all 76 plasma samples analyzed on the first plate. This sample was frozen in aliquots of an appropriate volume and analyzed independently on all of the eight plates analyzed in this study. The pooled sample was prepared and analyzed twice on each plate, once before, once during and once after the study samples.

***Quantitative UPLC-MS/MS and FIA-MS/MS Analysis***: Sample analysis was performed based on Standard Operating Procedures provided by Biocrates for the AbsoluteIDQ® p180 kit. Chromatographic separation of amino acids and biogenic amines was performed using a ACQUITY UPLC System (Waters Corporation) using a ACQUITY 2.1 mm x 50 mm 1.7 µm BEH C18 column fitted with a ACQUITY BEH C18 1.7 µm VanGuard guard column, and quantified by calibration curve using a linear regression with 1/x^2^ weighting. Acylcarnitines, sphingolipids, and glycerophospholipids, were analyzed by flow injection analysis tandem mass spectrometry (FIA-MS/MS), quantified by internal standard calibration. Thus, FIA-MS/MS analytes are reported as semi-quantitative values except where a stable-isotope labeled internal standard of that exact analyte was used. Samples for both UPLC and FIA were introduced directly into a Xevo TQ-S triple quadrupole mass spectrometer (Waters Corporation) using positive electrospray ionization operating in the Multiple Reaction Monitoring (MRM) mode. MRM transitions (compound-specific precursor to product ion transitions) for each analyte and internal standard were collected over the appropriate retention time using tune files and acquisition methods provided in the AbsoluteIDQ® p180 kit. The UPLC data were imported into TargetLynx (Waters Corporation) for peak integration, calibration and concentration calculations. The UPLC data from TargetLynx^TM^ and FIA data were analyzed using Biocrates’ MetIDQ software. Results of principal component analysis of profiled samples are shown in **Supplementary Fig. 1 to 5**.
