# Supplementary material for: Alterations in acylcarnitines, amines, and lipids inform about the mechanism of action of citalopram/escitalopram in major depression
Source: Transl Psychiatry. 2021 Mar 2;11:153. doi: 10.1038/s41398-020-01097-6 (PMC7925685; doi:10.1038/s41398-020-01097-6)
Supplement: Supplementary file 2 — Supplementary Table 1 [file 41398_2020_1097_MOESM2_ESM.docx]

**Supplementary Table 1. Metabolites Measured by Biocrates P180^®^ Kit**

| Class | Name | Limit of Detection [µM] | Lowest Calibration Standard [µM] | Highest Calibration Standard [µM] | %Missing | SPQC CV | Included in Analysis |
| --- | --- | --- | --- | --- | --- | --- | --- |
| Amino Acids | Alanine | 1 | 20 | 1600 | 0 | 0.05 | Yes |
|  | Arginine | 0.5 | 5 | 400 | 0 | 0.07 | Yes |
|  | Asparagine | 1.5 | 5 | 400 | 0 | 0.05 | Yes |
|  | Aspartate | 1.5 | 5 | 400 | 1.73 | 0.22 | Yes |
|  | Citrulline | 1 | 5 | 400 | 0 | 0.06 | Yes |
|  | Glutamine | 1.5 | 20 | 1600 | 0 | 0.05 | Yes |
|  | Glutamate | 2 | 10 | 800 | 0 | 0.07 | Yes |
|  | Glycine | 0.5 | 25 | 2000 | 0 | 0.05 | Yes |
|  | Histidine | 0.5 | 5 | 400 | 0 | 0.04 | Yes |
|  | Isoleucine | 1.5 | 5 | 400 | 0 | 0.06 | Yes |
|  | Lysine | 0.5 | 10 | 800 | 0 | 0.15 | Yes |
|  | Methionine | 0.1 | 5 | 400 | 0 | 0.09 | Yes |
|  | Ornithine | 0.5 | 5 | 400 | 0 | 0.11 | Yes |
|  | Phenylalanine | 0.1 | 5 | 400 | 0 | 0.04 | Yes |
|  | Proline | 1 | 10 | 800 | 0 | 0.04 | Yes |
|  | Serine | 1 | 5 | 400 | 0 | 0.07 | Yes |
|  | Threonine | 0.5 | 5 | 400 | 0 | 0.04 | Yes |
|  | Tryptophan | 0.5 | 5 | 400 | 0 | 0.04 | Yes |
|  | Tyrosine | 0.5 | 5 | 400 | 0 | 0.04 | Yes |
|  | Valine | 0.5 | 10 | 800 | 0 | 0.07 | Yes |
| Biogenic Amines | Acetylornithine | 0.2 | 10 | 800 | 100 | NA | No |
|  | Asymmetric dimethylarginine | 0.08 | 0.5 | 40 | 0.17 | 0.16 | Yes |
|  | alpha-Aminoadipic acid | 0.4 | 0.25 | 20 | 10.73 | 0.19 | Yes |
|  | Carnosine | 0.1 | 1 | 80 | 99.65 | NA | No |
|  | Creatinine | 1 | 0.5 | 40 | 0 | 0.04 | Yes |
|  | DOPA | 0.17 | 10 | 800 | 51.56 | 0.08 | No |
|  | Dopamine | 0.3 | 0.5 | 40 | 100 | NA | No |
|  | Histamine | 0.3 | 1 | 80 | 99.83 | NA | No |
|  | Kynurenine | 0.3 | 1 | 80 | 0.17 | 0.06 | Yes |
|  | Methioninesulfoxide | 0.3 | 1 | 80 | 3.63 | 0.17 | Yes |
|  | Nitrotyrosine | 0.3 | 1 | 80 | 100 | NA | No |
|  | Phenylethylamine | 0.02 | 1 | 80 | 100 | NA | No |
|  | Putrescine | 0.03 | 0.1 | 8 | 1.73 | 0.14 | Yes |
|  | Sarcosine | 0.3 | 0.1 | 8 | 0 | 0.06 | Yes |
|  | Symmetric dimethylarginine | 0.03 | 1 | 80 | 0 | 0.07 | Yes |
|  | Serotonin | 0.03 | 0.1 | 8 | 38.93 | 0.1 | Yes |
|  | Spermidine | 0.08 | 0.1 | 8 | 0.17 | 0.1 | Yes |
|  | Spermine | 0.08 | 0.25 | 20 | 2.08 | 0.17 | Yes |
|  | Taurine | 0.8 | 10 | 80 | 0 | 0.04 | Yes |
|  | cis-4-Hydroxyproline | 0.1 | 1 | 80 | 99.83 | NA | No |
|  | trans-4-Hydroxyproline | 0.1 | 0.25 | 20 | 0 | 0.04 | Yes |
| Short-chain Acylcarntines | C0(Carnitine) | [1.32-1.52] | 5 | 120 | 0 | 0.05 | Yes |
|  | C2(Acetylcarnitine) | [0.059-0.076] | 0.4 | 35 | 0 | 0.04 | Yes |
|  | C3(Propionylcarnitine) | [0.004-0.012] | 0.4 | 15 | 0 | 0.05 | Yes |
|  | C3-OH(Hydroxypropionylcarnitine) | [0.007-0.01] | NA | NA | 0.35 | 0.1 | Yes |
|  | C3:1(Propenoylcarnitine) | [0.003-0.012] | NA | NA | 0.17 | 0.1 | Yes |
|  | C3-DC (C4-OH)(Hydroxybutyrylcarnitine) | [0.006-0.022] | NA | NA | 0.17 | 0.08 | Yes |
|  | C4(Butyrylcarnitine) | [0.031-0.044] | 0.4 | 12 | 0 | 0.04 | Yes |
|  | C4:1(Butenylcarnitine) | [0.006-0.018] | NA | NA | 1.04 | 0.11 | Yes |
|  | C5(Valerylcarnitine) | [0.007-0.02] | 0.4 | 12 | 0 | 0.06 | Yes |
|  | C5-M-DC(Methylglutarylcarnitine) | [0.036-0.042] | NA | NA | 21.8 | 0.09 | Yes |
|  | C5:1(Tiglylcarnitine) | [0.009-0.026] | NA | NA | 0.52 | 0.08 | Yes |
|  | C5:1-DC(Glutaconylcarnitine) | [0.005-0.017] | NA | NA | 0.35 | 0.17 | Yes |
|  | C5-OH (C3-DC-M)(Hydroxyvalerylcarnitine (Methylmalonylcarnitine)) | [0.006-0.022] | NA | NA | 0 | 0.08 | Yes |
|  | C5-DC (C6-OH)(Glutarylcarnitine* (Hydroxyhexanoylcarnitine)) | [0.003-0.018] | NA | NA | 0.17 | 0.14 | Yes |
| Medium-chain Acylcarntines | C6 (C4:1-DC)(Hexanoylcarnitine (Fumarylcarnitine)) | [0.039-0.052] | NA | NA | 0.35 | 0.06 | Yes |
|  | C6:1(Hexenoylcarnitine) | [0.007-0.022] | NA | NA | 0.52 | 0.1 | Yes |
|  | C7-DC(Pimelylcarnitine) | [0.007-0.021] | NA | NA | 1.38 | 0.13 | Yes |
|  | C8(Octanoylcarnitine) | [0.115-0.157] | 0.2 | 8 | 21.8 | 0.05 | Yes |
|  | C9(Nonaylcarnitine) | [0.005-0.009] | NA | NA | 0 | 0.08 | Yes |
|  | C10(Decanoylcarnitine) | [0.051-0.064] | 0.3 | 6 | 1.21 | 0.04 | Yes |
|  | C10:1(Decenoylcarnitine) | [0.832-1.17] | NA | NA | 100 | NA | No |
|  | C10:2(Decadienylcarnitine) | [0.013-0.049] | NA | NA | 9.34 | 0.06 | Yes |
|  | C12(Dodecanoylcarnitine) | [0.054-0.063] | 0.4 | 12 | 22.49 | 0.05 | Yes |
|  | C12-DC(Dodecanedioylcarnitine) | [0.11-0.129] | NA | NA | 100 | NA | No |
|  | C12:1(Dodecenoylcarnitine) | [0.584-0.873] | NA | NA | 100 | NA | No |
| long-chain Acylcarntines | C14(Tetradecanoylcarnitine) | [0.028-0.038] | 0.4 | 6 | 45.16 | 0.04 | No |
|  | C14:1(Tetradecenoylcarnitine) | [0.003-0.005] | NA | NA | 0 | 0.15 | Yes |
|  | C14:1-OH(Hydroxytetradecenoylcarnitine) | [0.008-0.01] | NA | NA | 1.04 | 0.09 | Yes |
|  | C14:2(Tetradecadienylcarnitine) | [0.005-0.024] | NA | NA | 4.5 | 0.05 | Yes |
|  | C14:2-OH(Hydroxytetradecadienylcarnitine) | [0.003-0.007] | NA | NA | 2.42 | 0.1 | Yes |
|  | C16(Hexadecanoylcarnitine) | [0.008-0.01] | 0.4 | 12 | 0 | 0.05 | Yes |
|  | C16-OH(Hydroxyhexadecanoylcarnitine) | [0.002-0.007] | NA | NA | 8.48 | 0.1 | Yes |
|  | C16:1(Hexadecenoylcarnitine) | [0.008-0.011] | NA | NA | 0.35 | 0.06 | Yes |
|  | C16:1-OH(Hydroxyhexadecenoylcarnitine) | [0.009-0.014] | NA | NA | 7.79 | 0.07 | Yes |
|  | C16:2(Hexadecadienylcarnitine) | [0.007-0.015] | NA | NA | 23.7 | 0.06 | Yes |
|  | C16:2-OH(Hydroxyhexadecadienylcarnitine) | [0.006-0.013] | NA | NA | 11.76 | 0.06 | Yes |
|  | C18(Octadecanoylcarnitine) | [0.008-0.012] | 0.4 | 6 | 0 | 0.05 | Yes |
|  | C18:1(Octadecenoylcarnitine) | [0.014-0.033] | NA | NA | 0 | 0.04 | Yes |
|  | C18:1-OH(Hydroxyoctadecenoylcarnitine) | [0.006-0.009] | NA | NA | 5.88 | 0.1 | Yes |
|  | C18:2(Octadecadienylcarnitine) | [0.004-0.015] | NA | NA | 0 | 0.04 | Yes |
| Sphingolipids | SM (OH) C14:1 | [0.054-0.114] | NA | NA | 0 | 0.07 | Yes |
|  | SM C16:0 | [0.018-0.046] | NA | NA | 0 | 0.07 | Yes |
|  | SM (OH) C16:1 | [0-0.005] | NA | NA | 0 | 0.07 | Yes |
|  | SM C16:1 | [0.017-0.033] | NA | NA | 0 | 0.07 | Yes |
|  | SM C18:0 | [0.008-0.038] | NA | NA | 0 | 0.07 | Yes |
|  | SM C18:1 | [0-0.005] | NA | NA | 0 | 0.07 | Yes |
|  | SM C20:2 | [0.002-0.005] | NA | NA | 0 | 0.32 | Yes |
|  | SM (OH) C22:1 | [0.011-0.035] | NA | NA | 0 | 0.09 | Yes |
|  | SM (OH) C22:2 | [0.005-0.017] | NA | NA | 0 | 0.07 | Yes |
|  | SM C24:0 | [0.014-0.031] | NA | NA | 0 | 0.1 | Yes |
|  | SM (OH) C24:1 | [0.001-0.004] | NA | NA | 0 | 0.1 | Yes |
|  | SM C24:1 | [0.005-0.033] | NA | NA | 0 | 0.08 | Yes |
|  | SM C26:0 | [0.002-0.032] | NA | NA | 0 | 0.12 | Yes |
|  | SM C26:1 | [0.001-0.006] | NA | NA | 0 | 0.09 | Yes |
| Glycerophospholipids (PC aa) | PC aa C24:0 | [0.02-0.03] | NA | NA | 0.87 | 0.2 | Yes |
|  | PC aa C26:0 | [0.706-0.737] | NA | NA | 99.83 | NA | No |
|  | PC aa C28:1 | [0.007-0.023] | NA | NA | 0 | 0.04 | Yes |
|  | PC aa C30:0 | [0.129-0.137] | NA | NA | 0 | 0.04 | Yes |
|  | PC aa C32:0 | [0.007-0.017] | NA | NA | 0 | 0.04 | Yes |
|  | PC aa C32:1 | [0.003-0.006] | NA | NA | 0 | 0.05 | Yes |
|  | PC aa C32:3 | [0.004-0.007] | NA | NA | 0 | 0.05 | Yes |
|  | PC aa C34:1 | [0.006-0.112] | NA | NA | 0 | 0.09 | Yes |
|  | PC aa C34:2 | [0.006-0.275] | NA | NA | 0 | 0.09 | Yes |
|  | PC aa C34:3 | [0.001-0.015] | NA | NA | 0 | 0.05 | Yes |
|  | PC aa C34:4 | [0.002-0.004] | NA | NA | 0 | 0.05 | Yes |
|  | PC aa C36:0 | [0.527-0.565] | NA | NA | 75.78 | NA | No |
|  | PC aa C36:1 | [0.007-0.018] | NA | NA | 0 | 0.04 | Yes |
|  | PC aa C36:2 | [0.021-0.079] | NA | NA | 0 | 0.09 | Yes |
|  | PC aa C36:3 | [0.003-0.248] | NA | NA | 0 | 0.09 | Yes |
|  | PC aa C36:4 | [0.004-0.395] | NA | NA | 0 | 0.09 | Yes |
|  | PC aa C36:5 | [0.003-0.006] | NA | NA | 0 | 0.05 | Yes |
|  | PC aa C36:6 | [0-0.015] | NA | NA | 0 | 0.05 | Yes |
|  | PC aa C38:0 | [0.017-0.022] | NA | NA | 0 | 0.04 | Yes |
|  | PC aa C38:3 | [0.009-0.015] | NA | NA | 0 | 0.04 | Yes |
|  | PC aa C38:4 | [0.007-0.028] | NA | NA | 0 | 0.07 | Yes |
|  | PC aa C38:5 | [0.004-0.014] | NA | NA | 0 | 0.05 | Yes |
|  | PC aa C38:6 | [0-0.007] | NA | NA | 0 | 0.04 | Yes |
|  | PC aa C40:1 | [0.296-0.324] | NA | NA | 51.73 | 0.02 | No |
|  | PC aa C40:2 | [0.005-0.009] | NA | NA | 0 | 0.06 | Yes |
|  | PC aa C40:3 | [0.002-0.012] | NA | NA | 0 | 0.05 | Yes |
|  | PC aa C40:4 | [0.004-0.007] | NA | NA | 0 | 0.04 | Yes |
|  | PC aa C40:5 | 0.04 | NA | NA | 0 | 0.05 | Yes |
|  | PC aa C40:6 | [0.265-0.29] | NA | NA | 0 | 0.04 | Yes |
|  | PC aa C42:0 | [0.038-0.046] | NA | NA | 0 | 0.04 | Yes |
|  | PC aa C42:1 | [0.004-0.008] | NA | NA | 0 | 0.04 | Yes |
|  | PC aa C42:2 | [0.059-0.071] | NA | NA | 0.17 | 0.04 | Yes |
|  | PC aa C42:4 | [0.007-0.014] | NA | NA | 0 | 0.05 | Yes |
|  | PC aa C42:5 | [0.002-0.007] | NA | NA | 0 | 0.04 | Yes |
|  | PC aa C42:6 | [0.093-0.133] | NA | NA | 0.17 | 0.04 | Yes |
| Glycerophospholipids (PC ae) | PC ae C30:0 | [0.074-0.089] | NA | NA | 0.17 | 0.04 | Yes |
|  | PC ae C30:1 | 0.02 | NA | NA | 41.52 | 0.3 | No |
|  | PC ae C30:2 | [0.003-0.029] | NA | NA | 0 | 0.05 | Yes |
|  | PC ae C32:1 | [0.001-0.003] | NA | NA | 0 | 0.05 | Yes |
|  | PC ae C32:2 | [0.007-0.012] | NA | NA | 0 | 0.05 | Yes |
|  | PC ae C34:0 | [0.003-0.007] | NA | NA | 0 | 0.05 | Yes |
|  | PC ae C34:1 | [0.006-0.022] | NA | NA | 0 | 0.04 | Yes |
|  | PC ae C34:2 | [0.002-0.005] | NA | NA | 0 | 0.05 | Yes |
|  | PC ae C34:3 | [0.003-0.005] | NA | NA | 0 | 0.05 | Yes |
|  | PC ae C36:0 | [0.129-0.142] | NA | NA | 0.17 | 0.05 | Yes |
|  | PC ae C36:1 | [0.07-0.106] | NA | NA | 0 | 0.05 | Yes |
|  | PC ae C36:2 | [0.031-0.037] | NA | NA | 0 | 0.05 | Yes |
|  | PC ae C36:3 | [0.001-0.007] | NA | NA | 0 | 0.05 | Yes |
|  | PC ae C36:4 | [0.027-0.067] | NA | NA | 0 | 0.05 | Yes |
|  | PC ae C36:5 | [0.003-0.006] | NA | NA | 0 | 0.05 | Yes |
|  | PC ae C38:0 | [0.056-0.061] | NA | NA | 0 | 0.04 | Yes |
|  | PC ae C38:1 | [0.011-0.029] | NA | NA | 61.59 | 0.56 | No |
|  | PC ae C38:2 | [0.016-0.033] | NA | NA | 0 | 0.08 | Yes |
|  | PC ae C38:3 | [0.013-0.025] | NA | NA | 0 | 0.04 | Yes |
|  | PC ae C38:4 | [0.005-0.019] | NA | NA | 0 | 0.05 | Yes |
|  | PC ae C38:5 | [0.005-0.009] | NA | NA | 0 | 0.04 | Yes |
|  | PC ae C38:6 | [0.001-0.03] | NA | NA | 0 | 0.04 | Yes |
|  | PC ae C40:1 | [0.006-0.009] | NA | NA | 0 | 0.06 | Yes |
|  | PC ae C40:2 | [0.001-0.005] | NA | NA | 0 | 0.04 | Yes |
|  | PC ae C40:3 | [0.001-0.015] | NA | NA | 0 | 0.04 | Yes |
|  | PC ae C40:4 | [0.048-0.059] | NA | NA | 0 | 0.04 | Yes |
|  | PC ae C40:5 | [0.001-0.003] | NA | NA | 0 | 0.04 | Yes |
|  | PC ae C40:6 | [0.002-0.003] | NA | NA | 0 | 0.04 | Yes |
|  | PC ae C42:0 | [0.666-0.727] | NA | NA | 98.44 | NA | No |
|  | PC ae C42:1 | [0.103-0.136] | NA | NA | 0.17 | 0.04 | Yes |
|  | PC ae C42:2 | [0.007-0.012] | NA | NA | 0 | 0.03 | Yes |
|  | PC ae C42:3 | [0.002-0.005] | NA | NA | 0 | 0.04 | Yes |
|  | PC ae C42:4 | 0.3 | NA | NA | 0.17 | 0.04 | Yes |
|  | PC ae C42:5 | [0.64-0.668] | NA | NA | 0.17 | 0.04 | Yes |
|  | PC ae C44:3 | [0.029-0.038] | NA | NA | 0.17 | 0.06 | Yes |
|  | PC ae C44:4 | [0.082-0.094] | NA | NA | 0.17 | 0.05 | Yes |
|  | PC ae C44:5 | [0.047-0.055] | NA | NA | 0 | 0.04 | Yes |
|  | PC ae C44:6 | [0.019-0.025] | NA | NA | 0 | 0.04 | Yes |
| lysoPhosphatidylcholines | lysoPC a C14:0 | [10.6-11.3] | NA | NA | 100 | NA | No |
|  | lysoPC a C16:0 | [0.044-0.07] | NA | NA | 0 | 0.04 | Yes |
|  | lysoPC a C16:1 | [0.037-0.048] | NA | NA | 0 | 0.04 | Yes |
|  | lysoPC a C17:0 | [0.006-0.016] | NA | NA | 0 | 0.04 | Yes |
|  | lysoPC a C18:0 | [0.16-0.215] | NA | NA | 0 | 0.04 | Yes |
|  | lysoPC a C18:1 | [0.016-0.055] | NA | NA | 0 | 0.04 | Yes |
|  | lysoPC a C18:2 | [0.018-0.103] | NA | NA | 0 | 0.05 | Yes |
|  | lysoPC a C20:3 | [0.288-0.352] | NA | NA | 0 | 0.05 | Yes |
|  | lysoPC a C20:4 | [0.004-0.063] | NA | NA | 0 | 0.05 | Yes |
|  | lysoPC a C24:0 | [0.011-0.02] | NA | NA | 0 | 0.09 | Yes |
|  | lysoPC a C26:0 | [0.011-0.015] | NA | NA | 0 | 0.21 | Yes |
|  | lysoPC a C26:1 | [0.005-0.019] | NA | NA | 0 | 0.18 | Yes |
|  | lysoPC a C28:0 | [0.072-0.099] | NA | NA | 0.17 | 0.13 | Yes |
|  | lysoPC a C28:1 | [0.003-0.011] | NA | NA | 0 | 0.09 | Yes |

*Abbreviations*: SM: sphingomyelin, PC: Phosphatidylcholine, LysoPC: Lyso-Phosphatidylcholine.
