# Supplementary material for: Alterations in acylcarnitines, amines, and lipids inform about the mechanism of action of citalopram/escitalopram in major depression
Source: Transl Psychiatry. 2021 Mar 2;11:153. doi: 10.1038/s41398-020-01097-6 (PMC7925685; doi:10.1038/s41398-020-01097-6)
Supplement: Supplementary file 3 — Supplementary Table 2 [file 41398_2020_1097_MOESM3_ESM.docx]

**Supplementary Table 2. Significant Changes in Metabolite Levels after 8 weeks of SSRI Treatment Stratified by Sex, Age and Drug**

| *Class* | Metabolite | Estimated Log2 Change (SE) | p-value(q-value) |
| --- | --- | --- | --- |
| **Short-chain Acylcarnitines** | **C5-OH (C3-DC-M)** | All Participants:-0.24(0.06) Females:-0.23(0.08) Males:-0.25(0.11) [19.3,31.5] Years old:-0.18(0.12) (31.5,47.1] Years old:-0.20(0.11) (47.1,84.7] Years old:-0.35(0.10) Escitalopram:-0.17(0.07) Citalopram:-0.41(0.13) | All Participants:2.24E-04(3.04E-03) Females:2.74E-03(2.58E-02) Males:2.68E-02(1.12E-01) [19.3,31.5] Years old:1.30E-01(4.94E-01) (31.5,47.1] Years old:8.55E-02(1.70E-01) (47.1,84.7] Years old:1.39E-03(3.78E-02) Escitalopram:1.98E-02(9.78E-02) Citalopram:2.57E-03(3.81E-02) |
|  | **C3** | All Participants:0.16(0.04) Females:0.09(0.06) Males:0.27(0.06) [19.3,31.5] Years old:0.15(0.07) (31.5,47.1] Years old:0.28(0.08) (47.1,84.7] Years old:0.04(0.08) Escitalopram:0.14(0.05) Citalopram:0.21(0.09) | All Participants:3.24E-04(3.61E-03) Females:1.03E-01(3.11E-01) Males:4.64E-05(3.78E-03) [19.3,31.5] Years old:2.56E-02(2.01E-01) (31.5,47.1] Years old:4.87E-04(1.95E-02) (47.1,84.7] Years old:5.98E-01(8.42E-01) Escitalopram:5.82E-03(4.34E-02) Citalopram:2.40E-02(9.33E-02) |
|  | **C5** | All Participants:0.13(0.04) Females:0.06(0.06) Males:0.27(0.07) [19.3,31.5] Years old:0.14(0.07) (31.5,47.1] Years old:0.30(0.08) (47.1,84.7] Years old:-0.04(0.08) Escitalopram:0.12(0.05) Citalopram:0.16(0.08) | All Participants:2.47E-03(1.34E-02) Females:2.86E-01(6.06E-01) Males:2.10E-04(7.34E-03) [19.3,31.5] Years old:5.23E-02(2.94E-01) (31.5,47.1] Years old:1.72E-04(1.36E-02) (47.1,84.7] Years old:5.85E-01(8.42E-01) Escitalopram:1.64E-02(8.96E-02) Citalopram:6.79E-02(1.68E-01) |
|  | **C2** | All Participants:-0.16(0.06) Females:-0.23(0.07) Males:-0.04(0.08) [19.3,31.5] Years old:-0.25(0.09) (31.5,47.1] Years old:-0.09(0.09) (47.1,84.7] Years old:-0.15(0.10) Escitalopram:-0.18(0.07) Citalopram:-0.12(0.10) | All Participants:3.47E-03(1.71E-02) Females:2.17E-03(2.21E-02) Males:5.86E-01(6.59E-01) [19.3,31.5] Years old:9.19E-03(9.99E-02) (31.5,47.1] Years old:3.10E-01(4.21E-01) (47.1,84.7] Years old:1.61E-01(5.72E-01) Escitalopram:6.44E-03(4.56E-02) Citalopram:2.48E-01(3.64E-01) |
|  | **C4** | All Participants:0.09(0.04) Females:0.03(0.05) Males:0.21(0.05) [19.3,31.5] Years old:0.06(0.07) (31.5,47.1] Years old:0.18(0.06) (47.1,84.7] Years old:0.05(0.06) Escitalopram:0.06(0.04) Citalopram:0.18(0.07) | All Participants:9.16E-03(3.39E-02) Females:5.06E-01(7.48E-01) Males:1.70E-04(7.34E-03) [19.3,31.5] Years old:4.05E-01(7.84E-01) (31.5,47.1] Years old:2.93E-03(5.30E-02) (47.1,84.7] Years old:4.25E-01(7.93E-01) Escitalopram:1.47E-01(3.47E-01) Citalopram:1.73E-02(8.81E-02) |
|  | C0 | All Participants:0.06(0.03) Females:0.03(0.04) Males:0.12(0.04) [19.3,31.5] Years old:0.07(0.05) (31.5,47.1] Years old:0.08(0.05) (47.1,84.7] Years old:0.03(0.04) Escitalopram:0.03(0.03) Citalopram:0.13(0.05) | All Participants:2.75E-02(8.00E-02) Females:4.11E-01(6.79E-01) Males:4.57E-03(4.66E-02) [19.3,31.5] Years old:1.47E-01(5.09E-01) (31.5,47.1] Years old:1.19E-01(2.04E-01) (47.1,84.7] Years old:4.94E-01(8.21E-01) Escitalopram:3.41E-01(5.30E-01) Citalopram:1.20E-02(8.16E-02) |
|  | C5-M-DC | All Participants:0.14(0.06) Females:0.03(0.08) Males:0.32(0.11) [19.3,31.5] Years old:0.00(0.10) (31.5,47.1] Years old:0.34(0.12) (47.1,84.7] Years old:0.07(0.10) Escitalopram:0.08(0.07) Citalopram:0.27(0.11) | All Participants:3.09E-02(8.85E-02) Females:6.61E-01(8.03E-01) Males:4.10E-03(4.66E-02) [19.3,31.5] Years old:9.79E-01(9.91E-01) (31.5,47.1] Years old:5.59E-03(6.08E-02) (47.1,84.7] Years old:5.24E-01(8.27E-01) Escitalopram:2.81E-01(4.98E-01) Citalopram:1.92E-02(9.19E-02) |
|  | C5:1 | All Participants:0.05(0.03) Females:0.00(0.04) Males:0.14(0.06) [19.3,31.5] Years old:0.02(0.06) (31.5,47.1] Years old:0.15(0.06) (47.1,84.7] Years old:-0.01(0.05) Escitalopram:0.02(0.04) Citalopram:0.13(0.06) | All Participants:1.28E-01(2.23E-01) Females:9.35E-01(9.64E-01) Males:3.10E-02(1.20E-01) [19.3,31.5] Years old:6.90E-01(8.57E-01) (31.5,47.1] Years old:2.22E-02(9.06E-02) (47.1,84.7] Years old:7.75E-01(9.03E-01) Escitalopram:6.35E-01(7.97E-01) Citalopram:2.80E-02(1.01E-01) |
|  | C3-OH | All Participants:0.05(0.03) Females:0.02(0.04) Males:0.09(0.06) [19.3,31.5] Years old:0.03(0.05) (31.5,47.1] Years old:0.12(0.06) (47.1,84.7] Years old:-0.01(0.05) Escitalopram:0.02(0.04) Citalopram:0.12(0.06) | All Participants:1.34E-01(2.30E-01) Females:5.11E-01(7.48E-01) Males:1.34E-01(2.45E-01) [19.3,31.5] Years old:5.77E-01(8.40E-01) (31.5,47.1] Years old:5.92E-02(1.46E-01) (47.1,84.7] Years old:8.83E-01(9.52E-01) Escitalopram:6.33E-01(7.97E-01) Citalopram:3.80E-02(1.26E-01) |
|  | C3:1 | All Participants:0.03(0.03) Females:0.00(0.04) Males:0.08(0.06) [19.3,31.5] Years old:-0.02(0.06) (31.5,47.1] Years old:0.12(0.07) (47.1,84.7] Years old:-0.01(0.05) Escitalopram:0.00(0.04) Citalopram:0.10(0.06) | All Participants:3.73E-01(4.94E-01) Females:9.27E-01(9.64E-01) Males:2.25E-01(3.37E-01) [19.3,31.5] Years old:7.70E-01(8.78E-01) (31.5,47.1] Years old:9.05E-02(1.74E-01) (47.1,84.7] Years old:8.56E-01(9.39E-01) Escitalopram:9.77E-01(9.83E-01) Citalopram:8.86E-02(2.01E-01) |
|  | C4:1 | All Participants:0.03(0.04) Females:-0.01(0.05) Males:0.11(0.07) [19.3,31.5] Years old:0.03(0.07) (31.5,47.1] Years old:0.09(0.07) (47.1,84.7] Years old:-0.02(0.06) Escitalopram:-0.00(0.04) Citalopram:0.11(0.08) | All Participants:4.25E-01(5.37E-01) Females:7.77E-01(8.94E-01) Males:1.27E-01(2.41E-01) [19.3,31.5] Years old:7.28E-01(8.62E-01) (31.5,47.1] Years old:2.02E-01(3.02E-01) (47.1,84.7] Years old:7.24E-01(8.95E-01) Escitalopram:9.49E-01(9.79E-01) Citalopram:1.51E-01(2.64E-01) |
|  | C3-DC (C4-OH) | All Participants:-0.03(0.05) Females:-0.07(0.06) Males:0.04(0.07) [19.3,31.5] Years old:-0.04(0.08) (31.5,47.1] Years old:0.01(0.09) (47.1,84.7] Years old:-0.07(0.07) Escitalopram:-0.06(0.06) Citalopram:0.04(0.07) | All Participants:4.94E-01(6.01E-01) Females:2.57E-01(5.58E-01) Males:6.26E-01(6.99E-01) [19.3,31.5] Years old:6.18E-01(8.52E-01) (31.5,47.1] Years old:8.87E-01(9.09E-01) (47.1,84.7] Years old:3.54E-01(7.37E-01) Escitalopram:2.91E-01(4.98E-01) Citalopram:5.43E-01(6.06E-01) |
|  | C5-DC (C6-OH) | All Participants:0.01(0.03) Females:-0.05(0.04) Males:0.10(0.05) [19.3,31.5] Years old:-0.05(0.06) (31.5,47.1] Years old:0.11(0.06) (47.1,84.7] Years old:-0.04(0.05) Escitalopram:-0.04(0.04) Citalopram:0.11(0.06) | All Participants:8.42E-01(9.03E-01) Females:2.56E-01(5.58E-01) Males:5.68E-02(1.54E-01) [19.3,31.5] Years old:3.82E-01(7.82E-01) (31.5,47.1] Years old:7.97E-02(1.68E-01) (47.1,84.7] Years old:4.34E-01(7.95E-01) Escitalopram:3.84E-01(5.80E-01) Citalopram:6.45E-02(1.68E-01) |
|  | C5:1-DC | All Participants:0.00(0.04) Females:-0.03(0.04) Males:0.07(0.07) [19.3,31.5] Years old:-0.02(0.06) (31.5,47.1] Years old:0.13(0.07) (47.1,84.7] Years old:-0.09(0.06) Escitalopram:-0.02(0.05) Citalopram:0.06(0.06) | All Participants:8.95E-01(9.33E-01) Females:4.94E-01(7.43E-01) Males:3.34E-01(4.62E-01) [19.3,31.5] Years old:7.35E-01(8.62E-01) (31.5,47.1] Years old:8.39E-02(1.70E-01) (47.1,84.7] Years old:1.17E-01(4.79E-01) Escitalopram:6.67E-01(8.30E-01) Citalopram:2.90E-01(4.02E-01) |
| **Medium-chain Acylcarnitines** | **C10** | All Participants:-0.33(0.08) Females:-0.40(0.10) Males:-0.21(0.11) [19.3,31.5] Years old:-0.49(0.13) (31.5,47.1] Years old:-0.14(0.13) (47.1,84.7] Years old:-0.35(0.14) Escitalopram:-0.33(0.09) Citalopram:-0.31(0.15) | All Participants:2.91E-05(5.93E-04) Females:1.75E-04(3.56E-03) Males:6.63E-02(1.64E-01) [19.3,31.5] Years old:3.68E-04(1.30E-02) (31.5,47.1] Years old:2.85E-01(4.03E-01) (47.1,84.7] Years old:1.30E-02(1.43E-01) Escitalopram:2.41E-04(4.92E-03) Citalopram:4.50E-02(1.37E-01) |
|  | **C12** | All Participants:-0.32(0.08) Females:-0.40(0.10) Males:-0.18(0.13) [19.3,31.5] Years old:-0.48(0.14) (31.5,47.1] Years old:-0.11(0.13) (47.1,84.7] Years old:-0.36(0.15) Escitalopram:-0.37(0.09) Citalopram:-0.20(0.16) | All Participants:8.86E-05(1.55E-03) Females:1.26E-04(2.94E-03) Males:1.71E-01(2.88E-01) [19.3,31.5] Years old:6.39E-04(1.30E-02) (31.5,47.1] Years old:3.97E-01(4.94E-01) (47.1,84.7] Years old:1.67E-02(1.60E-01) Escitalopram:9.50E-05(2.58E-03) Citalopram:2.12E-01(3.23E-01) |
|  | **C8** | All Participants:-0.31(0.08) Females:-0.41(0.10) Males:-0.12(0.12) [19.3,31.5] Years old:-0.49(0.14) (31.5,47.1] Years old:-0.12(0.14) (47.1,84.7] Years old:-0.31(0.13) Escitalopram:-0.32(0.09) Citalopram:-0.29(0.16) | All Participants:1.10E-04(1.64E-03) Females:8.22E-05(2.23E-03) Males:3.17E-01(4.49E-01) [19.3,31.5] Years old:5.58E-04(1.30E-02) (31.5,47.1] Years old:4.03E-01(4.95E-01) (47.1,84.7] Years old:1.97E-02(1.68E-01) Escitalopram:4.27E-04(7.73E-03) Citalopram:8.67E-02(2.01E-01) |
|  | **C7-DC** | All Participants:-0.17(0.05) Females:-0.18(0.06) Males:-0.17(0.09) [19.3,31.5] Years old:-0.24(0.08) (31.5,47.1] Years old:-0.04(0.08) (47.1,84.7] Years old:-0.24(0.10) Escitalopram:-0.19(0.06) Citalopram:-0.14(0.09) | All Participants:5.08E-04(4.14E-03) Females:3.85E-03(3.30E-02) Males:5.44E-02(1.54E-01) [19.3,31.5] Years old:4.87E-03(6.11E-02) (31.5,47.1] Years old:6.47E-01(7.06E-01) (47.1,84.7] Years old:1.19E-02(1.43E-01) Escitalopram:1.85E-03(2.32E-02) Citalopram:1.22E-01(2.39E-01) |
|  | C6 (C4:1-DC) | All Participants:-0.07(0.04) Females:-0.12(0.05) Males:0.02(0.06) [19.3,31.5] Years old:-0.10(0.07) (31.5,47.1] Years old:0.03(0.06) (47.1,84.7] Years old:-0.14(0.07) Escitalopram:-0.06(0.04) Citalopram:-0.09(0.07) | All Participants:5.71E-02(1.35E-01) Females:1.19E-02(7.77E-02) Males:7.42E-01(7.90E-01) [19.3,31.5] Years old:1.43E-01(5.09E-01) (31.5,47.1] Years old:6.56E-01(7.08E-01) (47.1,84.7] Years old:3.29E-02(2.06E-01) Escitalopram:1.55E-01(3.50E-01) Citalopram:1.98E-01(3.20E-01) |
|  | C10:2 | All Participants:0.04(0.04) Females:0.04(0.05) Males:0.02(0.07) [19.3,31.5] Years old:0.06(0.07) (31.5,47.1] Years old:0.08(0.08) (47.1,84.7] Years old:-0.03(0.07) Escitalopram:0.02(0.05) Citalopram:0.08(0.08) | All Participants:3.92E-01(5.11E-01) Females:4.04E-01(6.79E-01) Males:7.54E-01(7.93E-01) [19.3,31.5] Years old:4.11E-01(7.84E-01) (31.5,47.1] Years old:2.98E-01(4.09E-01) (47.1,84.7] Years old:7.09E-01(8.92E-01) Escitalopram:7.42E-01(8.52E-01) Citalopram:2.91E-01(4.02E-01) |
|  | C9 | All Participants:0.03(0.03) Females:0.00(0.04) Males:0.07(0.06) [19.3,31.5] Years old:0.01(0.06) (31.5,47.1] Years old:0.14(0.06) (47.1,84.7] Years old:-0.08(0.05) Escitalopram:0.00(0.04) Citalopram:0.09(0.05) | All Participants:4.36E-01(5.47E-01) Females:9.84E-01(9.86E-01) Males:2.32E-01(3.41E-01) [19.3,31.5] Years old:7.96E-01(8.87E-01) (31.5,47.1] Years old:2.02E-02(8.82E-02) (47.1,84.7] Years old:1.51E-01(5.57E-01) Escitalopram:9.99E-01(9.99E-01) Citalopram:1.13E-01(2.31E-01) |
|  | C6:1 | All Participants:0.02(0.03) Females:-0.00(0.04) Males:0.05(0.06) [19.3,31.5] Years old:-0.01(0.05) (31.5,47.1] Years old:0.10(0.06) (47.1,84.7] Years old:-0.04(0.05) Escitalopram:-0.01(0.04) Citalopram:0.09(0.06) | All Participants:6.22E-01(7.04E-01) Females:9.39E-01(9.64E-01) Males:4.17E-01(5.19E-01) [19.3,31.5] Years old:7.84E-01(8.87E-01) (31.5,47.1] Years old:1.15E-01(1.99E-01) (47.1,84.7] Years old:4.83E-01(8.20E-01) Escitalopram:7.17E-01(8.44E-01) Citalopram:1.29E-01(2.44E-01) |
| **long-chain Acylcarnitnes** | **C18:1** | All Participants:-0.38(0.06) Females:-0.36(0.08) Males:-0.43(0.11) [19.3,31.5] Years old:-0.40(0.10) (31.5,47.1] Years old:-0.36(0.12) (47.1,84.7] Years old:-0.39(0.11) Escitalopram:-0.39(0.08) Citalopram:-0.37(0.11) | All Participants:6.67E-09(4.98E-07) Females:6.05E-06(3.29E-04) Males:2.25E-04(7.34E-03) [19.3,31.5] Years old:2.21E-04(1.20E-02) (31.5,47.1] Years old:3.58E-03(5.30E-02) (47.1,84.7] Years old:6.93E-04(3.35E-02) Escitalopram:1.46E-06(9.04E-05) Citalopram:1.22E-03(2.83E-02) |
|  | **C16:1** | All Participants:-0.31(0.05) Females:-0.32(0.07) Males:-0.30(0.09) [19.3,31.5] Years old:-0.38(0.09) (31.5,47.1] Years old:-0.24(0.09) (47.1,84.7] Years old:-0.32(0.09) Escitalopram:-0.32(0.06) Citalopram:-0.31(0.09) | All Participants:9.17E-09(4.98E-07) Females:3.74E-06(3.05E-04) Males:8.36E-04(1.51E-02) [19.3,31.5] Years old:7.51E-05(6.12E-03) (31.5,47.1] Years old:8.64E-03(7.32E-02) (47.1,84.7] Years old:1.03E-03(3.35E-02) Escitalopram:1.66E-06(9.04E-05) Citalopram:1.71E-03(3.10E-02) |
|  | **C18:2** | All Participants:-0.38(0.07) Females:-0.35(0.08) Males:-0.44(0.12) [19.3,31.5] Years old:-0.35(0.11) (31.5,47.1] Years old:-0.38(0.13) (47.1,84.7] Years old:-0.42(0.11) Escitalopram:-0.38(0.08) Citalopram:-0.38(0.11) | All Participants:2.51E-08(1.02E-06) Females:1.94E-05(6.31E-04) Males:3.40E-04(9.25E-03) [19.3,31.5] Years old:1.74E-03(3.10E-02) (31.5,47.1] Years old:5.33E-03(6.08E-02) (47.1,84.7] Years old:1.90E-04(1.55E-02) Escitalopram:4.33E-06(1.76E-04) Citalopram:1.48E-03(3.02E-02) |
|  | **C14:2** | All Participants:-0.36(0.08) Females:-0.46(0.10) Males:-0.20(0.12) [19.3,31.5] Years old:-0.41(0.13) (31.5,47.1] Years old:-0.20(0.14) (47.1,84.7] Years old:-0.48(0.14) Escitalopram:-0.37(0.09) Citalopram:-0.34(0.15) | All Participants:4.58E-06(1.23E-04) Females:1.33E-05(5.40E-04) Males:9.38E-02(2.12E-01) [19.3,31.5] Years old:1.90E-03(3.10E-02) (31.5,47.1] Years old:1.47E-01(2.34E-01) (47.1,84.7] Years old:9.07E-04(3.35E-02) Escitalopram:7.53E-05(2.45E-03) Citalopram:2.14E-02(9.33E-02) |
|  | **C16** | All Participants:-0.21(0.05) Females:-0.19(0.05) Males:-0.24(0.09) [19.3,31.5] Years old:-0.27(0.07) (31.5,47.1] Years old:-0.15(0.08) (47.1,84.7] Years old:-0.21(0.08) Escitalopram:-0.23(0.06) Citalopram:-0.18(0.07) | All Participants:5.28E-06(1.23E-04) Females:2.59E-04(4.70E-03) Males:6.40E-03(4.97E-02) [19.3,31.5] Years old:4.69E-04(1.30E-02) (31.5,47.1] Years old:8.01E-02(1.68E-01) (47.1,84.7] Years old:8.07E-03(1.20E-01) Escitalopram:1.29E-04(3.00E-03) Citalopram:1.12E-02(7.90E-02) |
|  | **C14:1-OH** | All Participants:-0.14(0.04) Females:-0.15(0.05) Males:-0.11(0.07) [19.3,31.5] Years old:-0.21(0.07) (31.5,47.1] Years old:-0.02(0.06) (47.1,84.7] Years old:-0.18(0.06) Escitalopram:-0.16(0.05) Citalopram:-0.09(0.07) | All Participants:4.09E-04(3.79E-03) Females:1.21E-03(1.79E-02) Males:1.05E-01(2.26E-01) [19.3,31.5] Years old:3.61E-03(5.35E-02) (31.5,47.1] Years old:7.34E-01(7.76E-01) (47.1,84.7] Years old:6.77E-03(1.20E-01) Escitalopram:7.81E-04(1.16E-02) Citalopram:2.07E-01(3.20E-01) |
|  | **C16:1-OH** | All Participants:-0.13(0.05) Females:-0.16(0.05) Males:-0.08(0.09) [19.3,31.5] Years old:-0.21(0.09) (31.5,47.1] Years old:-0.01(0.07) (47.1,84.7] Years old:-0.16(0.07) Escitalopram:-0.16(0.06) Citalopram:-0.05(0.07) | All Participants:4.66E-03(2.00E-02) Females:1.82E-03(2.21E-02) Males:3.93E-01(5.07E-01) [19.3,31.5] Years old:2.73E-02(2.01E-01) (31.5,47.1] Years old:8.48E-01(8.75E-01) (47.1,84.7] Years old:1.77E-02(1.60E-01) Escitalopram:4.71E-03(4.34E-02) Citalopram:4.65E-01(5.61E-01) |
|  | **C16:2-OH** | All Participants:-0.13(0.05) Females:-0.14(0.05) Males:-0.12(0.09) [19.3,31.5] Years old:-0.25(0.09) (31.5,47.1] Years old:-0.06(0.08) (47.1,84.7] Years old:-0.08(0.07) Escitalopram:-0.12(0.05) Citalopram:-0.14(0.09) | All Participants:4.71E-03(2.00E-02) Females:8.77E-03(5.96E-02) Males:1.85E-01(3.02E-01) [19.3,31.5] Years old:4.27E-03(5.80E-02) (31.5,47.1] Years old:4.80E-01(5.70E-01) (47.1,84.7] Years old:2.62E-01(6.81E-01) Escitalopram:1.67E-02(8.96E-02) Citalopram:1.40E-01(2.56E-01) |
|  | **C18** | All Participants:-0.11(0.04) Females:-0.10(0.05) Males:-0.12(0.06) [19.3,31.5] Years old:-0.12(0.07) (31.5,47.1] Years old:-0.07(0.07) (47.1,84.7] Years old:-0.12(0.06) Escitalopram:-0.13(0.05) Citalopram:-0.04(0.06) | All Participants:4.96E-03(2.00E-02) Females:3.79E-02(1.67E-01) Males:5.81E-02(1.54E-01) [19.3,31.5] Years old:5.99E-02(3.14E-01) (31.5,47.1] Years old:2.82E-01(4.03E-01) (47.1,84.7] Years old:6.01E-02(2.88E-01) Escitalopram:5.02E-03(4.34E-02) Citalopram:4.75E-01(5.68E-01) |
|  | **C18:1-OH** | All Participants:-0.14(0.05) Females:-0.19(0.06) Males:-0.05(0.09) [19.3,31.5] Years old:-0.22(0.10) (31.5,47.1] Years old:-0.02(0.07) (47.1,84.7] Years old:-0.17(0.08) Escitalopram:-0.17(0.06) Citalopram:-0.06(0.08) | All Participants:4.97E-03(2.00E-02) Females:1.50E-03(2.04E-02) Males:5.86E-01(6.59E-01) [19.3,31.5] Years old:2.96E-02(2.01E-01) (31.5,47.1] Years old:8.06E-01(8.37E-01) (47.1,84.7] Years old:3.09E-02(2.06E-01) Escitalopram:4.22E-03(4.34E-02) Citalopram:5.00E-01(5.82E-01) |
|  | **C14:1** | All Participants:-0.10(0.04) Females:-0.14(0.04) Males:-0.04(0.06) [19.3,31.5] Years old:-0.14(0.06) (31.5,47.1] Years old:-0.01(0.06) (47.1,84.7] Years old:-0.16(0.07) Escitalopram:-0.13(0.04) Citalopram:-0.05(0.07) | All Participants:5.03E-03(2.00E-02) Females:2.05E-03(2.21E-02) Males:5.37E-01(6.25E-01) [19.3,31.5] Years old:2.07E-02(1.78E-01) (31.5,47.1] Years old:9.10E-01(9.27E-01) (47.1,84.7] Years old:2.17E-02(1.68E-01) Escitalopram:4.33E-03(4.34E-02) Citalopram:4.58E-01(5.57E-01) |
|  | C16:2 | All Participants:-0.10(0.06) Females:-0.21(0.08) Males:0.10(0.11) [19.3,31.5] Years old:-0.24(0.10) (31.5,47.1] Years old:0.06(0.11) (47.1,84.7] Years old:-0.12(0.11) Escitalopram:-0.11(0.08) Citalopram:-0.07(0.12) | All Participants:1.11E-01(2.03E-01) Females:5.51E-03(4.08E-02) Males:3.77E-01(4.95E-01) [19.3,31.5] Years old:2.08E-02(1.78E-01) (31.5,47.1] Years old:6.07E-01(6.92E-01) (47.1,84.7] Years old:2.96E-01(6.93E-01) Escitalopram:1.39E-01(3.33E-01) Citalopram:5.22E-01(5.91E-01) |
|  | C14:2-OH | All Participants:-0.05(0.04) Females:-0.09(0.05) Males:0.01(0.08) [19.3,31.5] Years old:-0.07(0.08) (31.5,47.1] Years old:0.04(0.08) (47.1,84.7] Years old:-0.12(0.06) Escitalopram:-0.06(0.05) Citalopram:-0.03(0.09) | All Participants:2.22E-01(3.35E-01) Females:7.80E-02(2.52E-01) Males:8.93E-01(8.93E-01) [19.3,31.5] Years old:3.82E-01(7.82E-01) (31.5,47.1] Years old:6.45E-01(7.06E-01) (47.1,84.7] Years old:5.46E-02(2.87E-01) Escitalopram:2.00E-01(3.93E-01) Citalopram:7.52E-01(7.85E-01) |
|  | C16-OH | All Participants:-0.01(0.05) Females:-0.06(0.07) Males:0.09(0.09) [19.3,31.5] Years old:-0.02(0.10) (31.5,47.1] Years old:0.10(0.10) (47.1,84.7] Years old:-0.11(0.09) Escitalopram:-0.07(0.07) Citalopram:0.13(0.09) | All Participants:8.85E-01(9.33E-01) Females:3.67E-01(6.79E-01) Males:3.30E-01(4.59E-01) [19.3,31.5] Years old:8.70E-01(9.09E-01) (31.5,47.1] Years old:2.91E-01(4.05E-01) (47.1,84.7] Years old:2.02E-01(5.93E-01) Escitalopram:3.36E-01(5.26E-01) Citalopram:1.49E-01(2.64E-01) |
| **Amino Acids** | **Arginine** | All Participants:0.29(0.09) Females:0.22(0.10) Males:0.43(0.16) [19.3,31.5] Years old:0.15(0.12) (31.5,47.1] Years old:0.54(0.18) (47.1,84.7] Years old:0.19(0.14) Escitalopram:0.25(0.10) Citalopram:0.39(0.15) | All Participants:7.11E-04(5.46E-03) Females:2.98E-02(1.52E-01) Males:8.12E-03(5.09E-02) [19.3,31.5] Years old:2.15E-01(6.14E-01) (31.5,47.1] Years old:3.18E-03(5.30E-02) (47.1,84.7] Years old:1.80E-01(5.93E-01) Escitalopram:1.60E-02(8.96E-02) Citalopram:1.03E-02(7.88E-02) |
|  | **Proline** | All Participants:0.13(0.04) Females:0.09(0.06) Males:0.20(0.06) [19.3,31.5] Years old:0.14(0.07) (31.5,47.1] Years old:0.16(0.08) (47.1,84.7] Years old:0.08(0.08) Escitalopram:0.11(0.05) Citalopram:0.17(0.09) | All Participants:3.41E-03(1.71E-02) Females:1.24E-01(3.62E-01) Males:2.73E-03(3.71E-02) [19.3,31.5] Years old:5.62E-02(3.05E-01) (31.5,47.1] Years old:3.38E-02(1.11E-01) (47.1,84.7] Years old:3.10E-01(7.02E-01) Escitalopram:2.24E-02(9.82E-02) Citalopram:7.33E-02(1.78E-01) |
|  | Tyrosine | All Participants:0.10(0.04) Females:0.03(0.05) Males:0.21(0.06) [19.3,31.5] Years old:0.09(0.06) (31.5,47.1] Years old:0.20(0.07) (47.1,84.7] Years old:0.01(0.07) Escitalopram:0.09(0.05) Citalopram:0.12(0.08) | All Participants:1.54E-02(5.08E-02) Females:5.15E-01(7.48E-01) Males:1.01E-03(1.64E-02) [19.3,31.5] Years old:1.62E-01(5.21E-01) (31.5,47.1] Years old:1.09E-02(7.38E-02) (47.1,84.7] Years old:9.01E-01(9.52E-01) Escitalopram:5.57E-02(1.89E-01) Citalopram:1.46E-01(2.62E-01) |
|  | Citrulline | All Participants:0.09(0.04) Females:0.07(0.05) Males:0.14(0.07) [19.3,31.5] Years old:0.09(0.06) (31.5,47.1] Years old:0.23(0.07) (47.1,84.7] Years old:-0.04(0.08) Escitalopram:0.06(0.05) Citalopram:0.17(0.07) | All Participants:1.84E-02(5.77E-02) Females:1.63E-01(4.28E-01) Males:4.09E-02(1.31E-01) [19.3,31.5] Years old:1.34E-01(4.98E-01) (31.5,47.1] Years old:1.46E-03(3.97E-02) (47.1,84.7] Years old:6.42E-01(8.51E-01) Escitalopram:2.07E-01(4.00E-01) Citalopram:1.44E-02(8.64E-02) |
|  | Phenylalanine | All Participants:0.06(0.03) Females:0.02(0.04) Males:0.14(0.05) [19.3,31.5] Years old:0.06(0.05) (31.5,47.1] Years old:0.11(0.06) (47.1,84.7] Years old:0.02(0.06) Escitalopram:0.04(0.04) Citalopram:0.12(0.06) | All Participants:4.34E-02(1.12E-01) Females:5.90E-01(7.79E-01) Males:6.00E-03(4.97E-02) [19.3,31.5] Years old:2.32E-01(6.42E-01) (31.5,47.1] Years old:5.86E-02(1.46E-01) (47.1,84.7] Years old:7.25E-01(8.95E-01) Escitalopram:2.57E-01(4.72E-01) Citalopram:6.18E-02(1.68E-01) |
|  | Histidine | All Participants:0.05(0.03) Females:0.02(0.03) Males:0.11(0.05) [19.3,31.5] Years old:0.05(0.05) (31.5,47.1] Years old:0.09(0.05) (47.1,84.7] Years old:0.01(0.05) Escitalopram:0.02(0.03) Citalopram:0.14(0.06) | All Participants:6.10E-02(1.38E-01) Females:5.26E-01(7.48E-01) Males:2.55E-02(1.09E-01) [19.3,31.5] Years old:2.78E-01(6.79E-01) (31.5,47.1] Years old:7.33E-02(1.64E-01) (47.1,84.7] Years old:7.94E-01(9.05E-01) Escitalopram:5.71E-01(7.41E-01) Citalopram:2.45E-02(9.33E-02) |
|  | Ornithine | All Participants:-0.11(0.06) Females:-0.13(0.08) Males:-0.08(0.09) [19.3,31.5] Years old:-0.05(0.10) (31.5,47.1] Years old:-0.10(0.12) (47.1,84.7] Years old:-0.19(0.10) Escitalopram:-0.07(0.07) Citalopram:-0.20(0.11) | All Participants:6.41E-02(1.43E-01) Females:9.60E-02(2.95E-01) Males:3.95E-01(5.07E-01) [19.3,31.5] Years old:6.22E-01(8.52E-01) (31.5,47.1] Years old:4.10E-01(4.98E-01) (47.1,84.7] Years old:6.00E-02(2.88E-01) Escitalopram:3.09E-01(5.14E-01) Citalopram:5.89E-02(1.68E-01) |
|  | Methionine | All Participants:0.09(0.05) Females:0.05(0.07) Males:0.17(0.09) [19.3,31.5] Years old:0.10(0.08) (31.5,47.1] Years old:0.24(0.10) (47.1,84.7] Years old:-0.07(0.10) Escitalopram:0.08(0.06) Citalopram:0.13(0.10) | All Participants:9.07E-02(1.80E-01) Females:4.58E-01(7.17E-01) Males:7.65E-02(1.83E-01) [19.3,31.5] Years old:1.78E-01(5.28E-01) (31.5,47.1] Years old:1.53E-02(8.01E-02) (47.1,84.7] Years old:4.77E-01(8.20E-01) Escitalopram:2.43E-01(4.51E-01) Citalopram:1.93E-01(3.15E-01) |
|  | Glycine | All Participants:0.05(0.03) Females:0.04(0.04) Males:0.06(0.05) [19.3,31.5] Years old:0.05(0.06) (31.5,47.1] Years old:0.09(0.05) (47.1,84.7] Years old:0.00(0.05) Escitalopram:0.04(0.04) Citalopram:0.07(0.06) | All Participants:1.14E-01(2.06E-01) Females:3.12E-01(6.22E-01) Males:1.85E-01(3.02E-01) [19.3,31.5] Years old:4.01E-01(7.84E-01) (31.5,47.1] Years old:4.58E-02(1.31E-01) (47.1,84.7] Years old:9.60E-01(9.71E-01) Escitalopram:2.82E-01(4.98E-01) Citalopram:2.21E-01(3.33E-01) |
|  | Threonine | All Participants:-0.06(0.04) Females:-0.10(0.05) Males:0.01(0.06) [19.3,31.5] Years old:-0.02(0.07) (31.5,47.1] Years old:0.03(0.06) (47.1,84.7] Years old:-0.19(0.07) Escitalopram:-0.08(0.05) Citalopram:-0.01(0.06) | All Participants:1.16E-01(2.07E-01) Females:4.35E-02(1.74E-01) Males:8.10E-01(8.30E-01) [19.3,31.5] Years old:7.11E-01(8.62E-01) (31.5,47.1] Years old:6.22E-01(7.00E-01) (47.1,84.7] Years old:7.50E-03(1.20E-01) Escitalopram:8.70E-02(2.36E-01) Citalopram:8.69E-01(8.80E-01) |
|  | Tryptophan | All Participants:0.06(0.04) Females:0.01(0.05) Males:0.13(0.06) [19.3,31.5] Years old:0.04(0.06) (31.5,47.1] Years old:0.17(0.07) (47.1,84.7] Years old:-0.04(0.06) Escitalopram:-0.00(0.04) Citalopram:0.20(0.07) | All Participants:1.44E-01(2.43E-01) Females:8.11E-01(8.95E-01) Males:2.36E-02(1.07E-01) [19.3,31.5] Years old:5.46E-01(8.40E-01) (31.5,47.1] Years old:2.04E-02(8.82E-02) (47.1,84.7] Years old:4.88E-01(8.20E-01) Escitalopram:9.28E-01(9.70E-01) Citalopram:6.27E-03(6.26E-02) |
|  | Glutamate | All Participants:0.04(0.05) Females:0.02(0.07) Males:0.09(0.09) [19.3,31.5] Years old:0.07(0.09) (31.5,47.1] Years old:0.06(0.09) (47.1,84.7] Years old:0.01(0.09) Escitalopram:0.02(0.07) Citalopram:0.10(0.09) | All Participants:4.10E-01(5.26E-01) Females:7.51E-01(8.84E-01) Males:3.43E-01(4.64E-01) [19.3,31.5] Years old:4.73E-01(8.21E-01) (31.5,47.1] Years old:5.61E-01(6.53E-01) (47.1,84.7] Years old:9.15E-01(9.52E-01) Escitalopram:7.48E-01(8.52E-01) Citalopram:2.80E-01(3.97E-01) |
|  | Alanine | All Participants:-0.03(0.04) Females:-0.01(0.05) Males:-0.05(0.05) [19.3,31.5] Years old:-0.03(0.06) (31.5,47.1] Years old:-0.00(0.06) (47.1,84.7] Years old:-0.04(0.06) Escitalopram:-0.05(0.04) Citalopram:0.02(0.06) | All Participants:4.73E-01(5.82E-01) Females:8.38E-01(9.04E-01) Males:3.20E-01(4.49E-01) [19.3,31.5] Years old:6.35E-01(8.55E-01) (31.5,47.1] Years old:9.43E-01(9.48E-01) (47.1,84.7] Years old:4.81E-01(8.20E-01) Escitalopram:2.78E-01(4.98E-01) Citalopram:7.15E-01(7.52E-01) |
|  | Valine | All Participants:0.03(0.04) Females:-0.04(0.05) Males:0.16(0.06) [19.3,31.5] Years old:0.07(0.05) (31.5,47.1] Years old:0.11(0.07) (47.1,84.7] Years old:-0.10(0.07) Escitalopram:0.03(0.04) Citalopram:0.02(0.08) | All Participants:4.75E-01(5.82E-01) Females:3.72E-01(6.79E-01) Males:1.05E-02(6.10E-02) [19.3,31.5] Years old:1.75E-01(5.28E-01) (31.5,47.1] Years old:1.49E-01(2.34E-01) (47.1,84.7] Years old:1.69E-01(5.86E-01) Escitalopram:4.57E-01(6.75E-01) Citalopram:8.43E-01(8.59E-01) |
|  | Isoleucine | All Participants:0.03(0.05) Females:-0.03(0.07) Males:0.15(0.08) [19.3,31.5] Years old:0.08(0.07) (31.5,47.1] Years old:0.14(0.09) (47.1,84.7] Years old:-0.13(0.10) Escitalopram:0.04(0.06) Citalopram:0.01(0.10) | All Participants:5.21E-01(6.16E-01) Females:6.07E-01(7.80E-01) Males:5.71E-02(1.54E-01) [19.3,31.5] Years old:2.50E-01(6.66E-01) (31.5,47.1] Years old:1.01E-01(1.82E-01) (47.1,84.7] Years old:1.95E-01(5.93E-01) Escitalopram:4.61E-01(6.75E-01) Citalopram:9.42E-01(9.48E-01) |
|  | Aspartate | All Participants:-0.05(0.08) Females:-0.05(0.10) Males:-0.05(0.13) [19.3,31.5] Years old:0.02(0.12) (31.5,47.1] Years old:-0.08(0.15) (47.1,84.7] Years old:-0.09(0.15) Escitalopram:-0.06(0.10) Citalopram:-0.03(0.15) | All Participants:5.31E-01(6.19E-01) Females:6.16E-01(7.80E-01) Males:7.12E-01(7.68E-01) [19.3,31.5] Years old:8.67E-01(9.09E-01) (31.5,47.1] Years old:5.78E-01(6.64E-01) (47.1,84.7] Years old:5.49E-01(8.28E-01) Escitalopram:5.48E-01(7.32E-01) Citalopram:8.27E-01(8.48E-01) |
|  | Serine | All Participants:0.01(0.03) Females:-0.03(0.04) Males:0.07(0.05) [19.3,31.5] Years old:0.02(0.05) (31.5,47.1] Years old:0.04(0.05) (47.1,84.7] Years old:-0.05(0.06) Escitalopram:-0.00(0.04) Citalopram:0.04(0.06) | All Participants:8.16E-01(8.87E-01) Females:5.20E-01(7.48E-01) Males:1.48E-01(2.59E-01) [19.3,31.5] Years old:6.51E-01(8.57E-01) (31.5,47.1] Years old:3.68E-01(4.72E-01) (47.1,84.7] Years old:4.28E-01(7.93E-01) Escitalopram:8.96E-01(9.49E-01) Citalopram:5.33E-01(6.00E-01) |
|  | Lysine | All Participants:0.00(0.04) Females:-0.03(0.05) Males:0.07(0.07) [19.3,31.5] Years old:0.00(0.06) (31.5,47.1] Years old:0.11(0.08) (47.1,84.7] Years old:-0.10(0.08) Escitalopram:-0.00(0.05) Citalopram:0.02(0.08) | All Participants:9.07E-01(9.33E-01) Females:5.28E-01(7.48E-01) Males:2.66E-01(3.86E-01) [19.3,31.5] Years old:9.37E-01(9.66E-01) (31.5,47.1] Years old:1.49E-01(2.34E-01) (47.1,84.7] Years old:1.94E-01(5.93E-01) Escitalopram:9.61E-01(9.80E-01) Citalopram:7.86E-01(8.16E-01) |
|  | Glutamine | All Participants:0.00(0.03) Females:-0.04(0.04) Males:0.07(0.05) [19.3,31.5] Years old:-0.04(0.05) (31.5,47.1] Years old:0.08(0.05) (47.1,84.7] Years old:-0.02(0.05) Escitalopram:-0.01(0.03) Citalopram:0.04(0.06) | All Participants:9.10E-01(9.33E-01) Females:3.11E-01(6.22E-01) Males:1.38E-01(2.45E-01) [19.3,31.5] Years old:3.61E-01(7.65E-01) (31.5,47.1] Years old:1.43E-01(2.33E-01) (47.1,84.7] Years old:6.93E-01(8.83E-01) Escitalopram:7.03E-01(8.44E-01) Citalopram:4.78E-01(5.68E-01) |
|  | Asparagine | All Participants:0.00(0.03) Females:-0.00(0.04) Males:0.01(0.05) [19.3,31.5] Years old:0.02(0.05) (31.5,47.1] Years old:0.04(0.06) (47.1,84.7] Years old:-0.05(0.06) Escitalopram:-0.01(0.04) Citalopram:0.04(0.06) | All Participants:9.36E-01(9.53E-01) Females:9.70E-01(9.82E-01) Males:8.48E-01(8.59E-01) [19.3,31.5] Years old:6.82E-01(8.57E-01) (31.5,47.1] Years old:5.06E-01(5.93E-01) (47.1,84.7] Years old:3.87E-01(7.47E-01) Escitalopram:7.18E-01(8.44E-01) Citalopram:4.90E-01(5.75E-01) |
| **Biogenic Amines** | **Serotonin** | All Participants:-1.77(0.17) Females:-1.82(0.21) Males:-1.68(0.29) [19.3,31.5] Years old:-1.75(0.29) (31.5,47.1] Years old:-1.81(0.29) (47.1,84.7] Years old:-1.74(0.30) Escitalopram:-1.99(0.19) Citalopram:-1.23(0.33) | All Participants:1.55E-21(2.53E-19) Females:2.36E-15(3.84E-13) Males:1.25E-07(2.04E-05) [19.3,31.5] Years old:2.77E-08(4.51E-06) (31.5,47.1] Years old:2.67E-08(4.36E-06) (47.1,84.7] Years old:1.56E-07(2.55E-05) Escitalopram:1.33E-19(2.17E-17) Citalopram:3.70E-04(1.51E-02) |
|  | **Sarcosine** | All Participants:-0.34(0.09) Females:-0.27(0.11) Males:-0.47(0.13) [19.3,31.5] Years old:-0.16(0.15) (31.5,47.1] Years old:-0.41(0.15) (47.1,84.7] Years old:-0.47(0.15) Escitalopram:-0.23(0.10) Citalopram:-0.63(0.16) | All Participants:9.53E-05(1.55E-03) Females:1.52E-02(9.19E-02) Males:4.70E-04(9.57E-03) [19.3,31.5] Years old:3.08E-01(7.00E-01) (31.5,47.1] Years old:9.42E-03(7.32E-02) (47.1,84.7] Years old:2.06E-03(4.81E-02) Escitalopram:2.78E-02(1.11E-01) Citalopram:1.45E-04(7.86E-03) |
|  | **Methioninesulfoxide** | All Participants:0.31(0.08) Females:0.33(0.10) Males:0.27(0.14) [19.3,31.5] Years old:0.49(0.14) (31.5,47.1] Years old:0.35(0.14) (47.1,84.7] Years old:0.07(0.16) Escitalopram:0.27(0.10) Citalopram:0.38(0.16) | All Participants:3.34E-04(3.61E-03) Females:2.10E-03(2.21E-02) Males:6.22E-02(1.56E-01) [19.3,31.5] Years old:6.36E-04(1.30E-02) (31.5,47.1] Years old:1.33E-02(8.01E-02) (47.1,84.7] Years old:6.67E-01(8.70E-01) Escitalopram:7.07E-03(4.61E-02) Citalopram:1.59E-02(8.64E-02) |
|  | trans-4-Hydroxyproline | All Participants:0.15(0.07) Females:0.08(0.08) Males:0.29(0.12) [19.3,31.5] Years old:0.15(0.11) (31.5,47.1] Years old:0.34(0.13) (47.1,84.7] Years old:-0.03(0.12) Escitalopram:0.11(0.08) Citalopram:0.25(0.12) | All Participants:2.50E-02(7.53E-02) Females:3.39E-01(6.55E-01) Males:1.60E-02(8.97E-02) [19.3,31.5] Years old:1.55E-01(5.21E-01) (31.5,47.1] Years old:8.43E-03(7.32E-02) (47.1,84.7] Years old:8.05E-01(9.11E-01) Escitalopram:1.75E-01(3.60E-01) Citalopram:3.56E-02(1.21E-01) |
|  | Kynurenine | All Participants:0.07(0.04) Females:0.03(0.05) Males:0.13(0.06) [19.3,31.5] Years old:-0.02(0.06) (31.5,47.1] Years old:0.16(0.07) (47.1,84.7] Years old:0.06(0.07) Escitalopram:0.01(0.05) Citalopram:0.20(0.07) | All Participants:9.28E-02(1.82E-01) Females:5.75E-01(7.68E-01) Males:3.73E-02(1.27E-01) [19.3,31.5] Years old:7.26E-01(8.62E-01) (31.5,47.1] Years old:3.48E-02(1.11E-01) (47.1,84.7] Years old:3.57E-01(7.37E-01) Escitalopram:8.16E-01(8.99E-01) Citalopram:8.66E-03(7.05E-02) |
|  | Spermine | All Participants:-0.09(0.06) Females:-0.06(0.07) Males:-0.14(0.09) [19.3,31.5] Years old:-0.14(0.10) (31.5,47.1] Years old:-0.11(0.09) (47.1,84.7] Years old:-0.02(0.10) Escitalopram:-0.13(0.07) Citalopram:0.00(0.10) | All Participants:1.08E-01(2.00E-01) Females:4.02E-01(6.79E-01) Males:1.18E-01(2.29E-01) [19.3,31.5] Years old:1.58E-01(5.21E-01) (31.5,47.1] Years old:2.02E-01(3.02E-01) (47.1,84.7] Years old:8.71E-01(9.46E-01) Escitalopram:5.87E-02(1.91E-01) Citalopram:9.65E-01(9.65E-01) |
|  | Asymmetric dimethylarginine | All Participants:-0.05(0.04) Females:-0.04(0.04) Males:-0.06(0.06) [19.3,31.5] Years old:-0.06(0.06) (31.5,47.1] Years old:0.06(0.07) (47.1,84.7] Years old:-0.15(0.06) Escitalopram:-0.06(0.04) Citalopram:-0.03(0.07) | All Participants:1.70E-01(2.74E-01) Females:3.13E-01(6.22E-01) Males:3.50E-01(4.64E-01) [19.3,31.5] Years old:3.17E-01(7.07E-01) (31.5,47.1] Years old:3.52E-01(4.63E-01) (47.1,84.7] Years old:1.21E-02(1.43E-01) Escitalopram:1.64E-01(3.53E-01) Citalopram:7.03E-01(7.44E-01) |
|  | Taurine | All Participants:0.07(0.06) Females:0.03(0.06) Males:0.13(0.11) [19.3,31.5] Years old:0.07(0.10) (31.5,47.1] Years old:0.05(0.09) (47.1,84.7] Years old:0.09(0.10) Escitalopram:-0.03(0.07) Citalopram:0.29(0.10) | All Participants:2.30E-01(3.41E-01) Females:6.22E-01(7.80E-01) Males:2.24E-01(3.37E-01) [19.3,31.5] Years old:4.66E-01(8.21E-01) (31.5,47.1] Years old:6.35E-01(7.04E-01) (47.1,84.7] Years old:3.88E-01(7.47E-01) Escitalopram:6.81E-01(8.40E-01) Citalopram:3.48E-03(4.06E-02) |
|  | Spermidine | All Participants:-0.06(0.05) Females:-0.01(0.06) Males:-0.14(0.10) [19.3,31.5] Years old:-0.05(0.08) (31.5,47.1] Years old:-0.09(0.10) (47.1,84.7] Years old:-0.04(0.08) Escitalopram:-0.14(0.06) Citalopram:0.14(0.08) | All Participants:2.33E-01(3.43E-01) Females:8.14E-01(8.95E-01) Males:1.37E-01(2.45E-01) [19.3,31.5] Years old:5.56E-01(8.40E-01) (31.5,47.1] Years old:3.62E-01(4.72E-01) (47.1,84.7] Years old:6.18E-01(8.51E-01) Escitalopram:2.29E-02(9.82E-02) Citalopram:9.51E-02(2.08E-01) |
|  | Symmetric dimethylarginine | All Participants:-0.03(0.03) Females:-0.03(0.03) Males:-0.03(0.05) [19.3,31.5] Years old:-0.01(0.04) (31.5,47.1] Years old:0.00(0.04) (47.1,84.7] Years old:-0.07(0.05) Escitalopram:-0.07(0.03) Citalopram:0.06(0.04) | All Participants:2.88E-01(4.01E-01) Females:3.89E-01(6.79E-01) Males:5.35E-01(6.25E-01) [19.3,31.5] Years old:7.57E-01(8.69E-01) (31.5,47.1] Years old:9.41E-01(9.48E-01) (47.1,84.7] Years old:1.40E-01(5.53E-01) Escitalopram:4.03E-02(1.49E-01) Citalopram:1.71E-01(2.93E-01) |
|  | alpha-Aminoadipic acid | All Participants:0.05(0.08) Females:-0.03(0.11) Males:0.20(0.11) [19.3,31.5] Years old:0.08(0.11) (31.5,47.1] Years old:0.29(0.14) (47.1,84.7] Years old:-0.20(0.17) Escitalopram:0.02(0.09) Citalopram:0.14(0.17) | All Participants:5.17E-01(6.16E-01) Females:7.77E-01(8.94E-01) Males:5.79E-02(1.54E-01) [19.3,31.5] Years old:5.05E-01(8.25E-01) (31.5,47.1] Years old:3.79E-02(1.19E-01) (47.1,84.7] Years old:2.31E-01(6.39E-01) Escitalopram:8.43E-01(9.16E-01) Citalopram:4.31E-01(5.36E-01) |
|  | Putrescine | All Participants:-0.03(0.07) Females:0.01(0.09) Males:-0.11(0.12) [19.3,31.5] Years old:-0.04(0.09) (31.5,47.1] Years old:0.12(0.13) (47.1,84.7] Years old:-0.17(0.15) Escitalopram:-0.11(0.08) Citalopram:0.15(0.15) | All Participants:6.75E-01(7.59E-01) Females:8.98E-01(9.50E-01) Males:4.01E-01(5.10E-01) [19.3,31.5] Years old:6.41E-01(8.56E-01) (31.5,47.1] Years old:3.82E-01(4.87E-01) (47.1,84.7] Years old:2.75E-01(6.93E-01) Escitalopram:1.98E-01(3.93E-01) Citalopram:3.29E-01(4.40E-01) |
|  | Creatinine | All Participants:-0.00(0.02) Females:-0.01(0.03) Males:0.02(0.04) [19.3,31.5] Years old:0.02(0.04) (31.5,47.1] Years old:0.05(0.04) (47.1,84.7] Years old:-0.07(0.05) Escitalopram:-0.02(0.03) Citalopram:0.05(0.04) | All Participants:9.85E-01(9.85E-01) Females:6.44E-01(7.96E-01) Males:5.81E-01(6.59E-01) [19.3,31.5] Years old:5.32E-01(8.40E-01) (31.5,47.1] Years old:2.33E-01(3.45E-01) (47.1,84.7] Years old:1.11E-01(4.75E-01) Escitalopram:4.81E-01(6.75E-01) Citalopram:2.47E-01(3.64E-01) |
| **Sphingolipids** | **SM C18:1** | All Participants:-0.10(0.03) Females:-0.12(0.04) Males:-0.07(0.05) [19.3,31.5] Years old:-0.11(0.06) (31.5,47.1] Years old:-0.06(0.06) (47.1,84.7] Years old:-0.12(0.05) Escitalopram:-0.12(0.04) Citalopram:-0.05(0.05) | All Participants:1.83E-03(1.03E-02) Females:4.23E-03(3.45E-02) Males:1.79E-01(2.98E-01) [19.3,31.5] Years old:4.79E-02(2.79E-01) (31.5,47.1] Years old:2.98E-01(4.09E-01) (47.1,84.7] Years old:1.49E-02(1.52E-01) Escitalopram:1.84E-03(2.32E-02) Citalopram:3.92E-01(5.03E-01) |
|  | **SM C24:0** | All Participants:0.09(0.03) Females:0.08(0.04) Males:0.11(0.06) [19.3,31.5] Years old:0.06(0.05) (31.5,47.1] Years old:0.14(0.06) (47.1,84.7] Years old:0.07(0.05) Escitalopram:0.07(0.04) Citalopram:0.14(0.05) | All Participants:4.14E-03(1.93E-02) Females:3.23E-02(1.55E-01) Males:5.95E-02(1.54E-01) [19.3,31.5] Years old:2.76E-01(6.79E-01) (31.5,47.1] Years old:1.41E-02(8.01E-02) (47.1,84.7] Years old:2.00E-01(5.93E-01) Escitalopram:7.82E-02(2.20E-01) Citalopram:8.05E-03(6.91E-02) |
|  | SM C18:0 | All Participants:-0.06(0.03) Females:-0.08(0.04) Males:-0.03(0.05) [19.3,31.5] Years old:-0.07(0.05) (31.5,47.1] Years old:-0.03(0.06) (47.1,84.7] Years old:-0.09(0.05) Escitalopram:-0.08(0.04) Citalopram:-0.01(0.05) | All Participants:3.55E-02(9.65E-02) Females:3.45E-02(1.59E-01) Males:4.96E-01(5.86E-01) [19.3,31.5] Years old:2.12E-01(6.14E-01) (31.5,47.1] Years old:5.74E-01(6.63E-01) (47.1,84.7] Years old:5.46E-02(2.87E-01) Escitalopram:2.16E-02(9.82E-02) Citalopram:7.93E-01(8.18E-01) |
|  | SM C20:2 | All Participants:-0.08(0.04) Females:-0.12(0.05) Males:-0.01(0.06) [19.3,31.5] Years old:-0.11(0.07) (31.5,47.1] Years old:-0.06(0.07) (47.1,84.7] Years old:-0.07(0.06) Escitalopram:-0.08(0.05) Citalopram:-0.07(0.06) | All Participants:3.68E-02(9.84E-02) Females:1.45E-02(9.12E-02) Males:8.86E-01(8.91E-01) [19.3,31.5] Years old:1.03E-01(4.27E-01) (31.5,47.1] Years old:3.93E-01(4.93E-01) (47.1,84.7] Years old:2.88E-01(6.93E-01) Escitalopram:8.07E-02(2.23E-01) Citalopram:2.56E-01(3.67E-01) |
|  | SM (OH) C14:1 | All Participants:0.05(0.03) Females:0.02(0.03) Males:0.10(0.05) [19.3,31.5] Years old:0.04(0.04) (31.5,47.1] Years old:0.10(0.05) (47.1,84.7] Years old:0.00(0.04) Escitalopram:0.02(0.03) Citalopram:0.11(0.05) | All Participants:6.98E-02(1.50E-01) Females:5.62E-01(7.68E-01) Males:3.28E-02(1.23E-01) [19.3,31.5] Years old:3.84E-01(7.82E-01) (31.5,47.1] Years old:5.46E-02(1.45E-01) (47.1,84.7] Years old:9.24E-01(9.54E-01) Escitalopram:4.78E-01(6.75E-01) Citalopram:2.03E-02(9.19E-02) |
|  | SM (OH) C22:1 | All Participants:0.05(0.03) Females:0.03(0.04) Males:0.10(0.06) [19.3,31.5] Years old:0.04(0.05) (31.5,47.1] Years old:0.11(0.06) (47.1,84.7] Years old:0.00(0.05) Escitalopram:0.02(0.04) Citalopram:0.13(0.05) | All Participants:8.08E-02(1.66E-01) Females:4.12E-01(6.79E-01) Males:8.84E-02(2.05E-01) [19.3,31.5] Years old:3.61E-01(7.65E-01) (31.5,47.1] Years old:6.82E-02(1.57E-01) (47.1,84.7] Years old:9.44E-01(9.62E-01) Escitalopram:5.73E-01(7.41E-01) Citalopram:1.54E-02(8.64E-02) |
|  | SM (OH) C24:1 | All Participants:0.04(0.03) Females:0.03(0.04) Males:0.05(0.05) [19.3,31.5] Years old:0.02(0.05) (31.5,47.1] Years old:0.09(0.05) (47.1,84.7] Years old:-0.01(0.05) Escitalopram:0.01(0.03) Citalopram:0.10(0.05) | All Participants:2.06E-01(3.19E-01) Females:4.49E-01(7.16E-01) Males:2.85E-01(4.11E-01) [19.3,31.5] Years old:6.34E-01(8.55E-01) (31.5,47.1] Years old:8.77E-02(1.70E-01) (47.1,84.7] Years old:8.93E-01(9.52E-01) Escitalopram:7.83E-01(8.86E-01) Citalopram:5.81E-02(1.68E-01) |
|  | SM C16:0 | All Participants:0.03(0.03) Females:0.01(0.03) Males:0.07(0.04) [19.3,31.5] Years old:0.01(0.04) (31.5,47.1] Years old:0.09(0.05) (47.1,84.7] Years old:-0.01(0.04) Escitalopram:0.00(0.03) Citalopram:0.09(0.04) | All Participants:2.21E-01(3.35E-01) Females:8.11E-01(8.95E-01) Males:1.03E-01(2.26E-01) [19.3,31.5] Years old:7.94E-01(8.87E-01) (31.5,47.1] Years old:7.69E-02(1.67E-01) (47.1,84.7] Years old:8.88E-01(9.52E-01) Escitalopram:8.70E-01(9.35E-01) Citalopram:4.16E-02(1.30E-01) |
|  | SM C26:0 | All Participants:0.03(0.03) Females:0.03(0.04) Males:0.04(0.05) [19.3,31.5] Years old:0.03(0.05) (31.5,47.1] Years old:0.09(0.05) (47.1,84.7] Years old:-0.03(0.05) Escitalopram:0.00(0.03) Citalopram:0.10(0.06) | All Participants:2.80E-01(3.93E-01) Females:4.71E-01(7.25E-01) Males:4.12E-01(5.16E-01) [19.3,31.5] Years old:5.19E-01(8.30E-01) (31.5,47.1] Years old:9.21E-02(1.74E-01) (47.1,84.7] Years old:5.94E-01(8.42E-01) Escitalopram:9.04E-01(9.51E-01) Citalopram:9.20E-02(2.05E-01) |
|  | SM (OH) C22:2 | All Participants:0.03(0.03) Females:-0.00(0.03) Males:0.08(0.05) [19.3,31.5] Years old:0.02(0.05) (31.5,47.1] Years old:0.09(0.06) (47.1,84.7] Years old:-0.02(0.04) Escitalopram:-0.01(0.03) Citalopram:0.12(0.05) | All Participants:3.09E-01(4.26E-01) Females:9.41E-01(9.64E-01) Males:8.86E-02(2.05E-01) [19.3,31.5] Years old:6.77E-01(8.57E-01) (31.5,47.1] Years old:1.04E-01(1.86E-01) (47.1,84.7] Years old:5.95E-01(8.42E-01) Escitalopram:8.10E-01(8.99E-01) Citalopram:3.02E-02(1.07E-01) |
|  | SM (OH) C16:1 | All Participants:-0.02(0.03) Females:-0.05(0.04) Males:0.02(0.05) [19.3,31.5] Years old:-0.04(0.05) (31.5,47.1] Years old:0.03(0.06) (47.1,84.7] Years old:-0.07(0.04) Escitalopram:-0.05(0.03) Citalopram:0.03(0.05) | All Participants:3.86E-01(5.07E-01) Females:1.84E-01(4.75E-01) Males:7.50E-01(7.93E-01) [19.3,31.5] Years old:4.69E-01(8.21E-01) (31.5,47.1] Years old:6.28E-01(7.01E-01) (47.1,84.7] Years old:1.46E-01(5.53E-01) Escitalopram:1.60E-01(3.53E-01) Citalopram:5.22E-01(5.91E-01) |
|  | SM C16:1 | All Participants:0.02(0.03) Females:-0.01(0.03) Males:0.06(0.04) [19.3,31.5] Years old:-0.01(0.04) (31.5,47.1] Years old:0.08(0.05) (47.1,84.7] Years old:-0.03(0.04) Escitalopram:-0.01(0.03) Citalopram:0.08(0.05) | All Participants:5.61E-01(6.42E-01) Females:7.79E-01(8.94E-01) Males:2.04E-01(3.20E-01) [19.3,31.5] Years old:8.49E-01(9.09E-01) (31.5,47.1] Years old:1.14E-01(1.99E-01) (47.1,84.7] Years old:5.16E-01(8.27E-01) Escitalopram:7.27E-01(8.47E-01) Citalopram:9.58E-02(2.08E-01) |
|  | SM C24:1 | All Participants:-0.00(0.03) Females:-0.02(0.03) Males:0.04(0.05) [19.3,31.5] Years old:-0.02(0.05) (31.5,47.1] Years old:0.05(0.05) (47.1,84.7] Years old:-0.03(0.04) Escitalopram:-0.03(0.03) Citalopram:0.08(0.05) | All Participants:9.55E-01(9.61E-01) Females:4.80E-01(7.32E-01) Males:4.05E-01(5.11E-01) [19.3,31.5] Years old:6.75E-01(8.57E-01) (31.5,47.1] Years old:3.66E-01(4.72E-01) (47.1,84.7] Years old:5.03E-01(8.21E-01) Escitalopram:2.90E-01(4.98E-01) Citalopram:1.34E-01(2.48E-01) |
|  | SM C26:1 | All Participants:0.00(0.03) Females:-0.02(0.04) Males:0.03(0.05) [19.3,31.5] Years old:-0.02(0.05) (31.5,47.1] Years old:0.07(0.05) (47.1,84.7] Years old:-0.05(0.05) Escitalopram:-0.03(0.03) Citalopram:0.08(0.06) | All Participants:9.55E-01(9.61E-01) Females:6.53E-01(8.00E-01) Males:4.91E-01(5.86E-01) [19.3,31.5] Years old:7.48E-01(8.65E-01) (31.5,47.1] Years old:1.65E-01(2.56E-01) (47.1,84.7] Years old:3.02E-01(6.93E-01) Escitalopram:3.58E-01(5.50E-01) Citalopram:1.44E-01(2.61E-01) |
| **PC aa** | **PC aa C36:1** | All Participants:0.15(0.04) Females:0.12(0.05) Males:0.20(0.07) [19.3,31.5] Years old:0.09(0.07) (31.5,47.1] Years old:0.21(0.08) (47.1,84.7] Years old:0.14(0.06) Escitalopram:0.14(0.05) Citalopram:0.17(0.08) | All Participants:4.26E-04(3.79E-03) Females:1.82E-02(1.06E-01) Males:8.09E-03(5.09E-02) [19.3,31.5] Years old:1.74E-01(5.28E-01) (31.5,47.1] Years old:1.71E-02(8.20E-02) (47.1,84.7] Years old:2.09E-02(1.68E-01) Escitalopram:5.06E-03(4.34E-02) Citalopram:3.39E-02(1.17E-01) |
|  | **PC aa C30:0** | All Participants:0.17(0.05) Females:0.12(0.06) Males:0.24(0.08) [19.3,31.5] Years old:0.17(0.08) (31.5,47.1] Years old:0.23(0.10) (47.1,84.7] Years old:0.10(0.07) Escitalopram:0.15(0.06) Citalopram:0.20(0.09) | All Participants:4.41E-04(3.79E-03) Females:3.51E-02(1.59E-01) Males:2.59E-03(3.71E-02) [19.3,31.5] Years old:3.06E-02(2.01E-01) (31.5,47.1] Years old:2.11E-02(8.82E-02) (47.1,84.7] Years old:1.45E-01(5.53E-01) Escitalopram:6.83E-03(4.61E-02) Citalopram:2.46E-02(9.33E-02) |
|  | **PC aa C42:2** | All Participants:0.09(0.03) Females:0.07(0.03) Males:0.13(0.05) [19.3,31.5] Years old:0.04(0.04) (31.5,47.1] Years old:0.19(0.05) (47.1,84.7] Years old:0.05(0.05) Escitalopram:0.08(0.03) Citalopram:0.12(0.05) | All Participants:7.54E-04(5.46E-03) Females:2.65E-02(1.44E-01) Males:9.61E-03(5.80E-02) [19.3,31.5] Years old:4.14E-01(7.84E-01) (31.5,47.1] Years old:2.50E-04(1.36E-02) (47.1,84.7] Years old:2.63E-01(6.81E-01) Escitalopram:1.70E-02(8.96E-02) Citalopram:1.06E-02(7.88E-02) |
|  | **PC aa C34:4** | All Participants:0.16(0.05) Females:0.12(0.06) Males:0.24(0.08) [19.3,31.5] Years old:0.21(0.09) (31.5,47.1] Years old:0.24(0.09) (47.1,84.7] Years old:0.03(0.07) Escitalopram:0.13(0.06) Citalopram:0.25(0.09) | All Participants:7.70E-04(5.46E-03) Females:4.29E-02(1.74E-01) Males:4.55E-03(4.66E-02) [19.3,31.5] Years old:1.66E-02(1.60E-01) (31.5,47.1] Years old:1.03E-02(7.32E-02) (47.1,84.7] Years old:6.12E-01(8.51E-01) Escitalopram:2.75E-02(1.11E-01) Citalopram:6.53E-03(6.26E-02) |
|  | **PC aa C34:3** | All Participants:0.13(0.04) Females:0.09(0.05) Males:0.22(0.08) [19.3,31.5] Years old:0.12(0.07) (31.5,47.1] Years old:0.21(0.08) (47.1,84.7] Years old:0.07(0.06) Escitalopram:0.13(0.05) Citalopram:0.15(0.08) | All Participants:1.00E-03(6.12E-03) Females:6.72E-02(2.28E-01) Males:4.37E-03(4.66E-02) [19.3,31.5] Years old:6.44E-02(3.14E-01) (31.5,47.1] Years old:1.42E-02(8.01E-02) (47.1,84.7] Years old:2.39E-01(6.41E-01) Escitalopram:8.22E-03(5.15E-02) Citalopram:5.43E-02(1.61E-01) |
|  | **PC aa C40:2** | All Participants:0.10(0.03) Females:0.07(0.03) Males:0.15(0.05) [19.3,31.5] Years old:0.05(0.05) (31.5,47.1] Years old:0.16(0.05) (47.1,84.7] Years old:0.08(0.05) Escitalopram:0.10(0.04) Citalopram:0.09(0.05) | All Participants:1.00E-03(6.12E-03) Females:4.48E-02(1.74E-01) Males:7.56E-03(5.09E-02) [19.3,31.5] Years old:3.31E-01(7.29E-01) (31.5,47.1] Years old:1.95E-03(4.54E-02) (47.1,84.7] Years old:1.15E-01(4.79E-01) Escitalopram:5.86E-03(4.34E-02) Citalopram:7.49E-02(1.79E-01) |
|  | **PC aa C38:6** | All Participants:-0.08(0.03) Females:-0.10(0.03) Males:-0.04(0.05) [19.3,31.5] Years old:-0.10(0.05) (31.5,47.1] Years old:-0.03(0.06) (47.1,84.7] Years old:-0.11(0.04) Escitalopram:-0.09(0.03) Citalopram:-0.05(0.05) | All Participants:3.84E-03(1.84E-02) Females:2.85E-03(2.58E-02) Males:3.89E-01(5.07E-01) [19.3,31.5] Years old:3.08E-02(2.01E-01) (31.5,47.1] Years old:6.50E-01(7.06E-01) (47.1,84.7] Years old:6.08E-03(1.20E-01) Escitalopram:5.04E-03(4.34E-02) Citalopram:3.32E-01(4.41E-01) |
|  | **PC aa C24:0** | All Participants:0.12(0.04) Females:0.09(0.05) Males:0.17(0.07) [19.3,31.5] Years old:0.08(0.07) (31.5,47.1] Years old:0.26(0.09) (47.1,84.7] Years old:0.02(0.06) Escitalopram:0.10(0.05) Citalopram:0.16(0.10) | All Participants:4.93E-03(2.00E-02) Females:8.03E-02(2.52E-01) Males:1.81E-02(9.52E-02) [19.3,31.5] Years old:2.39E-01(6.49E-01) (31.5,47.1] Years old:3.52E-03(5.30E-02) (47.1,84.7] Years old:7.66E-01(9.03E-01) Escitalopram:2.75E-02(1.11E-01) Citalopram:8.87E-02(2.01E-01) |
|  | **PC aa C28:1** | All Participants:0.08(0.03) Females:0.04(0.03) Males:0.15(0.05) [19.3,31.5] Years old:0.08(0.05) (31.5,47.1] Years old:0.12(0.06) (47.1,84.7] Years old:0.03(0.05) Escitalopram:0.06(0.04) Citalopram:0.13(0.05) | All Participants:6.90E-03(2.62E-02) Females:2.49E-01(5.57E-01) Males:6.18E-03(4.97E-02) [19.3,31.5] Years old:7.78E-02(3.52E-01) (31.5,47.1] Years old:4.28E-02(1.27E-01) (47.1,84.7] Years old:4.77E-01(8.20E-01) Escitalopram:9.21E-02(2.46E-01) Citalopram:1.52E-02(8.64E-02) |
|  | **PC aa C36:6** | All Participants:0.11(0.04) Females:0.08(0.05) Males:0.18(0.07) [19.3,31.5] Years old:0.12(0.08) (31.5,47.1] Years old:0.20(0.09) (47.1,84.7] Years old:0.02(0.06) Escitalopram:0.08(0.05) Citalopram:0.20(0.08) | All Participants:9.60E-03(3.48E-02) Females:1.50E-01(4.07E-01) Males:1.80E-02(9.52E-02) [19.3,31.5] Years old:1.07E-01(4.27E-01) (31.5,47.1] Years old:2.07E-02(8.82E-02) (47.1,84.7] Years old:8.14E-01(9.15E-01) Escitalopram:1.36E-01(3.32E-01) Citalopram:1.43E-02(8.64E-02) |
|  | **PC aa C42:4** | All Participants:0.07(0.03) Females:0.05(0.03) Males:0.11(0.05) [19.3,31.5] Years old:0.04(0.05) (31.5,47.1] Years old:0.13(0.05) (47.1,84.7] Years old:0.05(0.04) Escitalopram:0.06(0.03) Citalopram:0.10(0.05) | All Participants:1.11E-02(3.92E-02) Females:1.35E-01(3.73E-01) Males:3.10E-02(1.20E-01) [19.3,31.5] Years old:4.31E-01(7.98E-01) (31.5,47.1] Years old:1.62E-02(8.01E-02) (47.1,84.7] Years old:2.94E-01(6.93E-01) Escitalopram:7.13E-02(2.08E-01) Citalopram:6.30E-02(1.68E-01) |
|  | PC aa C36:3 | All Participants:0.04(0.02) Females:0.03(0.02) Males:0.07(0.03) [19.3,31.5] Years old:0.04(0.03) (31.5,47.1] Years old:0.08(0.03) (47.1,84.7] Years old:0.02(0.02) Escitalopram:0.04(0.02) Citalopram:0.06(0.04) | All Participants:1.56E-02(5.08E-02) Females:1.91E-01(4.83E-01) Males:2.49E-02(1.09E-01) [19.3,31.5] Years old:2.53E-01(6.66E-01) (31.5,47.1] Years old:2.60E-02(1.01E-01) (47.1,84.7] Years old:5.33E-01(8.27E-01) Escitalopram:7.15E-02(2.08E-01) Citalopram:1.10E-01(2.31E-01) |
|  | PC aa C32:1 | All Participants:0.13(0.06) Females:0.09(0.07) Males:0.22(0.10) [19.3,31.5] Years old:0.06(0.10) (31.5,47.1] Years old:0.22(0.12) (47.1,84.7] Years old:0.12(0.09) Escitalopram:0.13(0.07) Citalopram:0.14(0.12) | All Participants:2.28E-02(7.02E-02) Females:2.22E-01(5.24E-01) Males:3.86E-02(1.27E-01) [19.3,31.5] Years old:5.16E-01(8.30E-01) (31.5,47.1] Years old:6.35E-02(1.50E-01) (47.1,84.7] Years old:1.94E-01(5.93E-01) Escitalopram:5.36E-02(1.86E-01) Citalopram:2.32E-01(3.47E-01) |
|  | PC aa C36:2 | All Participants:0.03(0.02) Females:0.02(0.02) Males:0.06(0.03) [19.3,31.5] Years old:0.02(0.03) (31.5,47.1] Years old:0.06(0.03) (47.1,84.7] Years old:0.01(0.02) Escitalopram:0.03(0.02) Citalopram:0.03(0.03) | All Participants:3.40E-02(9.55E-02) Females:3.90E-01(6.79E-01) Males:2.19E-02(1.02E-01) [19.3,31.5] Years old:3.94E-01(7.84E-01) (31.5,47.1] Years old:3.37E-02(1.11E-01) (47.1,84.7] Years old:5.56E-01(8.31E-01) Escitalopram:7.56E-02(2.16E-01) Citalopram:2.57E-01(3.67E-01) |
|  | PC aa C38:3 | All Participants:0.08(0.04) Females:0.06(0.05) Males:0.13(0.07) [19.3,31.5] Years old:0.05(0.07) (31.5,47.1] Years old:0.14(0.08) (47.1,84.7] Years old:0.06(0.06) Escitalopram:0.08(0.05) Citalopram:0.10(0.08) | All Participants:3.97E-02(1.04E-01) Females:2.44E-01(5.57E-01) Males:7.45E-02(1.81E-01) [19.3,31.5] Years old:4.73E-01(8.21E-01) (31.5,47.1] Years old:8.02E-02(1.68E-01) (47.1,84.7] Years old:3.26E-01(7.19E-01) Escitalopram:1.06E-01(2.74E-01) Citalopram:2.08E-01(3.20E-01) |
|  | PC aa C36:5 | All Participants:0.09(0.04) Females:0.05(0.05) Males:0.16(0.08) [19.3,31.5] Years old:0.12(0.07) (31.5,47.1] Years old:0.17(0.08) (47.1,84.7] Years old:-0.03(0.07) Escitalopram:0.06(0.05) Citalopram:0.16(0.08) | All Participants:4.70E-02(1.19E-01) Females:3.82E-01(6.79E-01) Males:3.91E-02(1.27E-01) [19.3,31.5] Years old:8.69E-02(3.83E-01) (31.5,47.1] Years old:4.44E-02(1.29E-01) (47.1,84.7] Years old:6.91E-01(8.83E-01) Escitalopram:2.93E-01(4.98E-01) Citalopram:4.11E-02(1.30E-01) |
|  | PC aa C42:6 | All Participants:0.06(0.03) Females:0.04(0.04) Males:0.09(0.06) [19.3,31.5] Years old:0.01(0.06) (31.5,47.1] Years old:0.12(0.06) (47.1,84.7] Years old:0.05(0.05) Escitalopram:0.05(0.04) Citalopram:0.08(0.06) | All Participants:6.56E-02(1.45E-01) Females:3.10E-01(6.22E-01) Males:1.03E-01(2.26E-01) [19.3,31.5] Years old:8.27E-01(9.00E-01) (31.5,47.1] Years old:6.18E-02(1.48E-01) (47.1,84.7] Years old:3.20E-01(7.15E-01) Escitalopram:1.85E-01(3.73E-01) Citalopram:1.86E-01(3.06E-01) |
|  | PC aa C40:4 | All Participants:0.07(0.04) Females:0.03(0.04) Males:0.13(0.07) [19.3,31.5] Years old:0.04(0.07) (31.5,47.1] Years old:0.11(0.07) (47.1,84.7] Years old:0.06(0.06) Escitalopram:0.06(0.04) Citalopram:0.09(0.07) | All Participants:6.67E-02(1.45E-01) Females:4.53E-01(7.16E-01) Males:5.29E-02(1.54E-01) [19.3,31.5] Years old:5.65E-01(8.40E-01) (31.5,47.1] Years old:1.30E-01(2.16E-01) (47.1,84.7] Years old:3.01E-01(6.93E-01) Escitalopram:1.77E-01(3.61E-01) Citalopram:2.05E-01(3.20E-01) |
|  | PC aa C34:2 | All Participants:0.03(0.01) Females:0.01(0.02) Males:0.06(0.03) [19.3,31.5] Years old:0.02(0.03) (31.5,47.1] Years old:0.05(0.03) (47.1,84.7] Years old:0.01(0.02) Escitalopram:0.03(0.02) Citalopram:0.03(0.03) | All Participants:7.42E-02(1.57E-01) Females:5.75E-01(7.68E-01) Males:3.32E-02(1.23E-01) [19.3,31.5] Years old:5.40E-01(8.40E-01) (31.5,47.1] Years old:5.52E-02(1.45E-01) (47.1,84.7] Years old:6.41E-01(8.51E-01) Escitalopram:1.24E-01(3.10E-01) Citalopram:3.72E-01(4.82E-01) |
|  | PC aa C34:1 | All Participants:0.03(0.02) Females:0.01(0.02) Males:0.06(0.03) [19.3,31.5] Years old:0.01(0.03) (31.5,47.1] Years old:0.06(0.03) (47.1,84.7] Years old:0.01(0.02) Escitalopram:0.03(0.02) Citalopram:0.03(0.03) | All Participants:7.63E-02(1.59E-01) Females:5.68E-01(7.68E-01) Males:3.59E-02(1.27E-01) [19.3,31.5] Years old:6.89E-01(8.57E-01) (31.5,47.1] Years old:4.90E-02(1.38E-01) (47.1,84.7] Years old:5.69E-01(8.42E-01) Escitalopram:1.50E-01(3.47E-01) Citalopram:3.12E-01(4.27E-01) |
|  | PC aa C40:3 | All Participants:0.05(0.03) Females:0.03(0.03) Males:0.09(0.05) [19.3,31.5] Years old:0.02(0.05) (31.5,47.1] Years old:0.11(0.05) (47.1,84.7] Years old:0.02(0.05) Escitalopram:0.03(0.03) Citalopram:0.09(0.05) | All Participants:8.14E-02(1.66E-01) Females:4.21E-01(6.87E-01) Males:8.93E-02(2.05E-01) [19.3,31.5] Years old:6.83E-01(8.57E-01) (31.5,47.1] Years old:2.80E-02(1.04E-01) (47.1,84.7] Years old:7.36E-01(9.02E-01) Escitalopram:3.29E-01(5.26E-01) Citalopram:9.93E-02(2.13E-01) |
|  | PC aa C40:6 | All Participants:-0.05(0.03) Females:-0.09(0.04) Males:0.01(0.05) [19.3,31.5] Years old:-0.11(0.05) (31.5,47.1] Years old:0.02(0.06) (47.1,84.7] Years old:-0.06(0.05) Escitalopram:-0.04(0.04) Citalopram:-0.06(0.06) | All Participants:1.07E-01(2.00E-01) Females:2.85E-02(1.50E-01) Males:7.76E-01(8.10E-01) [19.3,31.5] Years old:2.89E-02(2.01E-01) (31.5,47.1] Years old:7.02E-01(7.50E-01) (47.1,84.7] Years old:2.15E-01(6.16E-01) Escitalopram:2.12E-01(4.02E-01) Citalopram:3.16E-01(4.29E-01) |
|  | PC aa C36:4 | All Participants:0.02(0.02) Females:0.01(0.02) Males:0.05(0.03) [19.3,31.5] Years old:0.01(0.03) (31.5,47.1] Years old:0.06(0.03) (47.1,84.7] Years old:0.00(0.02) Escitalopram:0.02(0.02) Citalopram:0.03(0.03) | All Participants:1.18E-01(2.10E-01) Females:6.44E-01(7.96E-01) Males:5.86E-02(1.54E-01) [19.3,31.5] Years old:6.84E-01(8.57E-01) (31.5,47.1] Years old:5.54E-02(1.45E-01) (47.1,84.7] Years old:8.53E-01(9.39E-01) Escitalopram:2.09E-01(4.00E-01) Citalopram:3.64E-01(4.78E-01) |
|  | PC aa C42:0 | All Participants:-0.03(0.02) Females:-0.06(0.03) Males:0.01(0.04) [19.3,31.5] Years old:-0.04(0.04) (31.5,47.1] Years old:0.04(0.04) (47.1,84.7] Years old:-0.09(0.04) Escitalopram:-0.06(0.03) Citalopram:0.02(0.05) | All Participants:1.75E-01(2.80E-01) Females:5.45E-02(1.98E-01) Males:7.17E-01(7.69E-01) [19.3,31.5] Years old:2.96E-01(6.95E-01) (31.5,47.1] Years old:3.25E-01(4.38E-01) (47.1,84.7] Years old:3.16E-02(2.06E-01) Escitalopram:4.45E-02(1.61E-01) Citalopram:5.96E-01(6.44E-01) |
|  | PC aa C38:4 | All Participants:0.02(0.02) Females:0.01(0.02) Males:0.05(0.03) [19.3,31.5] Years old:-0.01(0.03) (31.5,47.1] Years old:0.06(0.04) (47.1,84.7] Years old:0.01(0.03) Escitalopram:0.02(0.02) Citalopram:0.02(0.04) | All Participants:2.25E-01(3.36E-01) Females:7.92E-01(8.95E-01) Males:1.27E-01(2.41E-01) [19.3,31.5] Years old:8.61E-01(9.09E-01) (31.5,47.1] Years old:9.91E-02(1.82E-01) (47.1,84.7] Years old:6.93E-01(8.83E-01) Escitalopram:3.08E-01(5.14E-01) Citalopram:5.16E-01(5.91E-01) |
|  | PC aa C32:3 | All Participants:-0.03(0.03) Females:-0.07(0.03) Males:0.04(0.05) [19.3,31.5] Years old:-0.07(0.05) (31.5,47.1] Years old:0.04(0.06) (47.1,84.7] Years old:-0.06(0.05) Escitalopram:-0.05(0.03) Citalopram:0.03(0.05) | All Participants:3.31E-01(4.54E-01) Females:4.63E-02(1.76E-01) Males:4.43E-01(5.38E-01) [19.3,31.5] Years old:1.67E-01(5.22E-01) (31.5,47.1] Years old:4.72E-01(5.70E-01) (47.1,84.7] Years old:1.89E-01(5.93E-01) Escitalopram:1.33E-01(3.28E-01) Citalopram:5.81E-01(6.38E-01) |
|  | PC aa C32:0 | All Participants:0.02(0.03) Females:0.01(0.04) Males:0.05(0.05) [19.3,31.5] Years old:0.02(0.05) (31.5,47.1] Years old:0.08(0.05) (47.1,84.7] Years old:-0.03(0.04) Escitalopram:0.00(0.03) Citalopram:0.07(0.05) | All Participants:4.04E-01(5.23E-01) Females:8.02E-01(8.95E-01) Males:2.92E-01(4.18E-01) [19.3,31.5] Years old:7.17E-01(8.62E-01) (31.5,47.1] Years old:1.41E-01(2.33E-01) (47.1,84.7] Years old:5.43E-01(8.27E-01) Escitalopram:9.36E-01(9.72E-01) Citalopram:1.73E-01(2.93E-01) |
|  | PC aa C42:5 | All Participants:0.02(0.03) Females:0.01(0.04) Males:0.05(0.05) [19.3,31.5] Years old:-0.01(0.05) (31.5,47.1] Years old:0.09(0.06) (47.1,84.7] Years old:-0.01(0.04) Escitalopram:0.01(0.04) Citalopram:0.05(0.06) | All Participants:4.41E-01(5.49E-01) Females:8.18E-01(8.95E-01) Males:3.49E-01(4.64E-01) [19.3,31.5] Years old:8.59E-01(9.09E-01) (31.5,47.1] Years old:1.49E-01(2.34E-01) (47.1,84.7] Years old:9.09E-01(9.52E-01) Escitalopram:6.89E-01(8.40E-01) Citalopram:4.40E-01(5.39E-01) |
|  | PC aa C40:5 | All Participants:0.02(0.04) Females:-0.01(0.04) Males:0.08(0.06) [19.3,31.5] Years old:-0.00(0.06) (31.5,47.1] Years old:0.07(0.07) (47.1,84.7] Years old:-0.00(0.05) Escitalopram:0.02(0.04) Citalopram:0.04(0.07) | All Participants:5.22E-01(6.16E-01) Females:7.87E-01(8.95E-01) Males:1.88E-01(3.03E-01) [19.3,31.5] Years old:9.92E-01(9.92E-01) (31.5,47.1] Years old:2.91E-01(4.05E-01) (47.1,84.7] Years old:9.39E-01(9.62E-01) Escitalopram:6.91E-01(8.40E-01) Citalopram:5.83E-01(6.38E-01) |
|  | PC aa C38:5 | All Participants:-0.01(0.03) Females:-0.04(0.03) Males:0.04(0.05) [19.3,31.5] Years old:-0.04(0.05) (31.5,47.1] Years old:0.06(0.05) (47.1,84.7] Years old:-0.05(0.04) Escitalopram:-0.03(0.03) Citalopram:0.03(0.05) | All Participants:6.97E-01(7.78E-01) Females:2.81E-01(6.02E-01) Males:4.73E-01(5.71E-01) [19.3,31.5] Years old:4.30E-01(7.98E-01) (31.5,47.1] Years old:2.43E-01(3.57E-01) (47.1,84.7] Years old:2.04E-01(5.93E-01) Escitalopram:4.09E-01(6.12E-01) Citalopram:5.97E-01(6.44E-01) |
|  | PC aa C38:0 | All Participants:0.01(0.02) Females:-0.01(0.03) Males:0.04(0.04) [19.3,31.5] Years old:-0.03(0.04) (31.5,47.1] Years old:0.07(0.05) (47.1,84.7] Years old:-0.02(0.04) Escitalopram:-0.04(0.03) Citalopram:0.11(0.05) | All Participants:8.16E-01(8.87E-01) Females:6.84E-01(8.14E-01) Males:3.50E-01(4.64E-01) [19.3,31.5] Years old:4.49E-01(8.21E-01) (31.5,47.1] Years old:1.28E-01(2.15E-01) (47.1,84.7] Years old:6.30E-01(8.51E-01) Escitalopram:1.67E-01(3.53E-01) Citalopram:1.92E-02(9.19E-02) |
|  | PC aa C42:1 | All Participants:-0.00(0.02) Females:-0.03(0.03) Males:0.05(0.04) [19.3,31.5] Years old:-0.04(0.04) (31.5,47.1] Years old:0.07(0.04) (47.1,84.7] Years old:-0.05(0.04) Escitalopram:-0.03(0.03) Citalopram:0.06(0.05) | All Participants:9.06E-01(9.33E-01) Females:2.94E-01(6.14E-01) Males:2.07E-01(3.22E-01) [19.3,31.5] Years old:4.06E-01(7.84E-01) (31.5,47.1] Years old:9.62E-02(1.78E-01) (47.1,84.7] Years old:2.85E-01(6.93E-01) Escitalopram:3.32E-01(5.26E-01) Citalopram:2.07E-01(3.20E-01) |
| **PC ae** | **PC ae C34:3** | All Participants:0.17(0.03) Females:0.14(0.04) Males:0.21(0.06) [19.3,31.5] Years old:0.16(0.06) (31.5,47.1] Years old:0.23(0.06) (47.1,84.7] Years old:0.11(0.05) Escitalopram:0.10(0.04) Citalopram:0.33(0.07) | All Participants:1.29E-06(4.21E-05) Females:7.89E-04(1.29E-02) Males:3.98E-04(9.28E-03) [19.3,31.5] Years old:8.38E-03(9.75E-02) (31.5,47.1] Years old:5.99E-04(1.95E-02) (47.1,84.7] Years old:3.42E-02(2.06E-01) Escitalopram:1.04E-02(6.31E-02) Citalopram:3.81E-06(6.21E-04) |
|  | **PC ae C38:2** | All Participants:0.13(0.04) Females:0.12(0.04) Males:0.15(0.06) [19.3,31.5] Years old:0.11(0.06) (31.5,47.1] Years old:0.19(0.07) (47.1,84.7] Years old:0.09(0.05) Escitalopram:0.12(0.04) Citalopram:0.16(0.07) | All Participants:3.46E-04(3.61E-03) Females:7.12E-03(5.05E-02) Males:1.98E-02(9.96E-02) [19.3,31.5] Years old:7.37E-02(3.43E-01) (31.5,47.1] Years old:9.70E-03(7.32E-02) (47.1,84.7] Years old:9.91E-02(4.49E-01) Escitalopram:5.81E-03(4.34E-02) Citalopram:2.35E-02(9.33E-02) |
|  | **PC ae C36:3** | All Participants:0.12(0.03) Females:0.10(0.04) Males:0.16(0.06) [19.3,31.5] Years old:0.11(0.06) (31.5,47.1] Years old:0.18(0.06) (47.1,84.7] Years old:0.07(0.05) Escitalopram:0.07(0.04) Citalopram:0.23(0.07) | All Participants:3.55E-04(3.61E-03) Females:1.88E-02(1.06E-01) Males:5.65E-03(4.97E-02) [19.3,31.5] Years old:6.44E-02(3.14E-01) (31.5,47.1] Years old:6.73E-03(6.85E-02) (47.1,84.7] Years old:1.54E-01(5.57E-01) Escitalopram:6.17E-02(1.91E-01) Citalopram:6.88E-04(1.87E-02) |
|  | **PC ae C34:2** | All Participants:0.11(0.03) Females:0.09(0.04) Males:0.14(0.05) [19.3,31.5] Years old:0.12(0.06) (31.5,47.1] Years old:0.17(0.06) (47.1,84.7] Years old:0.04(0.05) Escitalopram:0.07(0.04) Citalopram:0.20(0.07) | All Participants:8.88E-04(6.03E-03) Females:3.08E-02(1.52E-01) Males:8.07E-03(5.09E-02) [19.3,31.5] Years old:4.34E-02(2.62E-01) (31.5,47.1] Years old:9.17E-03(7.32E-02) (47.1,84.7] Years old:3.91E-01(7.47E-01) Escitalopram:5.34E-02(1.86E-01) Citalopram:3.84E-03(4.18E-02) |
|  | **PC ae C30:0** | All Participants:0.11(0.03) Females:0.09(0.04) Males:0.16(0.06) [19.3,31.5] Years old:0.14(0.06) (31.5,47.1] Years old:0.15(0.07) (47.1,84.7] Years old:0.05(0.05) Escitalopram:0.09(0.04) Citalopram:0.15(0.06) | All Participants:1.01E-03(6.12E-03) Females:4.04E-02(1.73E-01) Males:7.18E-03(5.09E-02) [19.3,31.5] Years old:1.60E-02(1.60E-01) (31.5,47.1] Years old:3.16E-02(1.11E-01) (47.1,84.7] Years old:3.56E-01(7.37E-01) Escitalopram:1.97E-02(9.78E-02) Citalopram:1.65E-02(8.68E-02) |
|  | **PC ae C42:2** | All Participants:0.08(0.03) Females:0.05(0.04) Males:0.12(0.05) [19.3,31.5] Years old:0.06(0.05) (31.5,47.1] Years old:0.15(0.06) (47.1,84.7] Years old:0.03(0.05) Escitalopram:0.05(0.03) Citalopram:0.13(0.06) | All Participants:1.13E-02(3.92E-02) Females:1.57E-01(4.21E-01) Males:2.13E-02(1.02E-01) [19.3,31.5] Years old:2.75E-01(6.79E-01) (31.5,47.1] Years old:1.18E-02(7.71E-02) (47.1,84.7] Years old:5.84E-01(8.42E-01) Escitalopram:1.21E-01(3.07E-01) Citalopram:2.76E-02(1.01E-01) |
|  | PC ae C36:2 | All Participants:0.07(0.03) Females:0.05(0.04) Males:0.10(0.05) [19.3,31.5] Years old:0.05(0.05) (31.5,47.1] Years old:0.14(0.06) (47.1,84.7] Years old:0.02(0.04) Escitalopram:0.05(0.04) Citalopram:0.12(0.05) | All Participants:1.65E-02(5.27E-02) Females:1.30E-01(3.67E-01) Males:5.64E-02(1.54E-01) [19.3,31.5] Years old:2.99E-01(6.95E-01) (31.5,47.1] Years old:1.62E-02(8.01E-02) (47.1,84.7] Years old:7.11E-01(8.92E-01) Escitalopram:1.51E-01(3.47E-01) Citalopram:2.31E-02(9.33E-02) |
|  | PC ae C36:5 | All Participants:0.07(0.03) Females:0.05(0.04) Males:0.10(0.05) [19.3,31.5] Years old:0.07(0.05) (31.5,47.1] Years old:0.14(0.06) (47.1,84.7] Years old:-0.01(0.04) Escitalopram:-0.01(0.03) Citalopram:0.24(0.05) | All Participants:2.64E-02(7.84E-02) Females:1.92E-01(4.83E-01) Males:5.80E-02(1.54E-01) [19.3,31.5] Years old:1.63E-01(5.21E-01) (31.5,47.1] Years old:1.55E-02(8.01E-02) (47.1,84.7] Years old:7.47E-01(9.02E-01) Escitalopram:8.72E-01(9.35E-01) Citalopram:3.47E-05(2.83E-03) |
|  | PC ae C36:0 | All Participants:0.06(0.03) Females:0.04(0.03) Males:0.08(0.04) [19.3,31.5] Years old:0.02(0.05) (31.5,47.1] Years old:0.12(0.05) (47.1,84.7] Years old:0.04(0.04) Escitalopram:0.03(0.03) Citalopram:0.12(0.05) | All Participants:3.52E-02(9.65E-02) Females:2.16E-01(5.20E-01) Males:6.03E-02(1.54E-01) [19.3,31.5] Years old:7.22E-01(8.62E-01) (31.5,47.1] Years old:2.33E-02(9.26E-02) (47.1,84.7] Years old:3.93E-01(7.47E-01) Escitalopram:3.32E-01(5.26E-01) Citalopram:2.24E-02(9.33E-02) |
|  | PC ae C32:2 | All Participants:0.05(0.03) Females:0.03(0.03) Males:0.09(0.04) [19.3,31.5] Years old:0.02(0.04) (31.5,47.1] Years old:0.14(0.05) (47.1,84.7] Years old:-0.01(0.04) Escitalopram:0.02(0.03) Citalopram:0.13(0.04) | All Participants:4.74E-02(1.19E-01) Females:4.07E-01(6.79E-01) Males:3.69E-02(1.27E-01) [19.3,31.5] Years old:6.02E-01(8.45E-01) (31.5,47.1] Years old:5.45E-03(6.08E-02) (47.1,84.7] Years old:7.63E-01(9.03E-01) Escitalopram:5.89E-01(7.55E-01) Citalopram:3.02E-03(4.06E-02) |
|  | PC ae C40:1 | All Participants:0.06(0.03) Females:0.04(0.04) Males:0.11(0.05) [19.3,31.5] Years old:0.06(0.05) (31.5,47.1] Years old:0.13(0.06) (47.1,84.7] Years old:-0.01(0.05) Escitalopram:0.03(0.04) Citalopram:0.15(0.06) | All Participants:5.21E-02(1.27E-01) Females:3.41E-01(6.55E-01) Males:5.70E-02(1.54E-01) [19.3,31.5] Years old:2.63E-01(6.79E-01) (31.5,47.1] Years old:2.89E-02(1.05E-01) (47.1,84.7] Years old:9.17E-01(9.52E-01) Escitalopram:4.78E-01(6.75E-01) Citalopram:1.98E-02(9.19E-02) |
|  | PC ae C36:1 | All Participants:0.06(0.03) Females:0.03(0.04) Males:0.11(0.05) [19.3,31.5] Years old:-0.00(0.05) (31.5,47.1] Years old:0.13(0.06) (47.1,84.7] Years old:0.04(0.04) Escitalopram:0.04(0.04) Citalopram:0.11(0.05) | All Participants:5.25E-02(1.27E-01) Females:4.02E-01(6.79E-01) Males:4.36E-02(1.37E-01) [19.3,31.5] Years old:9.74E-01(9.91E-01) (31.5,47.1] Years old:1.78E-02(8.30E-02) (47.1,84.7] Years old:3.62E-01(7.38E-01) Escitalopram:3.22E-01(5.24E-01) Citalopram:4.06E-02(1.30E-01) |
|  | PC ae C32:1 | All Participants:0.05(0.03) Females:0.04(0.03) Males:0.07(0.05) [19.3,31.5] Years old:0.04(0.04) (31.5,47.1] Years old:0.13(0.06) (47.1,84.7] Years old:-0.01(0.04) Escitalopram:0.02(0.03) Citalopram:0.14(0.05) | All Participants:5.28E-02(1.27E-01) Females:2.17E-01(5.20E-01) Males:1.17E-01(2.29E-01) [19.3,31.5] Years old:3.46E-01(7.51E-01) (31.5,47.1] Years old:2.70E-02(1.02E-01) (47.1,84.7] Years old:8.59E-01(9.39E-01) Escitalopram:5.93E-01(7.55E-01) Citalopram:6.93E-03(6.28E-02) |
|  | PC ae C34:0 | All Participants:0.06(0.03) Females:0.05(0.04) Males:0.09(0.06) [19.3,31.5] Years old:0.03(0.06) (31.5,47.1] Years old:0.12(0.07) (47.1,84.7] Years old:0.04(0.05) Escitalopram:0.03(0.04) Citalopram:0.13(0.06) | All Participants:6.05E-02(1.38E-01) Females:2.49E-01(5.57E-01) Males:1.16E-01(2.29E-01) [19.3,31.5] Years old:5.63E-01(8.40E-01) (31.5,47.1] Years old:8.71E-02(1.70E-01) (47.1,84.7] Years old:4.45E-01(7.97E-01) Escitalopram:3.74E-01(5.69E-01) Citalopram:4.54E-02(1.37E-01) |
|  | PC ae C42:1 | All Participants:0.05(0.03) Females:0.03(0.03) Males:0.10(0.05) [19.3,31.5] Years old:0.02(0.05) (31.5,47.1] Years old:0.12(0.05) (47.1,84.7] Years old:0.02(0.05) Escitalopram:0.03(0.03) Citalopram:0.10(0.05) | All Participants:6.09E-02(1.38E-01) Females:4.63E-01(7.19E-01) Males:3.73E-02(1.27E-01) [19.3,31.5] Years old:6.53E-01(8.57E-01) (31.5,47.1] Years old:1.54E-02(8.01E-02) (47.1,84.7] Years old:7.41E-01(9.02E-01) Escitalopram:3.15E-01(5.18E-01) Citalopram:6.51E-02(1.68E-01) |
|  | PC ae C36:4 | All Participants:0.05(0.03) Females:0.03(0.04) Males:0.08(0.05) [19.3,31.5] Years old:0.08(0.05) (31.5,47.1] Years old:0.10(0.05) (47.1,84.7] Years old:-0.03(0.05) Escitalopram:-0.01(0.03) Citalopram:0.18(0.06) | All Participants:9.80E-02(1.90E-01) Females:3.84E-01(6.79E-01) Males:1.16E-01(2.29E-01) [19.3,31.5] Years old:1.46E-01(5.09E-01) (31.5,47.1] Years old:6.65E-02(1.55E-01) (47.1,84.7] Years old:4.82E-01(8.20E-01) Escitalopram:8.13E-01(8.99E-01) Citalopram:3.49E-03(4.06E-02) |
|  | PC ae C38:3 | All Participants:0.05(0.03) Females:0.03(0.04) Males:0.08(0.05) [19.3,31.5] Years old:0.04(0.05) (31.5,47.1] Years old:0.11(0.05) (47.1,84.7] Years old:-0.00(0.05) Escitalopram:0.03(0.04) Citalopram:0.11(0.06) | All Participants:1.00E-01(1.92E-01) Females:3.75E-01(6.79E-01) Males:1.35E-01(2.45E-01) [19.3,31.5] Years old:4.92E-01(8.25E-01) (31.5,47.1] Years old:4.19E-02(1.27E-01) (47.1,84.7] Years old:9.65E-01(9.71E-01) Escitalopram:4.63E-01(6.75E-01) Citalopram:6.28E-02(1.68E-01) |
|  | PC ae C40:2 | All Participants:-0.04(0.02) Females:-0.05(0.03) Males:-0.02(0.04) [19.3,31.5] Years old:-0.03(0.04) (31.5,47.1] Years old:0.02(0.04) (47.1,84.7] Years old:-0.10(0.04) Escitalopram:-0.07(0.03) Citalopram:0.03(0.04) | All Participants:1.22E-01(2.15E-01) Females:1.08E-01(3.19E-01) Males:6.56E-01(7.27E-01) [19.3,31.5] Years old:4.73E-01(8.21E-01) (31.5,47.1] Years old:7.04E-01(7.50E-01) (47.1,84.7] Years old:1.31E-02(1.43E-01) Escitalopram:2.24E-02(9.82E-02) Citalopram:4.88E-01(5.75E-01) |
|  | PC ae C38:6 | All Participants:0.04(0.03) Females:0.02(0.03) Males:0.07(0.04) [19.3,31.5] Years old:0.02(0.05) (31.5,47.1] Years old:0.11(0.05) (47.1,84.7] Years old:-0.02(0.04) Escitalopram:-0.02(0.03) Citalopram:0.17(0.05) | All Participants:1.38E-01(2.34E-01) Females:5.37E-01(7.54E-01) Males:1.08E-01(2.28E-01) [19.3,31.5] Years old:5.80E-01(8.40E-01) (31.5,47.1] Years old:3.40E-02(1.11E-01) (47.1,84.7] Years old:6.57E-01(8.63E-01) Escitalopram:5.67E-01(7.41E-01) Citalopram:6.54E-04(1.87E-02) |
|  | PC ae C40:6 | All Participants:-0.04(0.03) Females:-0.06(0.03) Males:0.01(0.04) [19.3,31.5] Years old:-0.07(0.04) (31.5,47.1] Years old:0.04(0.05) (47.1,84.7] Years old:-0.08(0.04) Escitalopram:-0.07(0.03) Citalopram:0.04(0.05) | All Participants:1.47E-01(2.45E-01) Females:4.82E-02(1.79E-01) Males:8.42E-01(8.57E-01) [19.3,31.5] Years old:9.97E-02(4.27E-01) (31.5,47.1] Years old:3.33E-01(4.45E-01) (47.1,84.7] Years old:6.00E-02(2.88E-01) Escitalopram:2.28E-02(9.82E-02) Citalopram:3.99E-01(5.08E-01) |
|  | PC ae C34:1 | All Participants:0.04(0.03) Females:0.02(0.04) Males:0.08(0.05) [19.3,31.5] Years old:-0.02(0.05) (31.5,47.1] Years old:0.12(0.06) (47.1,84.7] Years old:0.03(0.04) Escitalopram:0.02(0.03) Citalopram:0.09(0.06) | All Participants:1.56E-01(2.57E-01) Females:6.01E-01(7.80E-01) Males:1.06E-01(2.26E-01) [19.3,31.5] Years old:6.94E-01(8.57E-01) (31.5,47.1] Years old:5.16E-02(1.43E-01) (47.1,84.7] Years old:5.39E-01(8.27E-01) Escitalopram:5.35E-01(7.27E-01) Citalopram:1.16E-01(2.33E-01) |
|  | PC ae C42:3 | All Participants:0.04(0.03) Females:0.02(0.03) Males:0.07(0.04) [19.3,31.5] Years old:0.02(0.05) (31.5,47.1] Years old:0.10(0.05) (47.1,84.7] Years old:-0.01(0.05) Escitalopram:0.02(0.03) Citalopram:0.08(0.05) | All Participants:1.68E-01(2.73E-01) Females:5.92E-01(7.79E-01) Males:1.12E-01(2.29E-01) [19.3,31.5] Years old:6.07E-01(8.45E-01) (31.5,47.1] Years old:4.21E-02(1.27E-01) (47.1,84.7] Years old:7.89E-01(9.05E-01) Escitalopram:5.57E-01(7.39E-01) Citalopram:1.18E-01(2.34E-01) |
|  | PC ae C40:5 | All Participants:-0.03(0.02) Females:-0.05(0.03) Males:0.01(0.04) [19.3,31.5] Years old:-0.07(0.04) (31.5,47.1] Years old:0.05(0.04) (47.1,84.7] Years old:-0.07(0.04) Escitalopram:-0.06(0.03) Citalopram:0.04(0.04) | All Participants:1.80E-01(2.86E-01) Females:6.33E-02(2.24E-01) Males:8.09E-01(8.30E-01) [19.3,31.5] Years old:1.07E-01(4.27E-01) (31.5,47.1] Years old:2.52E-01(3.67E-01) (47.1,84.7] Years old:6.43E-02(3.00E-01) Escitalopram:3.53E-02(1.37E-01) Citalopram:4.14E-01(5.23E-01) |
|  | PC ae C42:5 | All Participants:-0.03(0.02) Females:-0.05(0.03) Males:0.01(0.03) [19.3,31.5] Years old:-0.04(0.04) (31.5,47.1] Years old:0.04(0.03) (47.1,84.7] Years old:-0.08(0.04) Escitalopram:-0.05(0.03) Citalopram:0.02(0.04) | All Participants:2.06E-01(3.19E-01) Females:6.48E-02(2.25E-01) Males:6.60E-01(7.27E-01) [19.3,31.5] Years old:2.83E-01(6.79E-01) (31.5,47.1] Years old:2.56E-01(3.70E-01) (47.1,84.7] Years old:3.40E-02(2.06E-01) Escitalopram:6.21E-02(1.91E-01) Citalopram:5.52E-01(6.12E-01) |
|  | PC ae C44:3 | All Participants:0.03(0.03) Females:0.02(0.03) Males:0.06(0.05) [19.3,31.5] Years old:0.05(0.04) (31.5,47.1] Years old:0.09(0.05) (47.1,84.7] Years old:-0.04(0.05) Escitalopram:0.02(0.03) Citalopram:0.07(0.05) | All Participants:2.10E-01(3.22E-01) Females:5.45E-01(7.59E-01) Males:2.10E-01(3.23E-01) [19.3,31.5] Years old:2.80E-01(6.79E-01) (31.5,47.1] Years old:5.70E-02(1.45E-01) (47.1,84.7] Years old:3.68E-01(7.40E-01) Escitalopram:5.42E-01(7.31E-01) Citalopram:1.65E-01(2.86E-01) |
|  | PC ae C44:6 | All Participants:-0.03(0.03) Females:-0.06(0.03) Males:0.03(0.04) [19.3,31.5] Years old:-0.03(0.04) (31.5,47.1] Years old:0.03(0.04) (47.1,84.7] Years old:-0.09(0.05) Escitalopram:-0.06(0.03) Citalopram:0.04(0.04) | All Participants:2.37E-01(3.44E-01) Females:4.49E-02(1.74E-01) Males:4.37E-01(5.35E-01) [19.3,31.5] Years old:4.90E-01(8.25E-01) (31.5,47.1] Years old:3.91E-01(4.93E-01) (47.1,84.7] Years old:4.38E-02(2.46E-01) Escitalopram:5.96E-02(1.91E-01) Citalopram:4.38E-01(5.39E-01) |
|  | PC ae C44:5 | All Participants:-0.03(0.03) Females:-0.06(0.03) Males:0.02(0.04) [19.3,31.5] Years old:-0.03(0.05) (31.5,47.1] Years old:0.03(0.04) (47.1,84.7] Years old:-0.10(0.04) Escitalopram:-0.05(0.03) Citalopram:0.02(0.05) | All Participants:2.49E-01(3.56E-01) Females:7.03E-02(2.34E-01) Males:5.60E-01(6.43E-01) [19.3,31.5] Years old:5.68E-01(8.40E-01) (31.5,47.1] Years old:4.04E-01(4.95E-01) (47.1,84.7] Years old:2.84E-02(2.06E-01) Escitalopram:9.82E-02(2.58E-01) Citalopram:6.36E-01(6.77E-01) |
|  | PC ae C30:2 | All Participants:0.03(0.03) Females:0.00(0.03) Males:0.07(0.05) [19.3,31.5] Years old:0.01(0.04) (31.5,47.1] Years old:0.09(0.05) (47.1,84.7] Years old:-0.01(0.04) Escitalopram:0.01(0.03) Citalopram:0.07(0.05) | All Participants:2.64E-01(3.74E-01) Females:9.19E-01(9.64E-01) Males:1.12E-01(2.29E-01) [19.3,31.5] Years old:8.97E-01(9.31E-01) (31.5,47.1] Years old:8.26E-02(1.70E-01) (47.1,84.7] Years old:7.60E-01(9.03E-01) Escitalopram:7.38E-01(8.52E-01) Citalopram:1.13E-01(2.31E-01) |
|  | PC ae C42:4 | All Participants:0.03(0.03) Females:0.00(0.03) Males:0.07(0.04) [19.3,31.5] Years old:0.02(0.04) (31.5,47.1] Years old:0.09(0.04) (47.1,84.7] Years old:-0.04(0.05) Escitalopram:0.01(0.03) Citalopram:0.07(0.05) | All Participants:3.34E-01(4.54E-01) Females:9.86E-01(9.86E-01) Males:1.15E-01(2.29E-01) [19.3,31.5] Years old:5.82E-01(8.40E-01) (31.5,47.1] Years old:3.33E-02(1.11E-01) (47.1,84.7] Years old:4.43E-01(7.97E-01) Escitalopram:8.39E-01(9.16E-01) Citalopram:1.28E-01(2.44E-01) |
|  | PC ae C38:0 | All Participants:0.03(0.03) Females:0.00(0.04) Males:0.08(0.05) [19.3,31.5] Years old:0.00(0.06) (31.5,47.1] Years old:0.11(0.06) (47.1,84.7] Years old:-0.02(0.05) Escitalopram:0.00(0.04) Citalopram:0.10(0.06) | All Participants:3.45E-01(4.65E-01) Females:9.53E-01(9.71E-01) Males:1.51E-01(2.62E-01) [19.3,31.5] Years old:9.66E-01(9.90E-01) (31.5,47.1] Years old:6.12E-02(1.48E-01) (47.1,84.7] Years old:6.21E-01(8.51E-01) Escitalopram:9.60E-01(9.80E-01) Citalopram:8.62E-02(2.01E-01) |
|  | PC ae C38:5 | All Participants:0.02(0.02) Females:-0.00(0.03) Males:0.05(0.04) [19.3,31.5] Years old:-0.01(0.04) (31.5,47.1] Years old:0.08(0.04) (47.1,84.7] Years old:-0.02(0.04) Escitalopram:-0.04(0.03) Citalopram:0.15(0.05) | All Participants:5.04E-01(6.09E-01) Females:8.93E-01(9.50E-01) Males:2.02E-01(3.19E-01) [19.3,31.5] Years old:8.03E-01(8.87E-01) (31.5,47.1] Years old:7.27E-02(1.64E-01) (47.1,84.7] Years old:5.99E-01(8.42E-01) Escitalopram:1.73E-01(3.60E-01) Citalopram:2.18E-03(3.56E-02) |
|  | PC ae C44:4 | All Participants:0.01(0.02) Females:-0.01(0.03) Males:0.06(0.04) [19.3,31.5] Years old:0.03(0.04) (31.5,47.1] Years old:0.06(0.04) (47.1,84.7] Years old:-0.05(0.05) Escitalopram:-0.00(0.03) Citalopram:0.05(0.05) | All Participants:5.43E-01(6.28E-01) Females:7.54E-01(8.84E-01) Males:1.34E-01(2.45E-01) [19.3,31.5] Years old:5.06E-01(8.25E-01) (31.5,47.1] Years old:9.27E-02(1.74E-01) (47.1,84.7] Years old:2.99E-01(6.93E-01) Escitalopram:9.76E-01(9.83E-01) Citalopram:2.57E-01(3.67E-01) |
|  | PC ae C40:4 | All Participants:0.01(0.02) Females:-0.02(0.03) Males:0.06(0.04) [19.3,31.5] Years old:-0.01(0.04) (31.5,47.1] Years old:0.07(0.04) (47.1,84.7] Years old:-0.05(0.04) Escitalopram:-0.02(0.03) Citalopram:0.06(0.04) | All Participants:8.00E-01(8.83E-01) Females:4.45E-01(7.16E-01) Males:1.61E-01(2.76E-01) [19.3,31.5] Years old:8.29E-01(9.00E-01) (31.5,47.1] Years old:7.70E-02(1.67E-01) (47.1,84.7] Years old:2.40E-01(6.41E-01) Escitalopram:5.27E-01(7.22E-01) Citalopram:1.28E-01(2.44E-01) |
|  | PC ae C38:4 | All Participants:-0.01(0.03) Females:-0.03(0.03) Males:0.03(0.04) [19.3,31.5] Years old:-0.02(0.04) (31.5,47.1] Years old:0.06(0.05) (47.1,84.7] Years old:-0.07(0.04) Escitalopram:-0.04(0.03) Citalopram:0.08(0.04) | All Participants:8.02E-01(8.83E-01) Females:3.88E-01(6.79E-01) Males:4.95E-01(5.86E-01) [19.3,31.5] Years old:7.36E-01(8.62E-01) (31.5,47.1] Years old:1.80E-01(2.74E-01) (47.1,84.7] Years old:1.09E-01(4.75E-01) Escitalopram:1.63E-01(3.53E-01) Citalopram:6.65E-02(1.68E-01) |
|  | PC ae C40:3 | All Participants:0.00(0.02) Females:-0.02(0.03) Males:0.04(0.04) [19.3,31.5] Years old:0.00(0.04) (31.5,47.1] Years old:0.06(0.04) (47.1,84.7] Years old:-0.05(0.04) Escitalopram:-0.02(0.03) Citalopram:0.06(0.04) | All Participants:8.88E-01(9.33E-01) Females:5.73E-01(7.68E-01) Males:3.50E-01(4.64E-01) [19.3,31.5] Years old:9.89E-01(9.92E-01) (31.5,47.1] Years old:1.71E-01(2.63E-01) (47.1,84.7] Years old:2.24E-01(6.31E-01) Escitalopram:5.01E-01(6.98E-01) Citalopram:1.74E-01(2.93E-01) |
| **Lyso PCs** | **lysoPC a C20:4** | All Participants:-0.11(0.04) Females:-0.12(0.04) Males:-0.10(0.06) [19.3,31.5] Years old:-0.12(0.06) (31.5,47.1] Years old:-0.10(0.07) (47.1,84.7] Years old:-0.12(0.06) Escitalopram:-0.15(0.04) Citalopram:-0.03(0.07) | All Participants:1.82E-03(1.03E-02) Females:5.05E-03(3.92E-02) Males:1.38E-01(2.45E-01) [19.3,31.5] Years old:6.56E-02(3.14E-01) (31.5,47.1] Years old:1.28E-01(2.15E-01) (47.1,84.7] Years old:4.35E-02(2.46E-01) Escitalopram:7.82E-04(1.16E-02) Citalopram:6.15E-01(6.59E-01) |
|  | **lysoPC a C28:1** | All Participants:0.09(0.03) Females:0.06(0.04) Males:0.16(0.06) [19.3,31.5] Years old:0.11(0.05) (31.5,47.1] Years old:0.16(0.06) (47.1,84.7] Years old:0.01(0.05) Escitalopram:0.07(0.04) Citalopram:0.16(0.06) | All Participants:2.91E-03(1.53E-02) Females:1.31E-01(3.67E-01) Males:4.96E-03(4.75E-02) [19.3,31.5] Years old:4.09E-02(2.57E-01) (31.5,47.1] Years old:1.01E-02(7.32E-02) (47.1,84.7] Years old:8.38E-01(9.36E-01) Escitalopram:6.08E-02(1.91E-01) Citalopram:1.32E-02(8.63E-02) |
|  | **lysoPC a C24:0** | All Participants:0.08(0.03) Females:0.06(0.04) Males:0.12(0.05) [19.3,31.5] Years old:0.03(0.05) (31.5,47.1] Years old:0.16(0.05) (47.1,84.7] Years old:0.07(0.05) Escitalopram:0.08(0.04) Citalopram:0.10(0.06) | All Participants:5.78E-03(2.25E-02) Females:7.96E-02(2.52E-01) Males:2.90E-02(1.18E-01) [19.3,31.5] Years old:6.06E-01(8.45E-01) (31.5,47.1] Years old:4.35E-03(5.91E-02) (47.1,84.7] Years old:2.00E-01(5.93E-01) Escitalopram:3.64E-02(1.38E-01) Citalopram:6.81E-02(1.68E-01) |
|  | lysoPC a C28:0 | All Participants:0.08(0.03) Females:0.05(0.04) Males:0.13(0.05) [19.3,31.5] Years old:0.08(0.05) (31.5,47.1] Years old:0.11(0.06) (47.1,84.7] Years old:0.04(0.05) Escitalopram:0.07(0.04) Citalopram:0.09(0.06) | All Participants:1.48E-02(5.03E-02) Females:2.12E-01(5.20E-01) Males:2.02E-02(9.96E-02) [19.3,31.5] Years old:1.30E-01(4.94E-01) (31.5,47.1] Years old:8.44E-02(1.70E-01) (47.1,84.7] Years old:3.94E-01(7.47E-01) Escitalopram:6.45E-02(1.95E-01) Citalopram:1.12E-01(2.31E-01) |
|  | lysoPC a C26:0 | All Participants:0.06(0.03) Females:0.05(0.04) Males:0.07(0.06) [19.3,31.5] Years old:0.03(0.06) (31.5,47.1] Years old:0.13(0.07) (47.1,84.7] Years old:0.01(0.05) Escitalopram:0.06(0.04) Citalopram:0.06(0.06) | All Participants:8.67E-02(1.75E-01) Females:2.33E-01(5.43E-01) Males:2.16E-01(3.29E-01) [19.3,31.5] Years old:5.63E-01(8.40E-01) (31.5,47.1] Years old:5.63E-02(1.45E-01) (47.1,84.7] Years old:7.91E-01(9.05E-01) Escitalopram:1.65E-01(3.53E-01) Citalopram:3.21E-01(4.32E-01) |
|  | lysoPC a C18:2 | All Participants:0.07(0.05) Females:0.06(0.06) Males:0.11(0.08) [19.3,31.5] Years old:0.10(0.08) (31.5,47.1] Years old:0.08(0.08) (47.1,84.7] Years old:0.05(0.08) Escitalopram:0.03(0.05) Citalopram:0.17(0.09) | All Participants:1.07E-01(2.00E-01) Females:3.32E-01(6.51E-01) Males:1.69E-01(2.87E-01) [19.3,31.5] Years old:2.32E-01(6.42E-01) (31.5,47.1] Years old:3.53E-01(4.63E-01) (47.1,84.7] Years old:5.34E-01(8.27E-01) Escitalopram:5.20E-01(7.18E-01) Citalopram:6.31E-02(1.68E-01) |
|  | lysoPC a C26:1 | All Participants:0.04(0.03) Females:0.02(0.04) Males:0.07(0.05) [19.3,31.5] Years old:0.05(0.05) (31.5,47.1] Years old:0.09(0.06) (47.1,84.7] Years old:-0.04(0.05) Escitalopram:0.01(0.04) Citalopram:0.09(0.06) | All Participants:2.44E-01(3.52E-01) Females:6.65E-01(8.03E-01) Males:1.90E-01(3.04E-01) [19.3,31.5] Years old:3.09E-01(7.00E-01) (31.5,47.1] Years old:1.12E-01(1.98E-01) (47.1,84.7] Years old:3.45E-01(7.37E-01) Escitalopram:7.13E-01(8.44E-01) Citalopram:1.32E-01(2.48E-01) |
|  | lysoPC a C20:3 | All Participants:0.04(0.05) Females:0.01(0.06) Males:0.09(0.08) [19.3,31.5] Years old:0.02(0.08) (31.5,47.1] Years old:0.06(0.08) (47.1,84.7] Years old:0.05(0.07) Escitalopram:0.01(0.05) Citalopram:0.12(0.09) | All Participants:3.69E-01(4.93E-01) Females:8.26E-01(8.98E-01) Males:2.32E-01(3.41E-01) [19.3,31.5] Years old:8.06E-01(8.87E-01) (31.5,47.1] Years old:4.82E-01(5.70E-01) (47.1,84.7] Years old:5.28E-01(8.27E-01) Escitalopram:8.88E-01(9.46E-01) Citalopram:1.84E-01(3.06E-01) |
|  | lysoPC a C18:0 | All Participants:0.04(0.04) Females:0.04(0.05) Males:0.03(0.07) [19.3,31.5] Years old:0.01(0.07) (31.5,47.1] Years old:0.02(0.08) (47.1,84.7] Years old:0.07(0.07) Escitalopram:0.02(0.06) Citalopram:0.07(0.07) | All Participants:4.22E-01(5.37E-01) Females:4.97E-01(7.43E-01) Males:6.70E-01(7.32E-01) [19.3,31.5] Years old:8.50E-01(9.09E-01) (31.5,47.1] Years old:7.82E-01(8.17E-01) (47.1,84.7] Years old:3.34E-01(7.26E-01) Escitalopram:7.20E-01(8.44E-01) Citalopram:2.89E-01(4.02E-01) |
|  | lysoPC a C16:0 | All Participants:-0.02(0.04) Females:-0.02(0.05) Males:-0.02(0.06) [19.3,31.5] Years old:-0.02(0.06) (31.5,47.1] Years old:-0.05(0.07) (47.1,84.7] Years old:-0.00(0.07) Escitalopram:-0.05(0.05) Citalopram:0.04(0.06) | All Participants:5.31E-01(6.19E-01) Females:6.20E-01(7.80E-01) Males:7.05E-01(7.66E-01) [19.3,31.5] Years old:7.40E-01(8.62E-01) (31.5,47.1] Years old:4.76E-01(5.70E-01) (47.1,84.7] Years old:9.88E-01(9.88E-01) Escitalopram:2.88E-01(4.98E-01) Citalopram:5.10E-01(5.90E-01) |
|  | lysoPC a C17:0 | All Participants:-0.02(0.04) Females:-0.02(0.05) Males:-0.02(0.07) [19.3,31.5] Years old:-0.05(0.07) (31.5,47.1] Years old:-0.00(0.07) (47.1,84.7] Years old:-0.02(0.07) Escitalopram:-0.06(0.05) Citalopram:0.06(0.07) | All Participants:5.64E-01(6.42E-01) Females:6.09E-01(7.80E-01) Males:7.81E-01(8.11E-01) [19.3,31.5] Years old:5.06E-01(8.25E-01) (31.5,47.1] Years old:9.97E-01(9.97E-01) (47.1,84.7] Years old:7.71E-01(9.03E-01) Escitalopram:2.39E-01(4.49E-01) Citalopram:3.71E-01(4.82E-01) |
|  | lysoPC a C16:1 | All Participants:0.01(0.04) Females:-0.01(0.05) Males:0.04(0.07) [19.3,31.5] Years old:-0.04(0.07) (31.5,47.1] Years old:0.03(0.06) (47.1,84.7] Years old:0.03(0.07) Escitalopram:-0.01(0.05) Citalopram:0.06(0.07) | All Participants:8.42E-01(9.03E-01) Females:8.48E-01(9.09E-01) Males:5.59E-01(6.43E-01) [19.3,31.5] Years old:6.01E-01(8.45E-01) (31.5,47.1] Years old:6.23E-01(7.00E-01) (47.1,84.7] Years old:6.33E-01(8.51E-01) Escitalopram:7.94E-01(8.93E-01) Citalopram:4.26E-01(5.34E-01) |
|  | lysoPC a C18:1 | All Participants:0.00(0.04) Females:-0.02(0.05) Males:0.05(0.06) [19.3,31.5] Years old:-0.05(0.07) (31.5,47.1] Years old:0.02(0.06) (47.1,84.7] Years old:0.05(0.07) Escitalopram:-0.03(0.05) Citalopram:0.09(0.07) | All Participants:9.04E-01(9.33E-01) Females:6.84E-01(8.14E-01) Males:4.33E-01(5.35E-01) [19.3,31.5] Years old:4.83E-01(8.25E-01) (31.5,47.1] Years old:7.38E-01(7.76E-01) (47.1,84.7] Years old:5.03E-01(8.21E-01) Escitalopram:4.73E-01(6.75E-01) Citalopram:2.08E-01(3.20E-01) |

| Class | Metabolite | Estimated Log2 Change (SE) | p-value(q-value) |
| --- | --- | --- | --- |
| Acylcarnitine | C18:1 | All Participants:-0.38(0.06) Females:-0.36(0.08) Males:-0.43(0.11) [19.3,31.5] Years old:-0.40(0.10) (31.5,47.1] Years old:-0.36(0.12) (47.1,84.7] Years old:-0.39(0.11) | All Participants:6.67E-09(4.98E-07) Females:6.05E-06(3.29E-04) Males:2.25E-04(7.34E-03) [19.3,31.5] Years old:2.21E-04(1.20E-02) (31.5,47.1] Years old:3.58E-03(5.30E-02) (47.1,84.7] Years old:6.93E-04(3.35E-02) |
|  | C16:1 | All Participants:-0.31(0.05) Females:-0.32(0.07) Males:-0.30(0.09) [19.3,31.5] Years old:-0.38(0.09) (31.5,47.1] Years old:-0.24(0.09) (47.1,84.7] Years old:-0.32(0.09) | All Participants:9.17E-09(4.98E-07) Females:3.74E-06(3.05E-04) Males:8.36E-04(1.51E-02) [19.3,31.5] Years old:7.51E-05(6.12E-03) (31.5,47.1] Years old:8.64E-03(7.32E-02) (47.1,84.7] Years old:1.03E-03(3.35E-02) |
|  | C18:2 | All Participants:-0.38(0.07) Females:-0.35(0.08) Males:-0.44(0.12) [19.3,31.5] Years old:-0.35(0.11) (31.5,47.1] Years old:-0.38(0.13) (47.1,84.7] Years old:-0.42(0.11) | All Participants:2.51E-08(1.02E-06) Females:1.94E-05(6.31E-04) Males:3.40E-04(9.25E-03) [19.3,31.5] Years old:1.74E-03(3.10E-02) (31.5,47.1] Years old:5.33E-03(6.08E-02) (47.1,84.7] Years old:1.90E-04(1.55E-02) |
|  | **C14:2** | All Participants:-0.36(0.08) Females:-0.46(0.10) Males:-0.20(0.12) [19.3,31.5] Years old:-0.41(0.13) (31.5,47.1] Years old:-0.20(0.14) (47.1,84.7] Years old:-0.48(0.14) | All Participants:4.58E-06(1.23E-04) Females:1.33E-05(5.40E-04) Males:9.38E-02(2.12E-01) [19.3,31.5] Years old:1.90E-03(3.10E-02) (31.5,47.1] Years old:1.47E-01(2.34E-01) (47.1,84.7] Years old:9.07E-04(3.35E-02) |
|  | **C16** | All Participants:-0.21(0.05) Females:-0.19(0.05) Males:-0.24(0.09) [19.3,31.5] Years old:-0.27(0.07) (31.5,47.1] Years old:-0.15(0.08) (47.1,84.7] Years old:-0.21(0.08) | All Participants:5.28E-06(1.23E-04) Females:2.59E-04(4.70E-03) Males:6.40E-03(4.97E-02) [19.3,31.5] Years old:4.69E-04(1.30E-02) (31.5,47.1] Years old:8.01E-02(1.68E-01) (47.1,84.7] Years old:8.07E-03(1.20E-01) |
|  | **C10** | All Participants:-0.33(0.08) Females:-0.40(0.10) Males:-0.21(0.11) [19.3,31.5] Years old:-0.49(0.13) (31.5,47.1] Years old:-0.14(0.13) (47.1,84.7] Years old:-0.35(0.14) | All Participants:2.91E-05(5.93E-04) Females:1.75E-04(3.56E-03) Males:6.63E-02(1.64E-01) [19.3,31.5] Years old:3.68E-04(1.30E-02) (31.5,47.1] Years old:2.85E-01(4.03E-01) (47.1,84.7] Years old:1.30E-02(1.43E-01) |
|  | **C12** | All Participants:-0.32(0.08) Females:-0.40(0.10) Males:-0.18(0.13) [19.3,31.5] Years old:-0.48(0.14) (31.5,47.1] Years old:-0.11(0.13) (47.1,84.7] Years old:-0.36(0.15) | All Participants:8.86E-05(1.55E-03) Females:1.26E-04(2.94E-03) Males:1.71E-01(2.88E-01) [19.3,31.5] Years old:6.39E-04(1.30E-02) (31.5,47.1] Years old:3.97E-01(4.94E-01) (47.1,84.7] Years old:1.67E-02(1.60E-01) |
|  | **C8** | All Participants:-0.31(0.08) Females:-0.41(0.10) Males:-0.12(0.12) [19.3,31.5] Years old:-0.49(0.14) (31.5,47.1] Years old:-0.12(0.14) (47.1,84.7] Years old:-0.31(0.13) | All Participants:1.10E-04(1.64E-03) Females:8.22E-05(2.23E-03) Males:3.17E-01(4.49E-01) [19.3,31.5] Years old:5.58E-04(1.30E-02) (31.5,47.1] Years old:4.03E-01(4.95E-01) (47.1,84.7] Years old:1.97E-02(1.68E-01) |
|  | **C5-OH (C3-DC-M)** | All Participants:-0.24(0.06) Females:-0.23(0.08) Males:-0.25(0.11) [19.3,31.5] Years old:-0.18(0.12) (31.5,47.1] Years old:-0.20(0.11) (47.1,84.7] Years old:-0.35(0.10) | All Participants:2.24E-04(3.04E-03) Females:2.74E-03(2.58E-02) Males:2.68E-02(1.12E-01) [19.3,31.5] Years old:1.30E-01(4.94E-01) (31.5,47.1] Years old:8.55E-02(1.70E-01) (47.1,84.7] Years old:1.39E-03(3.78E-02) |
|  | **C3** | All Participants:0.16(0.04) Females:0.09(0.06) Males:0.27(0.06) [19.3,31.5] Years old:0.15(0.07) (31.5,47.1] Years old:0.28(0.08) (47.1,84.7] Years old:0.04(0.08) | All Participants:3.24E-04(3.61E-03) Females:1.03E-01(3.11E-01) Males:4.64E-05(3.78E-03) [19.3,31.5] Years old:2.56E-02(2.01E-01) (31.5,47.1] Years old:4.87E-04(1.95E-02) (47.1,84.7] Years old:5.98E-01(8.42E-01) |
|  | **C14:1-OH** | All Participants:-0.14(0.04) Females:-0.15(0.05) Males:-0.11(0.07) [19.3,31.5] Years old:-0.21(0.07) (31.5,47.1] Years old:-0.02(0.06) (47.1,84.7] Years old:-0.18(0.06) | All Participants:4.09E-04(3.79E-03) Females:1.21E-03(1.79E-02) Males:1.05E-01(2.26E-01) [19.3,31.5] Years old:3.61E-03(5.35E-02) (31.5,47.1] Years old:7.34E-01(7.76E-01) (47.1,84.7] Years old:6.77E-03(1.20E-01) |
|  | **C7-DC** | All Participants:-0.17(0.05) Females:-0.18(0.06) Males:-0.17(0.09) [19.3,31.5] Years old:-0.24(0.08) (31.5,47.1] Years old:-0.04(0.08) (47.1,84.7] Years old:-0.24(0.10) | All Participants:5.08E-04(4.14E-03) Females:3.85E-03(3.30E-02) Males:5.44E-02(1.54E-01) [19.3,31.5] Years old:4.87E-03(6.11E-02) (31.5,47.1] Years old:6.47E-01(7.06E-01) (47.1,84.7] Years old:1.19E-02(1.43E-01) |
|  | **C5** | All Participants:0.13(0.04) Females:0.06(0.06) Males:0.27(0.07) [19.3,31.5] Years old:0.14(0.07) (31.5,47.1] Years old:0.30(0.08) (47.1,84.7] Years old:-0.04(0.08) | All Participants:2.47E-03(1.34E-02) Females:2.86E-01(6.06E-01) Males:2.10E-04(7.34E-03) [19.3,31.5] Years old:5.23E-02(2.94E-01) (31.5,47.1] Years old:1.72E-04(1.36E-02) (47.1,84.7] Years old:5.85E-01(8.42E-01) |
|  | **C2** | All Participants:-0.16(0.06) Females:-0.23(0.07) Males:-0.04(0.08) [19.3,31.5] Years old:-0.25(0.09) (31.5,47.1] Years old:-0.09(0.09) (47.1,84.7] Years old:-0.15(0.10) | All Participants:3.47E-03(1.71E-02) Females:2.17E-03(2.21E-02) Males:5.86E-01(6.59E-01) [19.3,31.5] Years old:9.19E-03(9.99E-02) (31.5,47.1] Years old:3.10E-01(4.21E-01) (47.1,84.7] Years old:1.61E-01(5.72E-01) |
|  | **C16:1-OH** | All Participants:-0.13(0.05) Females:-0.16(0.05) Males:-0.08(0.09) [19.3,31.5] Years old:-0.21(0.09) (31.5,47.1] Years old:-0.01(0.07) (47.1,84.7] Years old:-0.16(0.07) | All Participants:4.66E-03(2.00E-02) Females:1.82E-03(2.21E-02) Males:3.93E-01(5.07E-01) [19.3,31.5] Years old:2.73E-02(2.01E-01) (31.5,47.1] Years old:8.48E-01(8.75E-01) (47.1,84.7] Years old:1.77E-02(1.60E-01) |
|  | **C16:2-OH** | All Participants:-0.13(0.05) Females:-0.14(0.05) Males:-0.12(0.09) [19.3,31.5] Years old:-0.25(0.09) (31.5,47.1] Years old:-0.06(0.08) (47.1,84.7] Years old:-0.08(0.07) | All Participants:4.71E-03(2.00E-02) Females:8.77E-03(5.96E-02) Males:1.85E-01(3.02E-01) [19.3,31.5] Years old:4.27E-03(5.80E-02) (31.5,47.1] Years old:4.80E-01(5.70E-01) (47.1,84.7] Years old:2.62E-01(6.81E-01) |
|  | **C18** | All Participants:-0.11(0.04) Females:-0.10(0.05) Males:-0.12(0.06) [19.3,31.5] Years old:-0.12(0.07) (31.5,47.1] Years old:-0.07(0.07) (47.1,84.7] Years old:-0.12(0.06) | All Participants:4.96E-03(2.00E-02) Females:3.79E-02(1.67E-01) Males:5.81E-02(1.54E-01) [19.3,31.5] Years old:5.99E-02(3.14E-01) (31.5,47.1] Years old:2.82E-01(4.03E-01) (47.1,84.7] Years old:6.01E-02(2.88E-01) |
|  | **C18:1-OH** | All Participants:-0.14(0.05) Females:-0.19(0.06) Males:-0.05(0.09) [19.3,31.5] Years old:-0.22(0.10) (31.5,47.1] Years old:-0.02(0.07) (47.1,84.7] Years old:-0.17(0.08) | All Participants:4.97E-03(2.00E-02) Females:1.50E-03(2.04E-02) Males:5.86E-01(6.59E-01) [19.3,31.5] Years old:2.96E-02(2.01E-01) (31.5,47.1] Years old:8.06E-01(8.37E-01) (47.1,84.7] Years old:3.09E-02(2.06E-01) |
|  | **C14:1** | All Participants:-0.10(0.04) Females:-0.14(0.04) Males:-0.04(0.06) [19.3,31.5] Years old:-0.14(0.06) (31.5,47.1] Years old:-0.01(0.06) (47.1,84.7] Years old:-0.16(0.07) | All Participants:5.03E-03(2.00E-02) Females:2.05E-03(2.21E-02) Males:5.37E-01(6.25E-01) [19.3,31.5] Years old:2.07E-02(1.78E-01) (31.5,47.1] Years old:9.10E-01(9.27E-01) (47.1,84.7] Years old:2.17E-02(1.68E-01) |
|  | **C4** | All Participants:0.09(0.04) Females:0.03(0.05) Males:0.21(0.05) [19.3,31.5] Years old:0.06(0.07) (31.5,47.1] Years old:0.18(0.06) (47.1,84.7] Years old:0.05(0.06) | All Participants:9.16E-03(3.39E-02) Females:5.06E-01(7.48E-01) Males:1.70E-04(7.34E-03) [19.3,31.5] Years old:4.05E-01(7.84E-01) (31.5,47.1] Years old:2.93E-03(5.30E-02) (47.1,84.7] Years old:4.25E-01(7.93E-01) |
|  | **C0** | All Participants:0.06(0.03) Females:0.03(0.04) Males:0.12(0.04) [19.3,31.5] Years old:0.07(0.05) (31.5,47.1] Years old:0.08(0.05) (47.1,84.7] Years old:0.03(0.04) | All Participants:2.75E-02(8.00E-02) Females:4.11E-01(6.79E-01) Males:4.57E-03(4.66E-02) [19.3,31.5] Years old:1.47E-01(5.09E-01) (31.5,47.1] Years old:1.19E-01(2.04E-01) (47.1,84.7] Years old:4.94E-01(8.21E-01) |
|  | **C5-M-DC** | All Participants:0.14(0.06) Females:0.03(0.08) Males:0.32(0.11) [19.3,31.5] Years old:0.00(0.10) (31.5,47.1] Years old:0.34(0.12) (47.1,84.7] Years old:0.07(0.10) | All Participants:3.09E-02(8.85E-02) Females:6.61E-01(8.03E-01) Males:4.10E-03(4.66E-02) [19.3,31.5] Years old:9.79E-01(9.91E-01) (31.5,47.1] Years old:5.59E-03(6.08E-02) (47.1,84.7] Years old:5.24E-01(8.27E-01) |
| Amino Acids | **Arginine** | All Participants:0.29(0.09) Females:0.22(0.10) Males:0.43(0.16) [19.3,31.5] Years old:0.15(0.12) (31.5,47.1] Years old:0.54(0.18) (47.1,84.7] Years old:0.19(0.14) | All Participants:7.11E-04(5.46E-03) Females:2.98E-02(1.52E-01) Males:8.12E-03(5.09E-02) [19.3,31.5] Years old:2.15E-01(6.14E-01) (31.5,47.1] Years old:3.18E-03(5.30E-02) (47.1,84.7] Years old:1.80E-01(5.93E-01) |
|  | **Proline** | All Participants:0.13(0.04) Females:0.09(0.06) Males:0.20(0.06) [19.3,31.5] Years old:0.14(0.07) (31.5,47.1] Years old:0.16(0.08) (47.1,84.7] Years old:0.08(0.08) | All Participants:3.41E-03(1.71E-02) Females:1.24E-01(3.62E-01) Males:2.73E-03(3.71E-02) [19.3,31.5] Years old:5.62E-02(3.05E-01) (31.5,47.1] Years old:3.38E-02(1.11E-01) (47.1,84.7] Years old:3.10E-01(7.02E-01) |
|  | **Tyrosine** | All Participants:0.10(0.04) Females:0.03(0.05) Males:0.21(0.06) [19.3,31.5] Years old:0.09(0.06) (31.5,47.1] Years old:0.20(0.07) (47.1,84.7] Years old:0.01(0.07) | All Participants:1.54E-02(5.08E-02) Females:5.15E-01(7.48E-01) Males:1.01E-03(1.64E-02) [19.3,31.5] Years old:1.62E-01(5.21E-01) (31.5,47.1] Years old:1.09E-02(7.38E-02) (47.1,84.7] Years old:9.01E-01(9.52E-01) |
|  | **Citrulline** | All Participants:0.09(0.04) Females:0.07(0.05) Males:0.14(0.07) [19.3,31.5] Years old:0.09(0.06) (31.5,47.1] Years old:0.23(0.07) (47.1,84.7] Years old:-0.04(0.08) | All Participants:1.84E-02(5.77E-02) Females:1.63E-01(4.28E-01) Males:4.09E-02(1.31E-01) [19.3,31.5] Years old:1.34E-01(4.98E-01) (31.5,47.1] Years old:1.46E-03(3.97E-02) (47.1,84.7] Years old:6.42E-01(8.51E-01) |
|  | **Phenylalanine** | All Participants:0.06(0.03) Females:0.02(0.04) Males:0.14(0.05) [19.3,31.5] Years old:0.06(0.05) (31.5,47.1] Years old:0.11(0.06) (47.1,84.7] Years old:0.02(0.06) | All Participants:4.34E-02(1.12E-01) Females:5.90E-01(7.79E-01) Males:6.00E-03(4.97E-02) [19.3,31.5] Years old:2.32E-01(6.42E-01) (31.5,47.1] Years old:5.86E-02(1.46E-01) (47.1,84.7] Years old:7.25E-01(8.95E-01) |
| Biogenic Amines | **Serotonin** | All Participants:-1.77(0.17) Females:-1.82(0.21) Males:-1.68(0.29) [19.3,31.5] Years old:-1.75(0.29) (31.5,47.1] Years old:-1.81(0.29) (47.1,84.7] Years old:-1.74(0.30) | All Participants:1.55E-21(2.53E-19) Females:2.36E-15(3.84E-13) Males:1.25E-07(2.04E-05) [19.3,31.5] Years old:2.77E-08(4.51E-06) (31.5,47.1] Years old:2.67E-08(4.36E-06) (47.1,84.7] Years old:1.56E-07(2.55E-05) |
| Biogenic Amines | **Sarcosine** | All Participants:-0.34(0.09) Females:-0.27(0.11) Males:-0.47(0.13) [19.3,31.5] Years old:-0.16(0.15) (31.5,47.1] Years old:-0.41(0.15) (47.1,84.7] Years old:-0.47(0.15) | All Participants:9.53E-05(1.55E-03) Females:1.52E-02(9.19E-02) Males:4.70E-04(9.57E-03) [19.3,31.5] Years old:3.08E-01(7.00E-01) (31.5,47.1] Years old:9.42E-03(7.32E-02) (47.1,84.7] Years old:2.06E-03(4.81E-02) |
|  | **Methioninesulfoxide** | All Participants:0.31(0.08) Females:0.33(0.10) Males:0.27(0.14) [19.3,31.5] Years old:0.49(0.14) (31.5,47.1] Years old:0.35(0.14) (47.1,84.7] Years old:0.07(0.16) | All Participants:3.34E-04(3.61E-03) Females:2.10E-03(2.21E-02) Males:6.22E-02(1.56E-01) [19.3,31.5] Years old:6.36E-04(1.30E-02) (31.5,47.1] Years old:1.33E-02(8.01E-02) (47.1,84.7] Years old:6.67E-01(8.70E-01) |
|  | **trans-4-Hydroxyproline** | All Participants:0.15(0.07) Females:0.08(0.08) Males:0.29(0.12) [19.3,31.5] Years old:0.15(0.11) (31.5,47.1] Years old:0.34(0.13) (47.1,84.7] Years old:-0.03(0.12) | All Participants:2.50E-02(7.53E-02) Females:3.39E-01(6.55E-01) Males:1.60E-02(8.97E-02) [19.3,31.5] Years old:1.55E-01(5.21E-01) (31.5,47.1] Years old:8.43E-03(7.32E-02) (47.1,84.7] Years old:8.05E-01(9.11E-01) |
| PC aa | **PC aa C36:1** | All Participants:0.15(0.04) Females:0.12(0.05) Males:0.20(0.07) [19.3,31.5] Years old:0.09(0.07) (31.5,47.1] Years old:0.21(0.08) (47.1,84.7] Years old:0.14(0.06) | All Participants:4.26E-04(3.79E-03) Females:1.82E-02(1.06E-01) Males:8.09E-03(5.09E-02) [19.3,31.5] Years old:1.74E-01(5.28E-01) (31.5,47.1] Years old:1.71E-02(8.20E-02) (47.1,84.7] Years old:2.09E-02(1.68E-01) |
|  | **PC aa C30:0** | All Participants:0.17(0.05) Females:0.12(0.06) Males:0.24(0.08) [19.3,31.5] Years old:0.17(0.08) (31.5,47.1] Years old:0.23(0.10) (47.1,84.7] Years old:0.10(0.07) | All Participants:4.41E-04(3.79E-03) Females:3.51E-02(1.59E-01) Males:2.59E-03(3.71E-02) [19.3,31.5] Years old:3.06E-02(2.01E-01) (31.5,47.1] Years old:2.11E-02(8.82E-02) (47.1,84.7] Years old:1.45E-01(5.53E-01) |
|  | **PC aa C42:2** | All Participants:0.09(0.03) Females:0.07(0.03) Males:0.13(0.05) [19.3,31.5] Years old:0.04(0.04) (31.5,47.1] Years old:0.19(0.05) (47.1,84.7] Years old:0.05(0.05) | All Participants:7.54E-04(5.46E-03) Females:2.65E-02(1.44E-01) Males:9.61E-03(5.80E-02) [19.3,31.5] Years old:4.14E-01(7.84E-01) (31.5,47.1] Years old:2.50E-04(1.36E-02) (47.1,84.7] Years old:2.63E-01(6.81E-01) |
|  | **PC aa C34:4** | All Participants:0.16(0.05) Females:0.12(0.06) Males:0.24(0.08) [19.3,31.5] Years old:0.21(0.09) (31.5,47.1] Years old:0.24(0.09) (47.1,84.7] Years old:0.03(0.07) | All Participants:7.70E-04(5.46E-03) Females:4.29E-02(1.74E-01) Males:4.55E-03(4.66E-02) [19.3,31.5] Years old:1.66E-02(1.60E-01) (31.5,47.1] Years old:1.03E-02(7.32E-02) (47.1,84.7] Years old:6.12E-01(8.51E-01) |
|  | **PC aa C34:3** | All Participants:0.13(0.04) Females:0.09(0.05) Males:0.22(0.08) [19.3,31.5] Years old:0.12(0.07) (31.5,47.1] Years old:0.21(0.08) (47.1,84.7] Years old:0.07(0.06) | All Participants:1.00E-03(6.12E-03) Females:6.72E-02(2.28E-01) Males:4.37E-03(4.66E-02) [19.3,31.5] Years old:6.44E-02(3.14E-01) (31.5,47.1] Years old:1.42E-02(8.01E-02) (47.1,84.7] Years old:2.39E-01(6.41E-01) |
|  | **PC aa C40:2** | All Participants:0.10(0.03) Females:0.07(0.03) Males:0.15(0.05) [19.3,31.5] Years old:0.05(0.05) (31.5,47.1] Years old:0.16(0.05) (47.1,84.7] Years old:0.08(0.05) | All Participants:1.00E-03(6.12E-03) Females:4.48E-02(1.74E-01) Males:7.56E-03(5.09E-02) [19.3,31.5] Years old:3.31E-01(7.29E-01) (31.5,47.1] Years old:1.95E-03(4.54E-02) (47.1,84.7] Years old:1.15E-01(4.79E-01) |
|  | **PC aa C38:6** | All Participants:-0.08(0.03) Females:-0.10(0.03) Males:-0.04(0.05) [19.3,31.5] Years old:-0.10(0.05) (31.5,47.1] Years old:-0.03(0.06) (47.1,84.7] Years old:-0.11(0.04) | All Participants:3.84E-03(1.84E-02) Females:2.85E-03(2.58E-02) Males:3.89E-01(5.07E-01) [19.3,31.5] Years old:3.08E-02(2.01E-01) (31.5,47.1] Years old:6.50E-01(7.06E-01) (47.1,84.7] Years old:6.08E-03(1.20E-01) |
|  | **PC aa C24:0** | All Participants:0.12(0.04) Females:0.09(0.05) Males:0.17(0.07) [19.3,31.5] Years old:0.08(0.07) (31.5,47.1] Years old:0.26(0.09) (47.1,84.7] Years old:0.02(0.06) | All Participants:4.93E-03(2.00E-02) Females:8.03E-02(2.52E-01) Males:1.81E-02(9.52E-02) [19.3,31.5] Years old:2.39E-01(6.49E-01) (31.5,47.1] Years old:3.52E-03(5.30E-02) (47.1,84.7] Years old:7.66E-01(9.03E-01) |
|  | **PC aa C28:1** | All Participants:0.08(0.03) Females:0.04(0.03) Males:0.15(0.05) [19.3,31.5] Years old:0.08(0.05) (31.5,47.1] Years old:0.12(0.06) (47.1,84.7] Years old:0.03(0.05) | All Participants:6.90E-03(2.62E-02) Females:2.49E-01(5.57E-01) Males:6.18E-03(4.97E-02) [19.3,31.5] Years old:7.78E-02(3.52E-01) (31.5,47.1] Years old:4.28E-02(1.27E-01) (47.1,84.7] Years old:4.77E-01(8.20E-01) |
|  | **PC aa C36:6** | All Participants:0.11(0.04) Females:0.08(0.05) Males:0.18(0.07) [19.3,31.5] Years old:0.12(0.08) (31.5,47.1] Years old:0.20(0.09) (47.1,84.7] Years old:0.02(0.06) | All Participants:9.60E-03(3.48E-02) Females:1.50E-01(4.07E-01) Males:1.80E-02(9.52E-02) [19.3,31.5] Years old:1.07E-01(4.27E-01) (31.5,47.1] Years old:2.07E-02(8.82E-02) (47.1,84.7] Years old:8.14E-01(9.15E-01) |
|  | **PC aa C42:4** | All Participants:0.07(0.03) Females:0.05(0.03) Males:0.11(0.05) [19.3,31.5] Years old:0.04(0.05) (31.5,47.1] Years old:0.13(0.05) (47.1,84.7] Years old:0.05(0.04) | All Participants:1.11E-02(3.92E-02) Females:1.35E-01(3.73E-01) Males:3.10E-02(1.20E-01) [19.3,31.5] Years old:4.31E-01(7.98E-01) (31.5,47.1] Years old:1.62E-02(8.01E-02) (47.1,84.7] Years old:2.94E-01(6.93E-01) |
|  | **PC aa C36:3** | All Participants:0.04(0.02) Females:0.03(0.02) Males:0.07(0.03) [19.3,31.5] Years old:0.04(0.03) (31.5,47.1] Years old:0.08(0.03) (47.1,84.7] Years old:0.02(0.02) | All Participants:1.56E-02(5.08E-02) Females:1.91E-01(4.83E-01) Males:2.49E-02(1.09E-01) [19.3,31.5] Years old:2.53E-01(6.66E-01) (31.5,47.1] Years old:2.60E-02(1.01E-01) (47.1,84.7] Years old:5.33E-01(8.27E-01) |
|  | **PC aa C32:1** | All Participants:0.13(0.06) Females:0.09(0.07) Males:0.22(0.10) [19.3,31.5] Years old:0.06(0.10) (31.5,47.1] Years old:0.22(0.12) (47.1,84.7] Years old:0.12(0.09) | All Participants:2.28E-02(7.02E-02) Females:2.22E-01(5.24E-01) Males:3.86E-02(1.27E-01) [19.3,31.5] Years old:5.16E-01(8.30E-01) (31.5,47.1] Years old:6.35E-02(1.50E-01) (47.1,84.7] Years old:1.94E-01(5.93E-01) |
|  | **PC aa C36:2** | All Participants:0.03(0.02) Females:0.02(0.02) Males:0.06(0.03) [19.3,31.5] Years old:0.02(0.03) (31.5,47.1] Years old:0.06(0.03) (47.1,84.7] Years old:0.01(0.02) | All Participants:3.40E-02(9.55E-02) Females:3.90E-01(6.79E-01) Males:2.19E-02(1.02E-01) [19.3,31.5] Years old:3.94E-01(7.84E-01) (31.5,47.1] Years old:3.37E-02(1.11E-01) (47.1,84.7] Years old:5.56E-01(8.31E-01) |
|  | **PC aa C38:3** | All Participants:0.08(0.04) Females:0.06(0.05) Males:0.13(0.07) [19.3,31.5] Years old:0.05(0.07) (31.5,47.1] Years old:0.14(0.08) (47.1,84.7] Years old:0.06(0.06) | All Participants:3.97E-02(1.04E-01) Females:2.44E-01(5.57E-01) Males:7.45E-02(1.81E-01) [19.3,31.5] Years old:4.73E-01(8.21E-01) (31.5,47.1] Years old:8.02E-02(1.68E-01) (47.1,84.7] Years old:3.26E-01(7.19E-01) |
|  | **PC aa C36:5** | All Participants:0.09(0.04) Females:0.05(0.05) Males:0.16(0.08) [19.3,31.5] Years old:0.12(0.07) (31.5,47.1] Years old:0.17(0.08) (47.1,84.7] Years old:-0.03(0.07) | All Participants:4.70E-02(1.19E-01) Females:3.82E-01(6.79E-01) Males:3.91E-02(1.27E-01) [19.3,31.5] Years old:8.69E-02(3.83E-01) (31.5,47.1] Years old:4.44E-02(1.29E-01) (47.1,84.7] Years old:6.91E-01(8.83E-01) |
| PC ae | **PC ae C34:3** | All Participants:0.17(0.03) Females:0.14(0.04) Males:0.21(0.06) [19.3,31.5] Years old:0.16(0.06) (31.5,47.1] Years old:0.23(0.06) (47.1,84.7] Years old:0.11(0.05) | All Participants:1.29E-06(4.21E-05) Females:7.89E-04(1.29E-02) Males:3.98E-04(9.28E-03) [19.3,31.5] Years old:8.38E-03(9.75E-02) (31.5,47.1] Years old:5.99E-04(1.95E-02) (47.1,84.7] Years old:3.42E-02(2.06E-01) |
|  | **PC ae C38:2** | All Participants:0.13(0.04) Females:0.12(0.04) Males:0.15(0.06) [19.3,31.5] Years old:0.11(0.06) (31.5,47.1] Years old:0.19(0.07) (47.1,84.7] Years old:0.09(0.05) | All Participants:3.46E-04(3.61E-03) Females:7.12E-03(5.05E-02) Males:1.98E-02(9.96E-02) [19.3,31.5] Years old:7.37E-02(3.43E-01) (31.5,47.1] Years old:9.70E-03(7.32E-02) (47.1,84.7] Years old:9.91E-02(4.49E-01) |
|  | **PC ae C36:3** | All Participants:0.12(0.03) Females:0.10(0.04) Males:0.16(0.06) [19.3,31.5] Years old:0.11(0.06) (31.5,47.1] Years old:0.18(0.06) (47.1,84.7] Years old:0.07(0.05) | All Participants:3.55E-04(3.61E-03) Females:1.88E-02(1.06E-01) Males:5.65E-03(4.97E-02) [19.3,31.5] Years old:6.44E-02(3.14E-01) (31.5,47.1] Years old:6.73E-03(6.85E-02) (47.1,84.7] Years old:1.54E-01(5.57E-01) |
|  | **PC ae C34:2** | All Participants:0.11(0.03) Females:0.09(0.04) Males:0.14(0.05) [19.3,31.5] Years old:0.12(0.06) (31.5,47.1] Years old:0.17(0.06) (47.1,84.7] Years old:0.04(0.05) | All Participants:8.88E-04(6.03E-03) Females:3.08E-02(1.52E-01) Males:8.07E-03(5.09E-02) [19.3,31.5] Years old:4.34E-02(2.62E-01) (31.5,47.1] Years old:9.17E-03(7.32E-02) (47.1,84.7] Years old:3.91E-01(7.47E-01) |
|  | **PC ae C30:0** | All Participants:0.11(0.03) Females:0.09(0.04) Males:0.16(0.06) [19.3,31.5] Years old:0.14(0.06) (31.5,47.1] Years old:0.15(0.07) (47.1,84.7] Years old:0.05(0.05) | All Participants:1.01E-03(6.12E-03) Females:4.04E-02(1.73E-01) Males:7.18E-03(5.09E-02) [19.3,31.5] Years old:1.60E-02(1.60E-01) (31.5,47.1] Years old:3.16E-02(1.11E-01) (47.1,84.7] Years old:3.56E-01(7.37E-01) |
|  | **PC ae C42:2** | All Participants:0.08(0.03) Females:0.05(0.04) Males:0.12(0.05) [19.3,31.5] Years old:0.06(0.05) (31.5,47.1] Years old:0.15(0.06) (47.1,84.7] Years old:0.03(0.05) | All Participants:1.13E-02(3.92E-02) Females:1.57E-01(4.21E-01) Males:2.13E-02(1.02E-01) [19.3,31.5] Years old:2.75E-01(6.79E-01) (31.5,47.1] Years old:1.18E-02(7.71E-02) (47.1,84.7] Years old:5.84E-01(8.42E-01) |
|  | **PC ae C36:2** | All Participants:0.07(0.03) Females:0.05(0.04) Males:0.10(0.05) [19.3,31.5] Years old:0.05(0.05) (31.5,47.1] Years old:0.14(0.06) (47.1,84.7] Years old:0.02(0.04) | All Participants:1.65E-02(5.27E-02) Females:1.30E-01(3.67E-01) Males:5.64E-02(1.54E-01) [19.3,31.5] Years old:2.99E-01(6.95E-01) (31.5,47.1] Years old:1.62E-02(8.01E-02) (47.1,84.7] Years old:7.11E-01(8.92E-01) |
|  | **PC ae C36:5** | All Participants:0.07(0.03) Females:0.05(0.04) Males:0.10(0.05) [19.3,31.5] Years old:0.07(0.05) (31.5,47.1] Years old:0.14(0.06) (47.1,84.7] Years old:-0.01(0.04) | All Participants:2.64E-02(7.84E-02) Females:1.92E-01(4.83E-01) Males:5.80E-02(1.54E-01) [19.3,31.5] Years old:1.63E-01(5.21E-01) (31.5,47.1] Years old:1.55E-02(8.01E-02) (47.1,84.7] Years old:7.47E-01(9.02E-01) |
|  | **PC ae C36:0** | All Participants:0.06(0.03) Females:0.04(0.03) Males:0.08(0.04) [19.3,31.5] Years old:0.02(0.05) (31.5,47.1] Years old:0.12(0.05) (47.1,84.7] Years old:0.04(0.04) | All Participants:3.52E-02(9.65E-02) Females:2.16E-01(5.20E-01) Males:6.03E-02(1.54E-01) [19.3,31.5] Years old:7.22E-01(8.62E-01) (31.5,47.1] Years old:2.33E-02(9.26E-02) (47.1,84.7] Years old:3.93E-01(7.47E-01) |
|  | **PC ae C32:2** | All Participants:0.05(0.03) Females:0.03(0.03) Males:0.09(0.04) [19.3,31.5] Years old:0.02(0.04) (31.5,47.1] Years old:0.14(0.05) (47.1,84.7] Years old:-0.01(0.04) | All Participants:4.74E-02(1.19E-01) Females:4.07E-01(6.79E-01) Males:3.69E-02(1.27E-01) [19.3,31.5] Years old:6.02E-01(8.45E-01) (31.5,47.1] Years old:5.45E-03(6.08E-02) (47.1,84.7] Years old:7.63E-01(9.03E-01) |
| Sphingolipids | **SM C18:1** | All Participants:-0.10(0.03) Females:-0.12(0.04) Males:-0.07(0.05) [19.3,31.5] Years old:-0.11(0.06) (31.5,47.1] Years old:-0.06(0.06) (47.1,84.7] Years old:-0.12(0.05) | All Participants:1.83E-03(1.03E-02) Females:4.23E-03(3.45E-02) Males:1.79E-01(2.98E-01) [19.3,31.5] Years old:4.79E-02(2.79E-01) (31.5,47.1] Years old:2.98E-01(4.09E-01) (47.1,84.7] Years old:1.49E-02(1.52E-01) |
|  | **SM C24:0** | All Participants:0.09(0.03) Females:0.08(0.04) Males:0.11(0.06) [19.3,31.5] Years old:0.06(0.05) (31.5,47.1] Years old:0.14(0.06) (47.1,84.7] Years old:0.07(0.05) | All Participants:4.14E-03(1.93E-02) Females:3.23E-02(1.55E-01) Males:5.95E-02(1.54E-01) [19.3,31.5] Years old:2.76E-01(6.79E-01) (31.5,47.1] Years old:1.41E-02(8.01E-02) (47.1,84.7] Years old:2.00E-01(5.93E-01) |
|  | **SM C18:0** | All Participants:-0.06(0.03) Females:-0.08(0.04) Males:-0.03(0.05) [19.3,31.5] Years old:-0.07(0.05) (31.5,47.1] Years old:-0.03(0.06) (47.1,84.7] Years old:-0.09(0.05) | All Participants:3.55E-02(9.65E-02) Females:3.45E-02(1.59E-01) Males:4.96E-01(5.86E-01) [19.3,31.5] Years old:2.12E-01(6.14E-01) (31.5,47.1] Years old:5.74E-01(6.63E-01) (47.1,84.7] Years old:5.46E-02(2.87E-01) |
|  | **SM C20:2** | All Participants:-0.08(0.04) Females:-0.12(0.05) Males:-0.01(0.06) [19.3,31.5] Years old:-0.11(0.07) (31.5,47.1] Years old:-0.06(0.07) (47.1,84.7] Years old:-0.07(0.06) | All Participants:3.68E-02(9.84E-02) Females:1.45E-02(9.12E-02) Males:8.86E-01(8.91E-01) [19.3,31.5] Years old:1.03E-01(4.27E-01) (31.5,47.1] Years old:3.93E-01(4.93E-01) (47.1,84.7] Years old:2.88E-01(6.93E-01) |
| Lyso PC | **lysoPC a C20:4** | All Participants:-0.11(0.04) Females:-0.12(0.04) Males:-0.10(0.06) [19.3,31.5] Years old:-0.12(0.06) (31.5,47.1] Years old:-0.10(0.07) (47.1,84.7] Years old:-0.12(0.06) | All Participants:1.82E-03(1.03E-02) Females:5.05E-03(3.92E-02) Males:1.38E-01(2.45E-01) [19.3,31.5] Years old:6.56E-02(3.14E-01) (31.5,47.1] Years old:1.28E-01(2.15E-01) (47.1,84.7] Years old:4.35E-02(2.46E-01) |
|  | **lysoPC a C28:1** | All Participants:0.09(0.03) Females:0.06(0.04) Males:0.16(0.06) [19.3,31.5] Years old:0.11(0.05) (31.5,47.1] Years old:0.16(0.06) (47.1,84.7] Years old:0.01(0.05) | All Participants:2.91E-03(1.53E-02) Females:1.31E-01(3.67E-01) Males:4.96E-03(4.75E-02) [19.3,31.5] Years old:4.09E-02(2.57E-01) (31.5,47.1] Years old:1.01E-02(7.32E-02) (47.1,84.7] Years old:8.38E-01(9.36E-01) |
|  | **lysoPC a C24:0** | All Participants:0.08(0.03) Females:0.06(0.04) Males:0.12(0.05) [19.3,31.5] Years old:0.03(0.05) (31.5,47.1] Years old:0.16(0.05) (47.1,84.7] Years old:0.07(0.05) | All Participants:5.78E-03(2.25E-02) Females:7.96E-02(2.52E-01) Males:2.90E-02(1.18E-01) [19.3,31.5] Years old:6.06E-01(8.45E-01) (31.5,47.1] Years old:4.35E-03(5.91E-02) (47.1,84.7] Years old:2.00E-01(5.93E-01) |
|  | **lysoPC a C28:0** | All Participants:0.08(0.03) Females:0.05(0.04) Males:0.13(0.05) [19.3,31.5] Years old:0.08(0.05) (31.5,47.1] Years old:0.11(0.06) (47.1,84.7] Years old:0.04(0.05) | All Participants:1.48E-02(5.03E-02) Females:2.12E-01(5.20E-01) Males:2.02E-02(9.96E-02) [19.3,31.5] Years old:1.30E-01(4.94E-01) (31.5,47.1] Years old:8.44E-02(1.70E-01) (47.1,84.7] Years old:3.94E-01(7.47E-01) |

Significance level was set to ɑ=0.05. *p*-values obtained from linear mixed-effect models adjusted for covariate stratified by 1) No stratification: age, sex, baseline HRSD_17_ and antidepressant. 2) Sex: age, baseline HRSD_17_ and antidepressant. 3) Age: Sex, baseline HRSD_17_ and antidepressant 4) Drug: age, sex, and baseline HRSD_17_. Metabolites surpassing Benjamini-Hochberg correction (*q*-value<0.05) are bolded.
