# Supplementary material for: Alterations in acylcarnitines, amines, and lipids inform about the mechanism of action of citalopram/escitalopram in major depression
Source: Transl Psychiatry. 2021 Mar 2;11:153. doi: 10.1038/s41398-020-01097-6 (PMC7925685; doi:10.1038/s41398-020-01097-6)
Supplement: Supplementary file 4 — Supplementary Table 3 [file 41398_2020_1097_MOESM4_ESM.docx]

**Supplementary Table 3. Significant Associations Between Change in HRSD_17_ Score and Change in Metabolite Levels Over 8 Weeks of Treatment**

| Class | Metabolite | Effect Size (β) | SE | *p*-value | *q*-value |
| --- | --- | --- | --- | --- | --- |
| **Acylcarnitines** | **C5-M-DC** | -0.203 | 0.087 | 2.09E-02 | 1.94E-01 |
| **Amino Acids** | **Histidine** | -0.188 | 0.086 | 3.09E-02 | 2.33E-01 |
|  | **Proline** | -0.183 | 0.086 | 3.57E-02 | 2.33E-01 |
| **Biogenic Amines** | **Kynurenine** | -0.287 | 0.084 | 8.11E-04 | 6.61E-02 |
|  | **trans-4-Hydroxyproline** | -0.232 | 0.086 | 8.11E-03 | 1.56E-01 |
| **PC aas** | **PC aa C36:3** | -0.245 | 0.085 | 4.52E-03 | 1.55E-01 |
|  | **PC aa C36:2** | -0.242 | 0.085 | 5.35E-03 | 1.55E-01 |
|  | **PC aa C34:2** | -0.236 | 0.085 | 6.43E-03 | 1.55E-01 |
|  | **PC aa C34:1** | -0.216 | 0.086 | 1.28E-02 | 1.67E-01 |
|  | **PC aa C36:4** | -0.215 | 0.086 | 1.33E-02 | 1.67E-01 |
|  | **PC aa C36:1** | -0.195 | 0.085 | 2.39E-02 | 2.05E-01 |
|  | **PC aa C40:2** | -0.186 | 0.086 | 3.21E-02 | 2.33E-01 |
| **PC aes** | **PC ae C38:2** | -0.297 | 0.083 | 5.12E-04 | 6.61E-02 |
|  | **PC ae C36:3** | -0.244 | 0.085 | 4.52E-03 | 1.55E-01 |
|  | **PC ae C34:3** | -0.225 | 0.085 | 9.08E-03 | 1.56E-01 |
|  | **PC ae C36:2** | -0.210 | 0.085 | 1.52E-02 | 1.77E-01 |
|  | **PC ae C34:2** | -0.208 | 0.086 | 1.66E-02 | 1.80E-01 |
|  | **PC ae C38:3** | -0.202 | 0.085 | 1.96E-02 | 1.94E-01 |
|  | **PC ae C36:1** | -0.183 | 0.086 | 3.48E-02 | 2.33E-01 |
| **Lyso PC** | **lysoPC a C18:1** | -0.233 | 0.085 | 6.68E-03 | 1.55E-01 |
|  | **lysoPC a C18:0** | -0.222 | 0.085 | 9.56E-03 | 1.56E-01 |
|  | **lysoPC a C17:0** | -0.213 | 0.085 | 1.33E-02 | 1.67E-01 |
|  | **lysoPC a C18:2** | -0.200 | 0.086 | 2.14E-02 | 1.94E-01 |
|  | **lysoPC a C20:3** | -0.191 | 0.086 | 2.71E-02 | 2.21E-01 |
|  | **lysoPC a C16:0** | -0.183 | 0.085 | 3.43E-02 | 2.33E-01 |

P-values were obtained from linear regression models with change in HRSD_17_ scale as the dependent variable, and independent variable was log_2_ fold change of metabolite concentrations, β represents estimated coefficient of metabolite’s log2 fold change while the models were adjusted for age and sex. Significance level was set to ɑ=0.05.

*Abbreviations*: HRSD_17_: 17-item Hamilton Rating Scale for Depression, PC: Phosphatidylcholine, LysoPC: Lyso-Phosphatidylcholine. Metabolite abbreviations are spelled out in Supplementary Table 1.
