# Supplementary material for: Alterations in acylcarnitines, amines, and lipids inform about the mechanism of action of citalopram/escitalopram in major depression
Source: Transl Psychiatry. 2021 Mar 2;11:153. doi: 10.1038/s41398-020-01097-6 (PMC7925685; doi:10.1038/s41398-020-01097-6)
Supplement: Supplementary file 5 — Supplementary Table 4 [file 41398_2020_1097_MOESM5_ESM.docx]

**Supplementary Table 4. Differences in Metabolites Levels between Remitters and Treatment Failures across Visits**

| **Baseline** | | | **8 weeks** | | |
| --- | --- | --- | --- | --- | --- |
| **Metabolite** | **Estimated Log_2_ Difference (SE)** | ***p*-value(*q*-value)** | **Metabolite** | **Estimated Log_2_ Difference (SE)** | ***p*-value(*q*-value)** |
| **alpha-Aminoadipic acid** | 0.54 (0.26) | 0.040(0.989) | **alpha-Aminoadipic acid** | 0.58(0.26) | 0.027(0.989) |
| **Sarcosine** | 0.57(0.25) | 0.024(0.989) | **Sarcosine** | 0.52(0.25) | 0.038(0.989) |
| **Serotonin** | 1.02(0.48) | 0.032(0.989) | **C5** | 0.38(0.15) | 0.012(0.989) |
| **C3** | 0.35(0.15) | 0.023(0.989) | **lysoPC a C18:2** | 0.34(0.16) | 0.036(0.989) |
| **C5** | 0.31(0.16) | 0.039(0.989) | **lysoPC a C20:4** | 0.28(0.14) | 0.042(0.989) |
|  |  |  | **PC aa C34:1** | 0.09(0.04) | 0.040(0.989) |
|  |  |  | **PC aa C34:2** | 0.10(0.04) | 0.015(0.989) |
|  |  |  | **PC aa C36:2** | 0.10(0.04) | 0.019(0.989) |
|  |  |  | **PC aa C36:4** | 0.10(0.04) | 0.029(0.989) |

Linear mixed effect models were used to assess the significance of visit by treatment outcome interaction. Models were adjusted for age, sex, and baseline HRSD_17_ score. Q-value: Benjamini-Hochberg adjusted p-values. Significance level was set to ɑ(unadjusted)=0.05. N(Remitters)=64; N(Treatment Failures)=12.

*Abbreviations*: LysoPC: Lyso-Phosphatidylcholine. PC: Phosphatidylcholine. Metabolite abbreviations are spelled out in Supplementary Table 1.
