# Supplementary figures and images for: Alterations in acylcarnitines, amines, and lipids inform about the mechanism of action of citalopram/escitalopram in major depression
Source: Transl Psychiatry. 2021 Mar 2;11:153. doi: 10.1038/s41398-020-01097-6 (PMC7925685; doi:10.1038/s41398-020-01097-6)

Supplementary Fig 1. Principal Components Analysis of Amino Acids

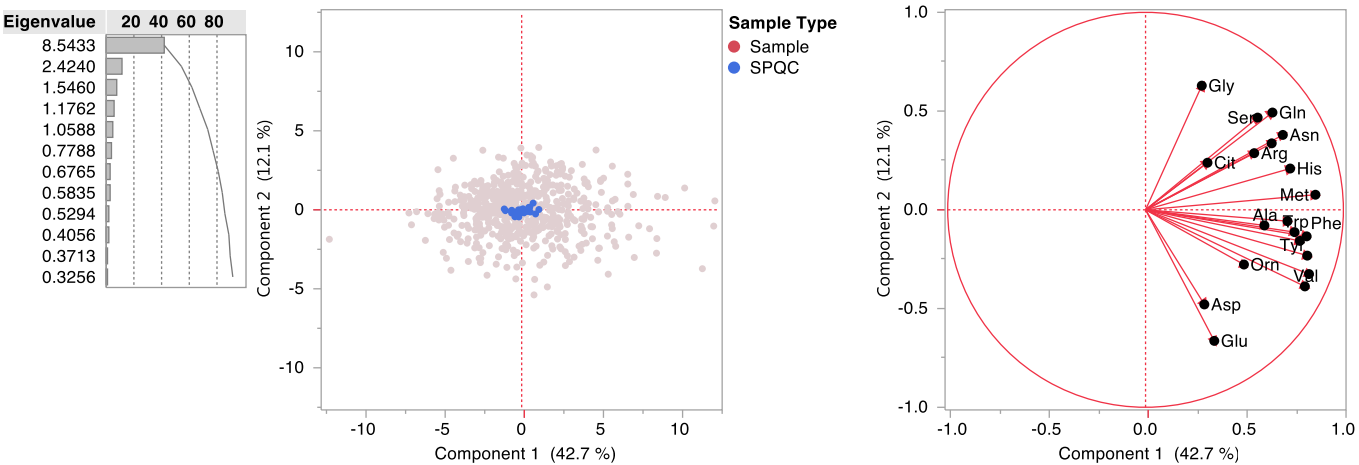

Supplement: Supplementary file 6 — Supplementary Figure 1 [file 41398_2020_1097_MOESM6_ESM.pdf]

Supplementary Fig 2. Principal Components Analysis of Biogenic Amines

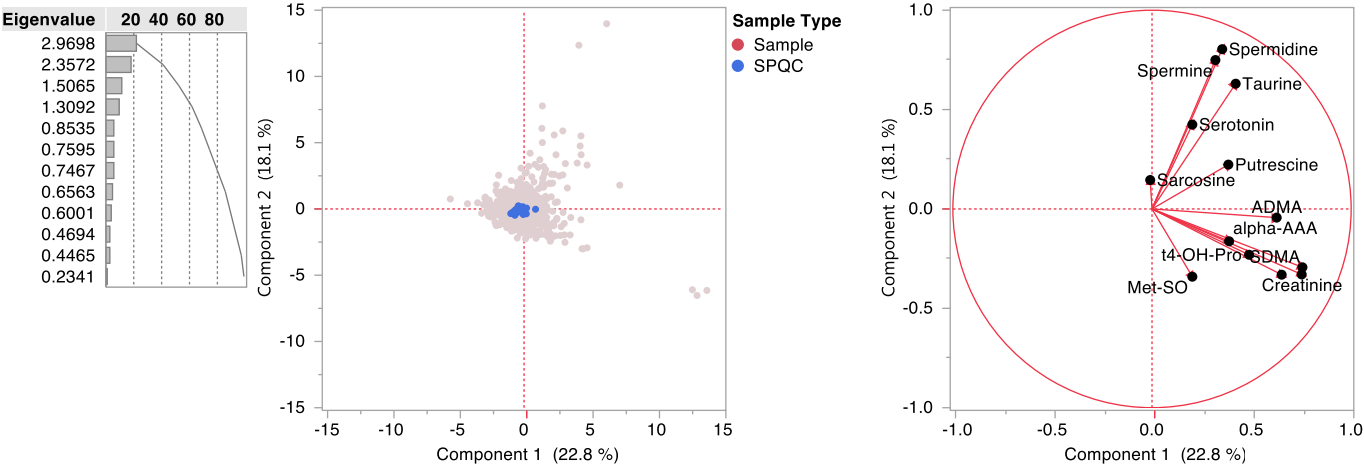

Supplement: Supplementary file 7 — Supplementary Figure 2 [file 41398_2020_1097_MOESM7_ESM.pdf]

Supplementary Fig 3. Principal Components Analysis of Acylcarnitines

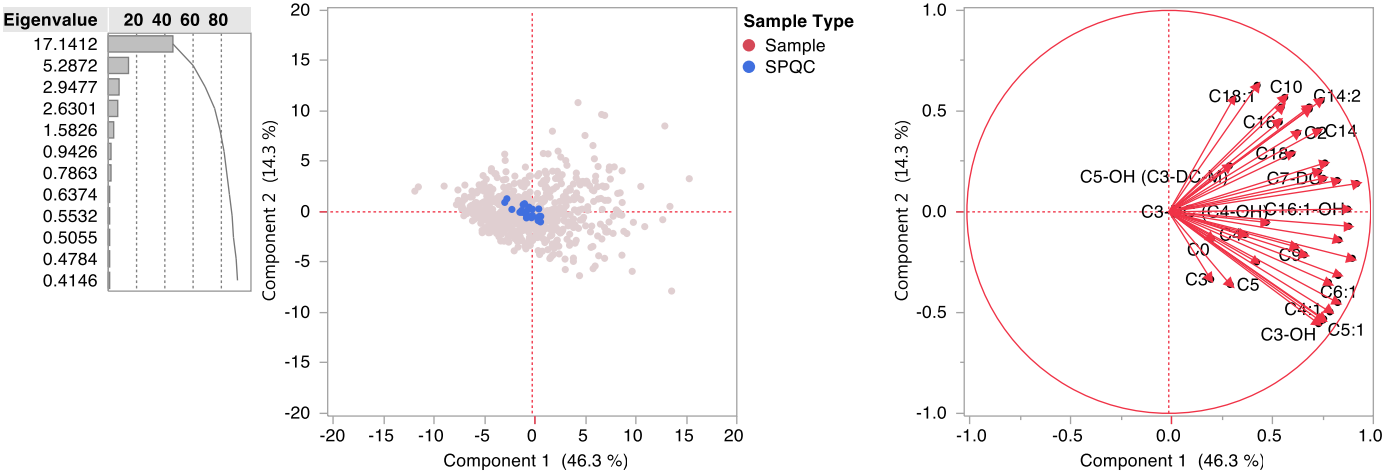

Supplement: Supplementary file 8 — Supplementary Figure 3 [file 41398_2020_1097_MOESM8_ESM.pdf]

Supplementary Fig 4. Principal Components Analysis of Glycerophospholipids

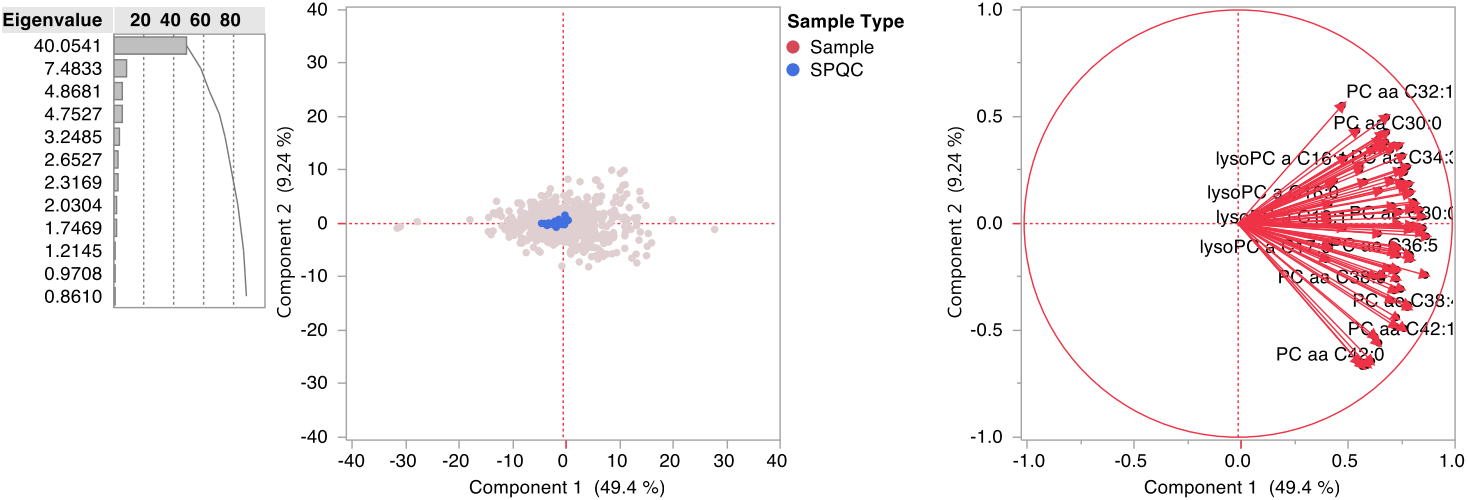

Supplement: Supplementary file 9 — Supplementary Figure 4 [file 41398_2020_1097_MOESM9_ESM.pdf]

Supplementary Fig 5. Principal Components Analysis of Sphingolipids

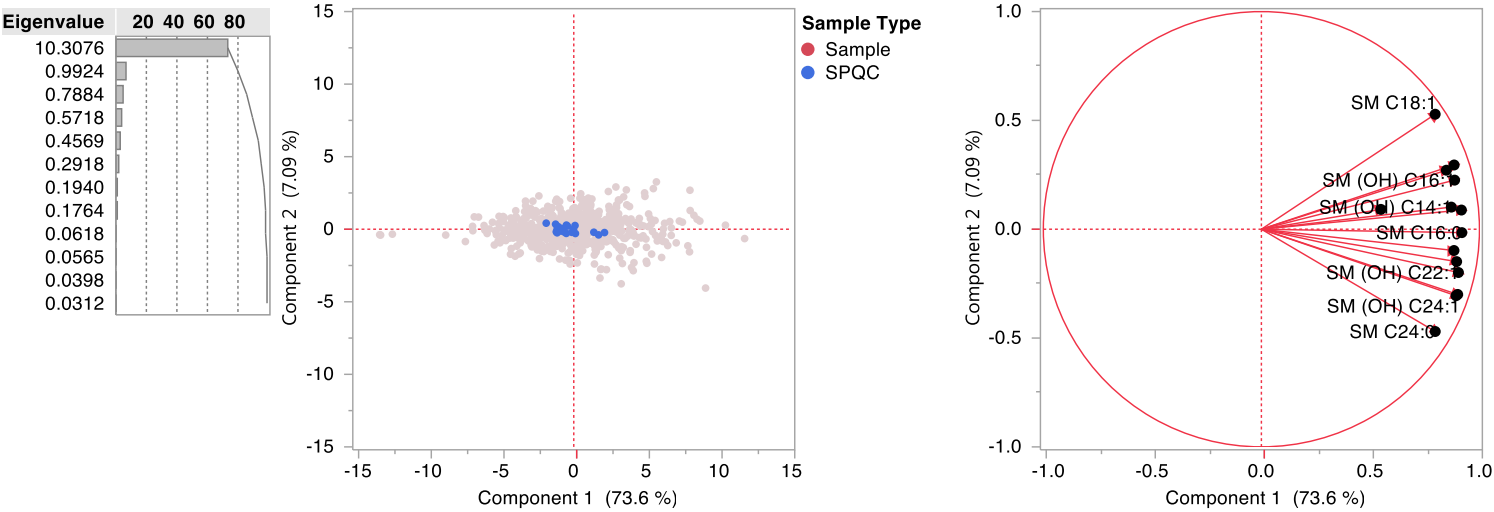

Supplement: Supplementary file 10 — Supplementary Figure 5 [file 41398_2020_1097_MOESM10_ESM.pdf]
