# Supplementary material for: Alterations in acylcarnitines, amines, and lipids inform about the mechanism of action of citalopram/escitalopram in major depression
Source: Transl Psychiatry. 2021 Mar 2;11:153. doi: 10.1038/s41398-020-01097-6 (PMC7925685; doi:10.1038/s41398-020-01097-6)
Supplement: Supplementary file 11 — Supplementary Figure 6 [file 41398_2020_1097_MOESM11_ESM.pdf]

Supplementary Fig 6. Changes in Metabolite Levels after 8 weeks of SSRI Treatment Stratified by Sex, Age and Drug

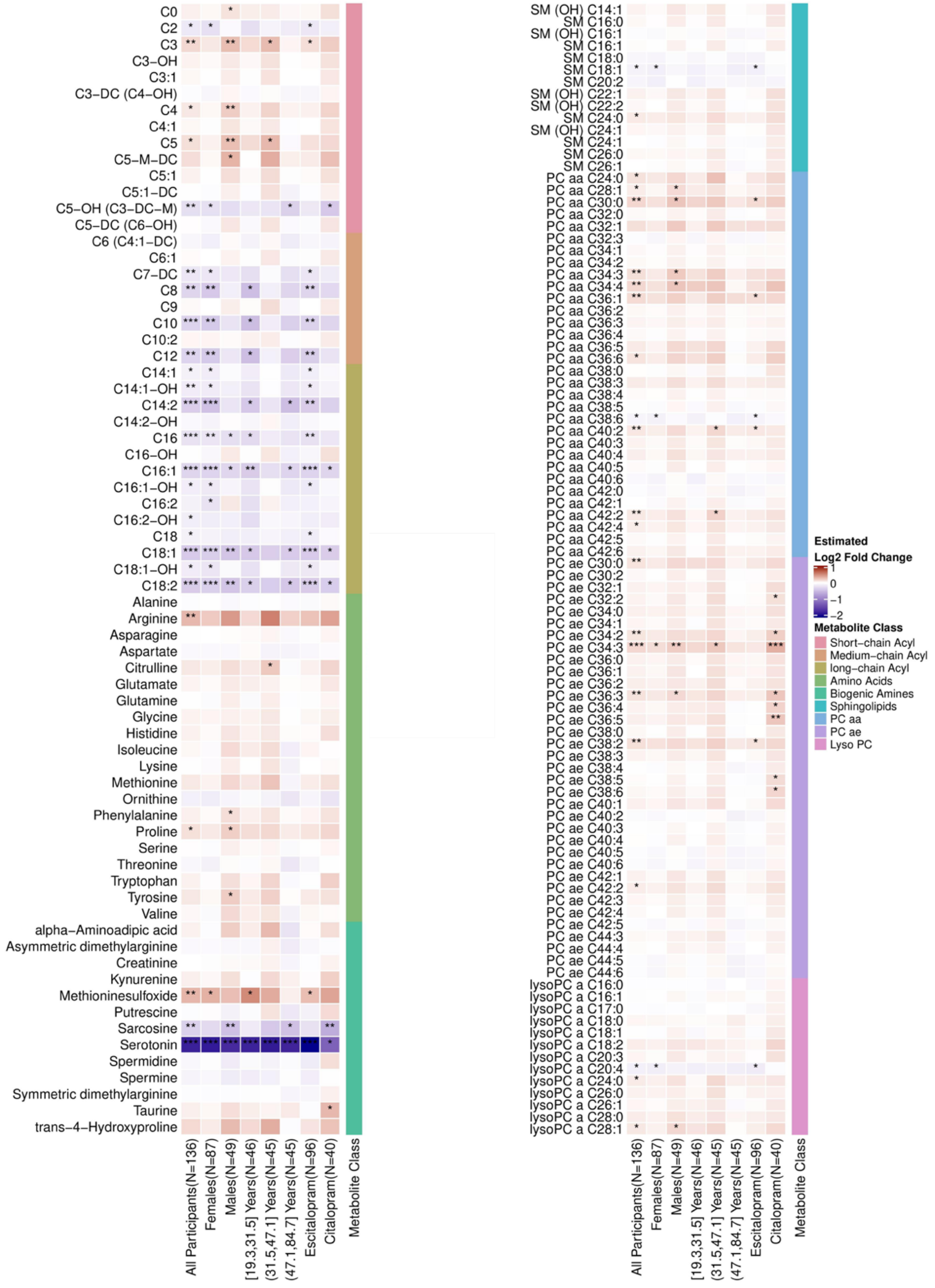

Significance level was set to α=0.05 after Benjamin-Hochberge FDR correction. *p*-values obtained from linear mixed-effect models adjusted for covariate stratified by 1) No stratification: age, sex, baseline $HRSD_{17}$ and antidepressant. 2) Sex: age, baseline $HRSD_{17}$ and antidepressant. 3) Age: Sex, baseline $HRSD_{17}$ and antidepressant 4) Drug: age, sex, and baseline $HRSD_{17}$; ***: q-value<0.001;**: q-value<0.01; *:q<0.05
