# Supplementary material for: Alterations in acylcarnitines, amines, and lipids inform about the mechanism of action of citalopram/escitalopram in major depression
Source: Transl Psychiatry. 2021 Mar 2;11:153. doi: 10.1038/s41398-020-01097-6 (PMC7925685; doi:10.1038/s41398-020-01097-6)
Supplement: Supplementary file 12 — Supplementary Figure 7 [file 41398_2020_1097_MOESM12_ESM.pdf]

Supplementary Fig 7. Effect of 8 Weeks SSRI Treatment on Levels of Ether Phosphatidylcholines.

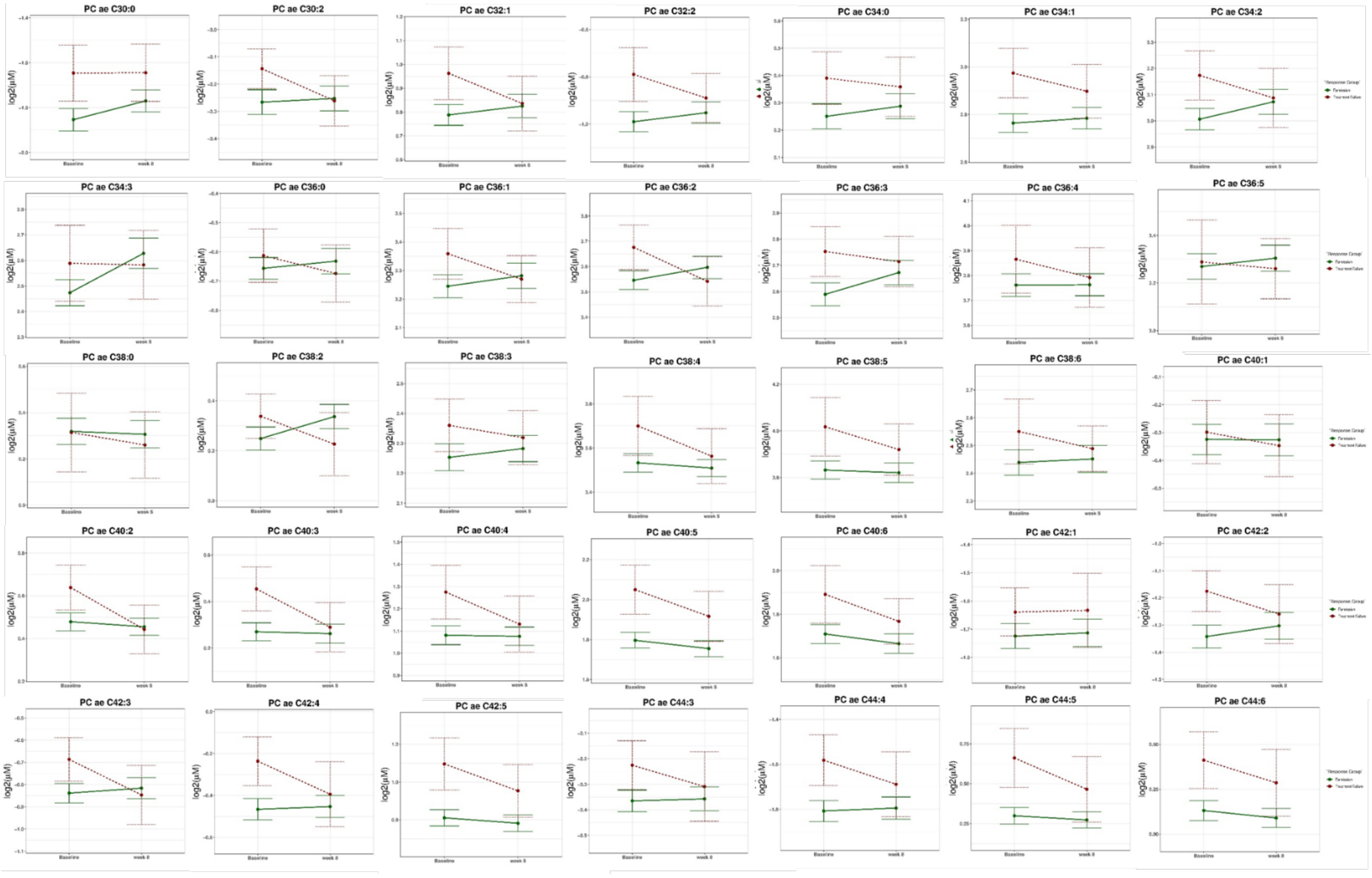

Mean(SE) concentration of etherphosphatidylcholines at baseline and week 8 for the "Remission" and "Treatment failure" groups were calculated. Solid green lines represent "Remission" group and dotted red lines represents "Treatment failure" group.
Abbreviations: SSRI: Selective Serotonin Reuptake Inhibitor, PC: Phosphatidylcholine. Metabolite abbreviations are spelled out in **Supplementary Table 1**.
